# Supplementary material for: Genome reorganisation and expansion shape 3D genome architecture and define a distinct regulatory landscape in coleoid cephalopods
Source: Nat Commun. 2026 Oct 9;17:10319. doi: 10.1038/s41467-026-77625-7 (PMC13652810; doi:10.1038/s41467-026-77625-7)
Supplement: Supplementary file 1 [file 41467_2026_77625_MOESM1_ESM.pdf]

Supplementary Information

Table of contents

Supplementary Figures **..2**

Supplementary Notes **..32**

Supplementary Note 1. Evolutionary braiding entropy reflects chromatin interaction conservation **..32**

Supplementary Note 2. Interaction conservation and ancestral genome organisation shape tissue-wide gene expression and reflect regulatory constraints **..........................33**

Supplementary Note 3. Functional enrichment patterns reflect interaction conservation and ancestral chromosomal context of gene pairs **..........................35**

Supplementary Note 4. Loop-associated genes are enriched for dynamic regulatory functions and transcription binding factor motifs across species and contexts **..........................37**

Supplementary Note 5. **Chromatin loops are enriched for CNEs and open chromatin regions and show greater plasticity than TADs 39**

Supplementary References **..41**

Supplementary Figures

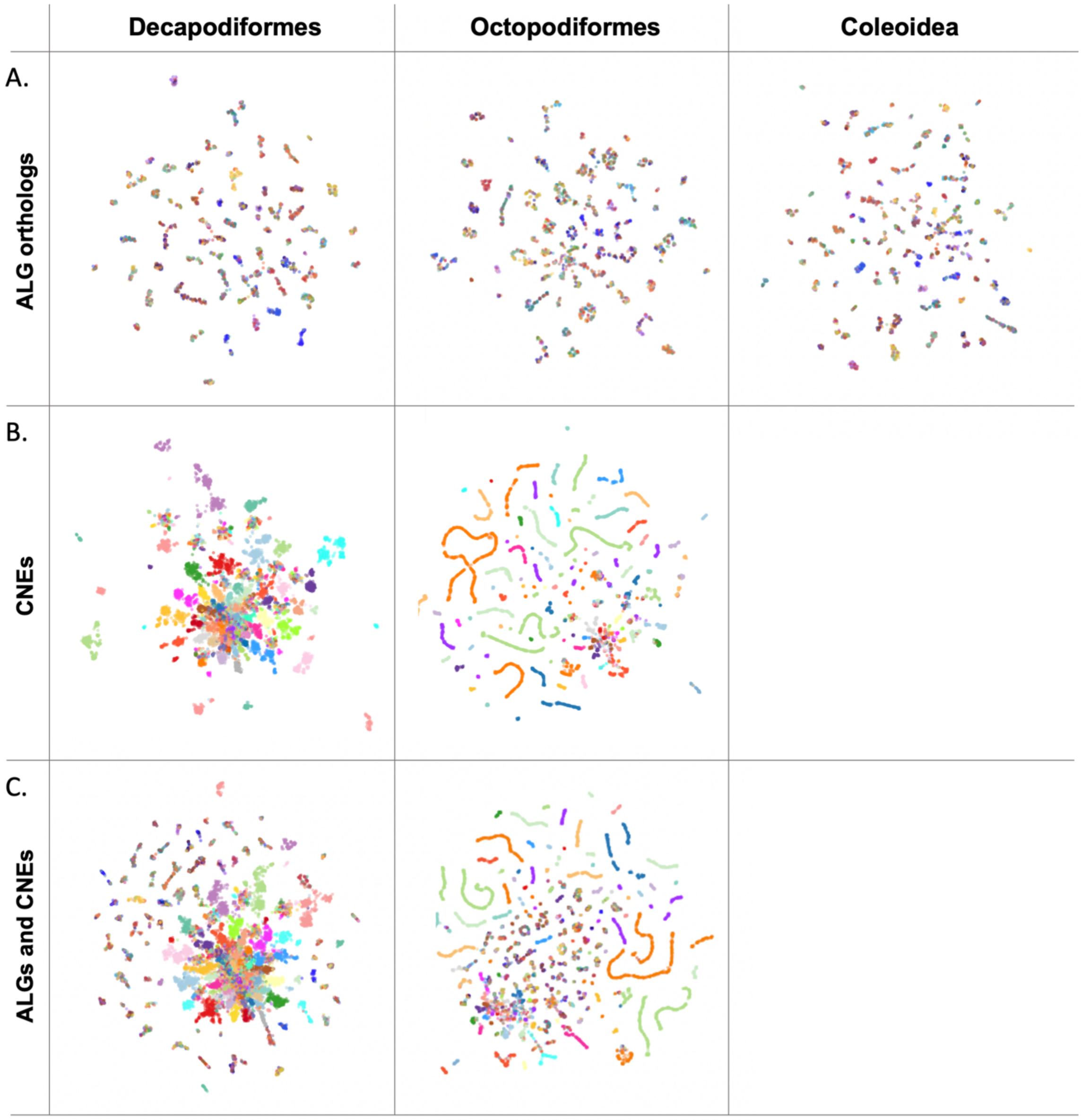

Figure S1. Multi-locus topology of ALG orthologs and conserved non-coding elements (CNEs) across coleoid cephalopods.

Each point represents a genomic locus, coloured by either: (A) ALG orthologs identified in BCnS[1], or (B, C) by chromosome of origin: *E. scolopes* chromosomes for Decapodiform CNEs and *O. bimaculoides* chromosomes for Octopodiform CNEs. UMAP was performed using 15 nearest neighbours and a minimum distance of 0.75.

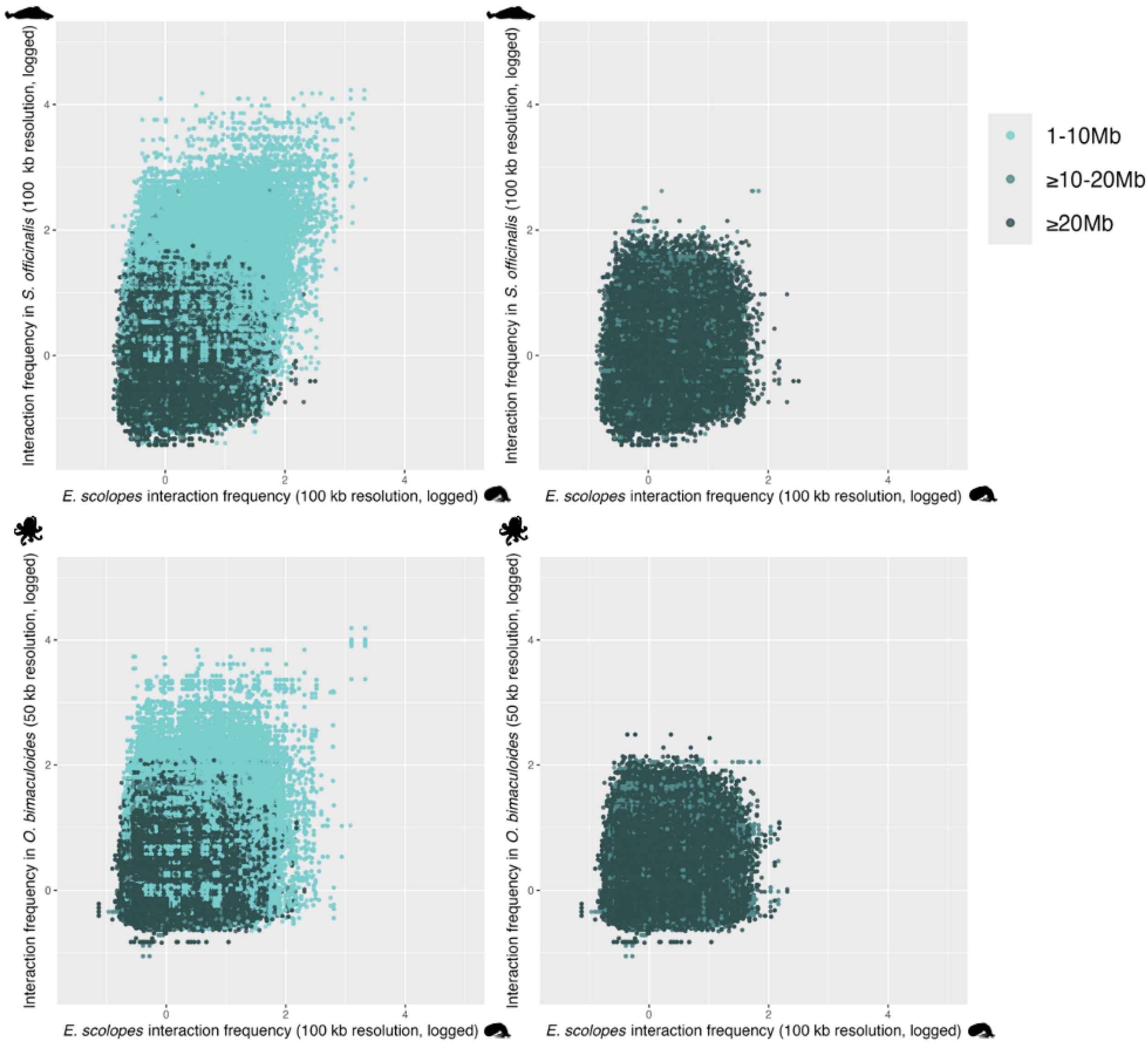

Figure S2. Justification of interaction frequency threshold based on distance-dependent signal decay across species.
Pairwise interaction frequencies (log-transformed) are plotted between *E. scolopes* (x-axis) and either *S. officinalis* or *O. bimaculoides* (y-axis) at 100 kb or 50 kb resolution, respectively, for orthologous gene pairs. Points are coloured by genomic distance: 1–10 Mb (light blue), 10–20 Mb (medium blue), and ≥20 Mb (dark blue). The right-hand panels show the same data with interactions spanning 1–10 Mb removed. Interactions involving regions ≥20 Mb apart rarely exceed 9 KR-normalised reads, providing a basis for setting the threshold of ≥10 reads to define interacting gene pairs. This threshold captures topological interactions primarily within 20 Mb and minimises inclusion of artefacts due to long-range noise or misassemblies. Source data are provided as a Source Data file.

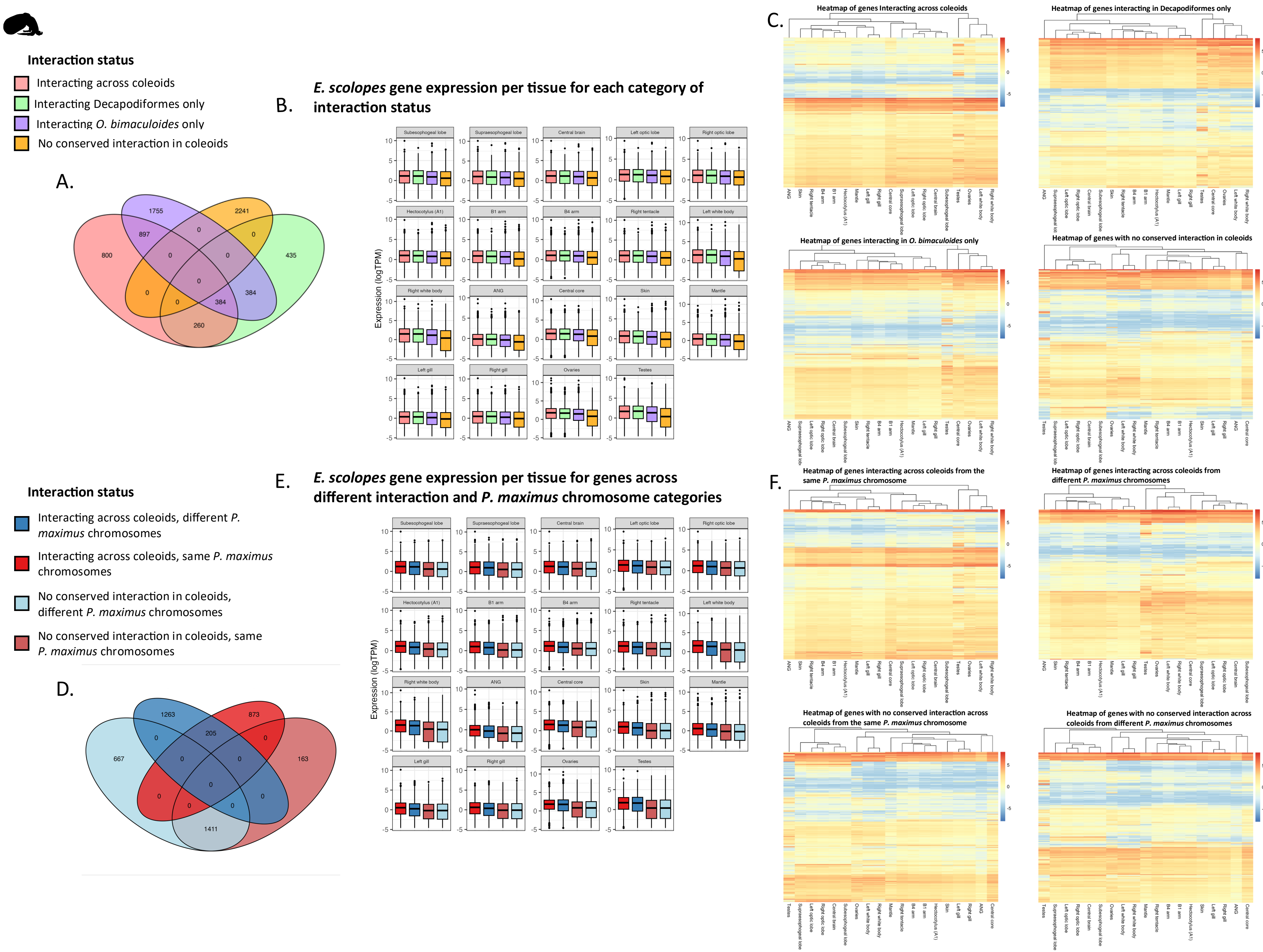
Interaction status
Interacting across coleoids
Interacting Decapodiformes only
Interacting O. bimaculoides only
No conserved interaction in coleoids
A.
B.
E. scolopes gene expression per tissue for each category of interaction status
Expression (logTPM)
C.
Heatmap of genes Interacting across coleoids
Heatmap of genes interacting in Decapodiformes only
Heatmap of genes interacting in O. bimaculoides only
Heatmap of genes with no conserved interaction in coleoids
Interaction status
Interacting across coleoids, different P. maximus chromosomes
Interacting across coleoids, same P. maximus chromosomes
No conserved interaction in coleoids, different P. maximus chromosomes
No conserved interaction in coleoids, same P. maximus chromosomes
D.
E.
E. scolopes gene expression per tissue for genes across different interaction and P. maximus chromosome categories
F.
Heatmap of genes interacting across coleoids from the same P. maximus chromosome
Heatmap of genes interacting across coleoids from different P. maximus chromosomes
Heatmap of genes with no conserved interaction across coleoids from the same P. maximus chromosome
Heatmap of genes with no conserved interaction across coleoids from different P. maximus chromosomes

Figure S3. Expression patterns of *E. scolopes* genes across tissues in relation to interaction conservation and ancestral chromosomal organisation.
(A) Venn diagram showing the overlap of genes across interaction categories included in gene expression analyses. Only genes unique to the 'no conserved interaction in coleoids' category were retained for comparisons in analyses of single genes (i.e. not paired).
(B) Boxplots showing log-transformed TPM expression of *E. scolopes* genes across tissues, grouped by interaction status: interacting in across coleoids (pink), Decapodiformes only (green), *O. bimaculoides* only (purple), or no conserved interaction in coleoids (orange). Sample sizes for each interaction category are shown in panel A. Two-sided Wilcoxon rank-sum test significance values comparing interaction categories within each tissue are provided in Supplementary Data 3.
(C) Heatmaps of log-transformed TPM expression across *E. scolopes* tissues for genes in each interaction category.
(D) Venn diagram showing the overlap of genes across combined interaction and *P. maximus* chromosome status categories included in the expression analyses. Again, only genes unique to the 'no conserved interaction in coleoids' category were retained for comparisons, as in all analyses of single genes (i.e. not paired).
(E) Boxplots showing log-transformed TPM expression across *E. scolopes* tissues for genes interacting across coleoids or with no conserved interaction in coleoids, grouped by *P. maximus* chromosome status (same chromosome, interacting: dark red; same chromosome, non-interacting: light red; different chromosome, interacting: dark blue; different chromosome, non-interacting: light blue). Sample sizes for each category are shown in panel D. Two-sided Wilcoxon rank-sum test significance values comparing interaction categories within each tissue are provided in Supplementary Data 6.
(F) Heatmaps showing log-transformed TPM expression across *E. scolopes* tissues for genes interacting in all species or not interacting in any species, grouped by *P. maximus* chromosome status.
Source data are provided as a Source Data file.

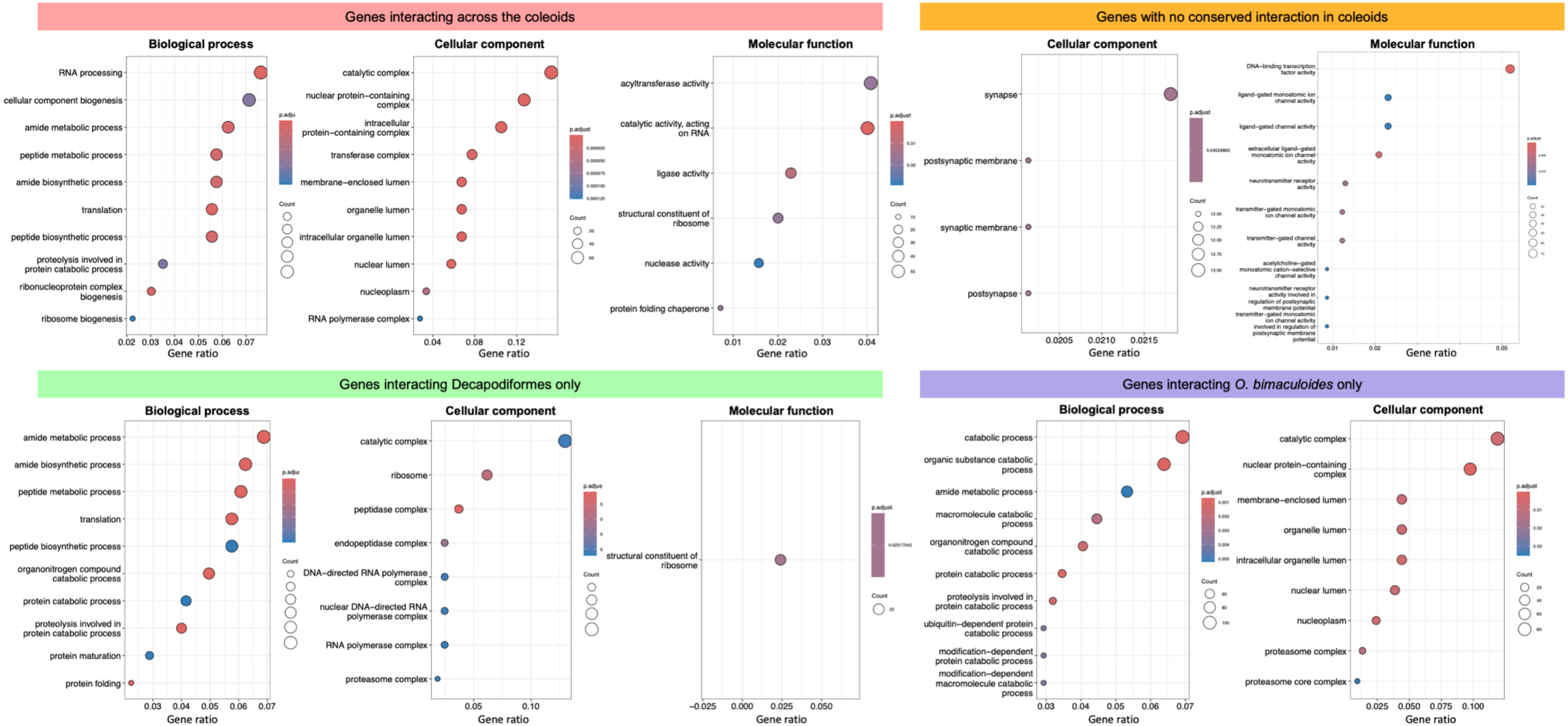

Figure S4. Gene Ontology (GO) enrichment analysis for genes in each interaction category.
Dot plots showing enriched GO terms (biological process, cellular component, molecular function) for gene pairs grouped by interaction status: Genes interacting in across the coleoids (pink), genes with no conserved interaction in coleoids (orange), genes interacting in Decapodiformes only (green), genes interacting only in *O. bimaculoides* (purple). For each GO category, enriched GO terms are shown along the y-axis. Dot size indicates the number of genes associated with each term, and colour reflects statistical significance (adjusted P value). The gene ratio, shown on the x-axis, represents the proportion of input genes annotated with each GO term (number of genes in the term divided by total number of genes in the input list). Source data are provided as a Source Data file.

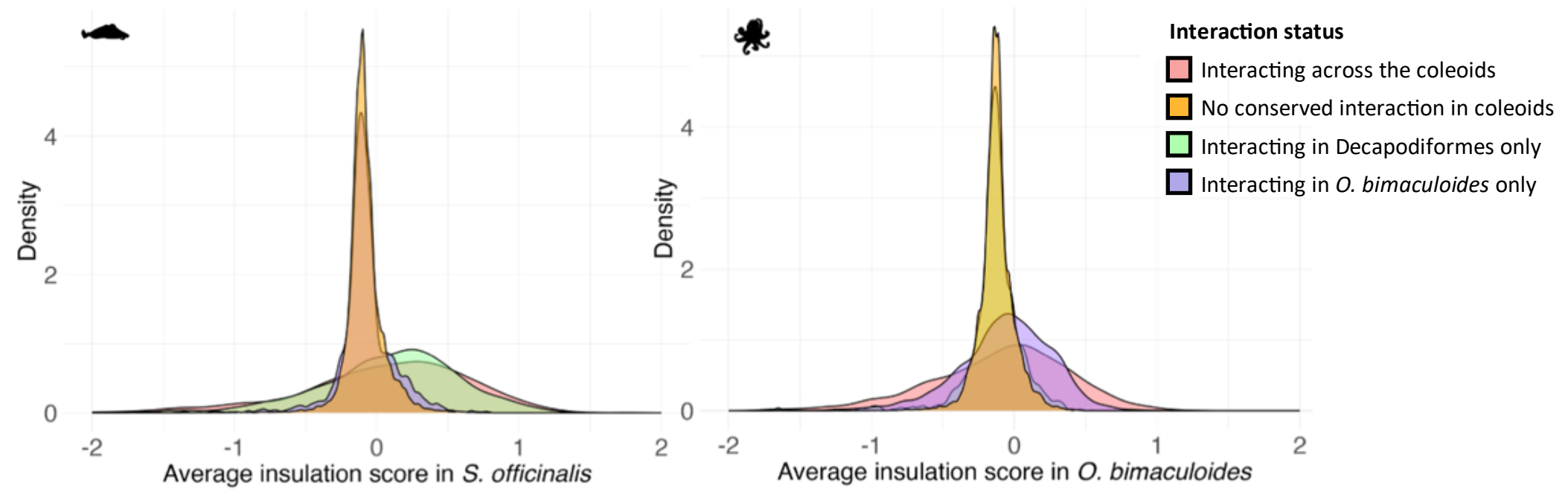

Figure S5. **Insulation score distributions for gene pairs grouped by interaction conservation.**
Density plots of average insulation scores between gene pairs in *S. officinalis* (left) and *O. bimaculoides* (right), grouped by interaction status across species. Insulation scores were computed from Micro-C data at 100 kb resolution using a 350 kb window in *S. officinalis* and at 50 kb resolution with a 350 kb window in *O. bimaculoides*. All within-species pairwise comparisons were significant (BH-corrected two-sided Welch's t-test, P < 0.05), except comparisons between the categories 'no conserved interaction in coleoids' vs. 'interacting in *O. bimaculoides* only' and 'interacting across the coleoids' vs. 'interacting in Decapodiformes only' in *S. officinalis* and 'interacting across the coleoids' vs. 'interacting in *O. bimaculoides* only', in *O. bimaculoides*. Source data are provided as a Source Data file.

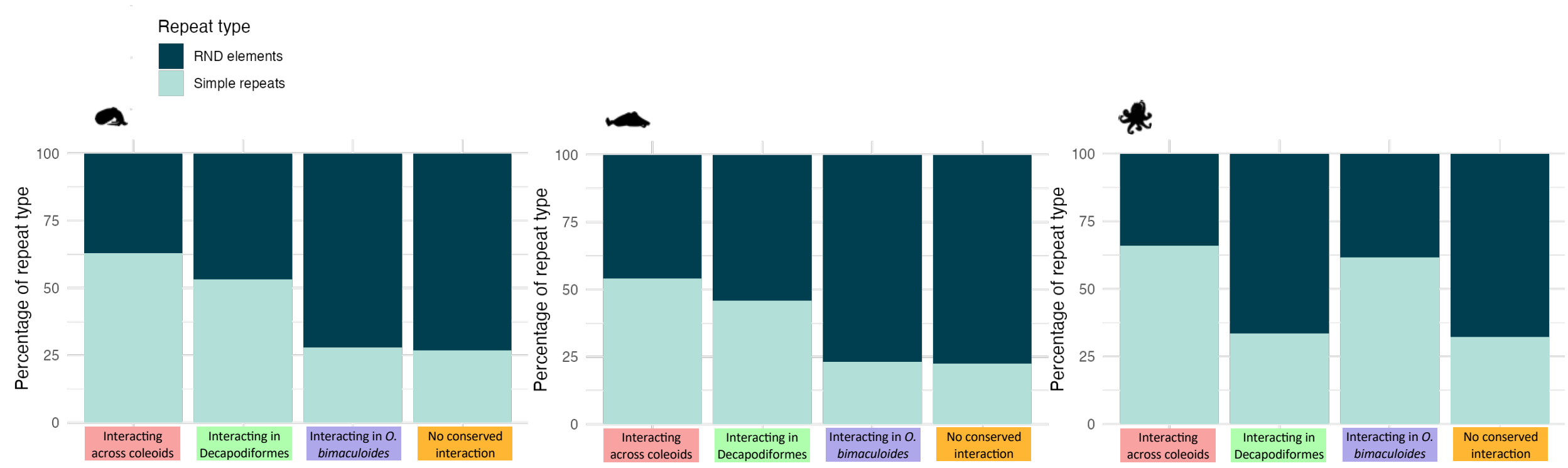

Figure S6. **Repeat content for gene pairs grouped by interaction conservation.**
Stacked bar plots showing the percentage of RND elements (complex repeats identified by RepeatModeler) and simple repeats found in the genomic intervals between gene pairs across three coleoid species (*E. scolopes*, *S. officinalis*, and *O. bimaculoides*). Gene pairs are grouped by interaction status. Across all species and categories, RND elements constitute the majority of repeat content. Differences in repeat composition in Decapodiformes and *O. bimaculoides* likely reflects variation in genome architecture associated with interaction conservation and lineage specificity. Source data are provided as a Source Data file.

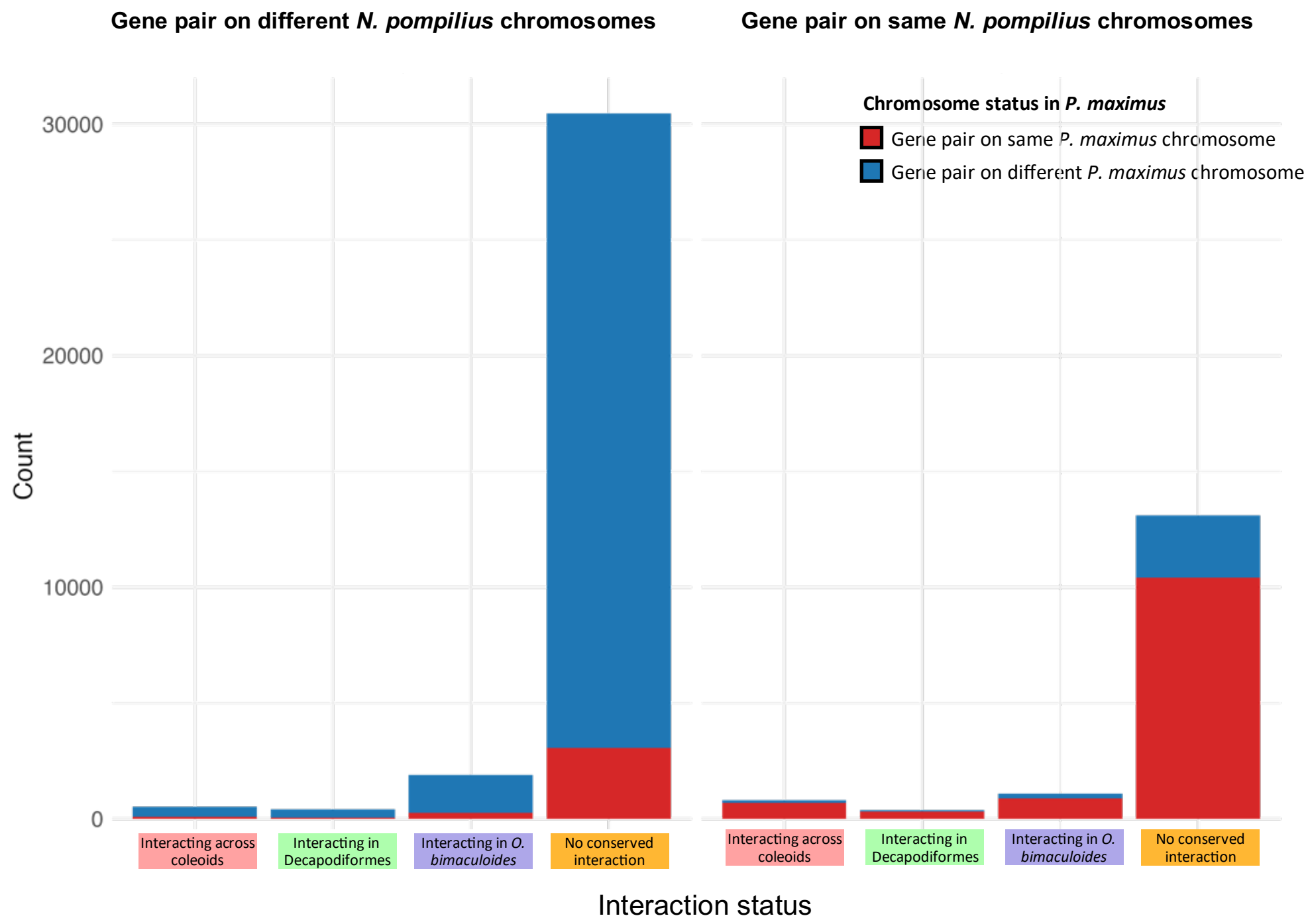

Figure S7. Chromosomal arrangement of interacting gene pairs in *N. pompilius* and *P. maximus*.
Barplots showing the number of gene pairs grouped by interaction status (x-axis), split by whether they are located on different (left) or the same (right) *N. pompilius* chromosomes. Bars are coloured by whether the gene pairs are on different (blue) or the same (red) *P. maximus* chromosomes. Across all interaction categories, 83–87% of gene pairs show concordant chromosome assignments between the two genomes, indicating broadly consistent large-scale chromosomal organisation between *N. pompilius* and *P. maximus*. Source data are provided as a Source Data file.

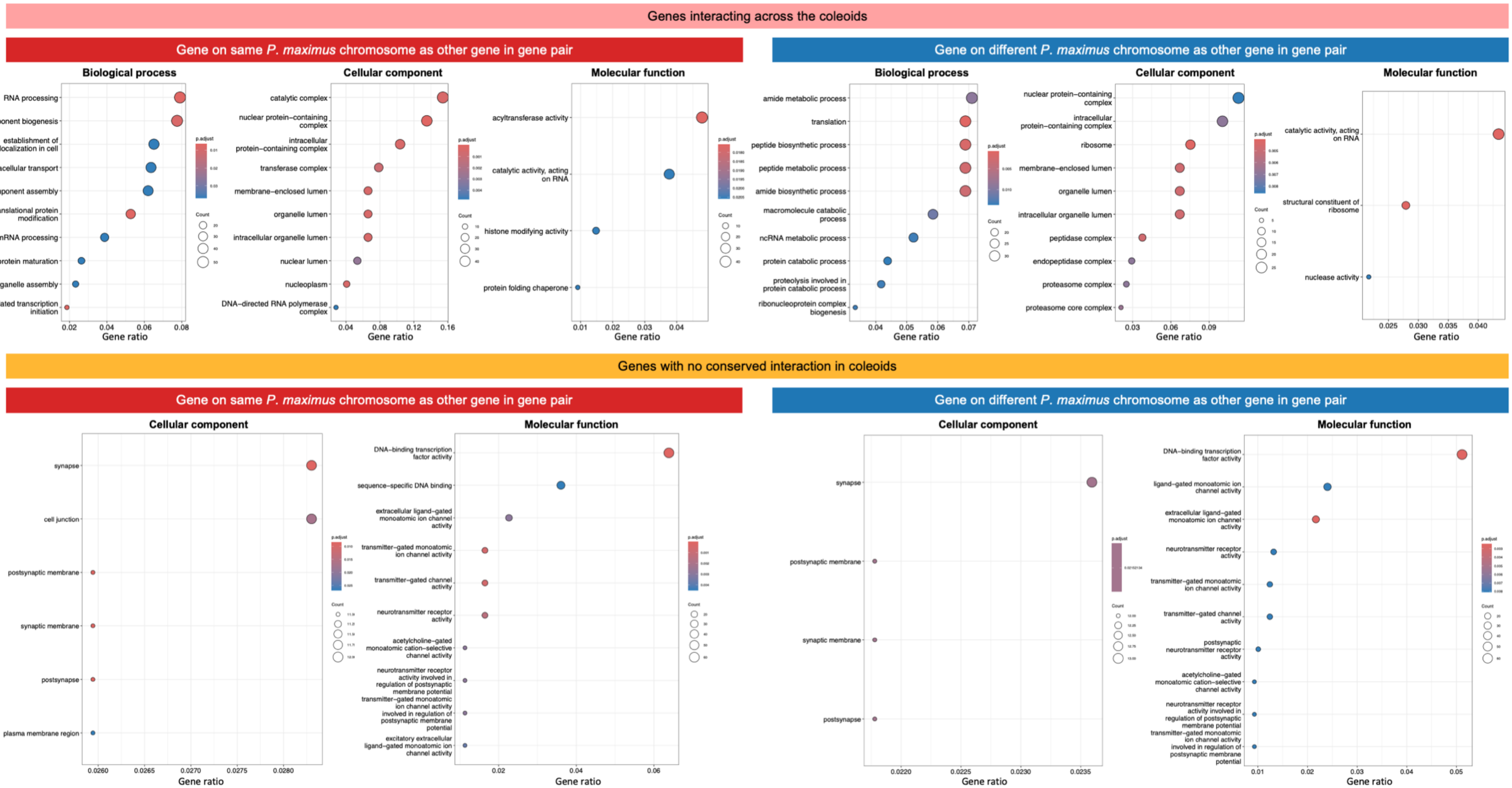

Figure S8. Gene Ontology (GO) enrichment analysis for *E. scolopes* genes grouped by interaction status and chromosomal context in *P. maximus*.
Dot plots show enriched GO terms (biological process, cellular component, and molecular function) for genes interacting across the coleoids (top panels; pink) and for genes with no conserved interaction in coleoids (bottom panels; orange). Genes are further separated by whether their gene pairs are located on the same (left; red) or different (right; blue) *P. maximus* chromosome as the other gene in the pair. For each group, enriched GO terms are displayed along the y-axis. Dot size represents the number of genes annotated with the term; colour reflects statistical significance (adjusted P value); and the x-axis shows the gene ratio, that is, the proportion of input genes annotated with the given GO term. Source data are provided as a Source Data file.

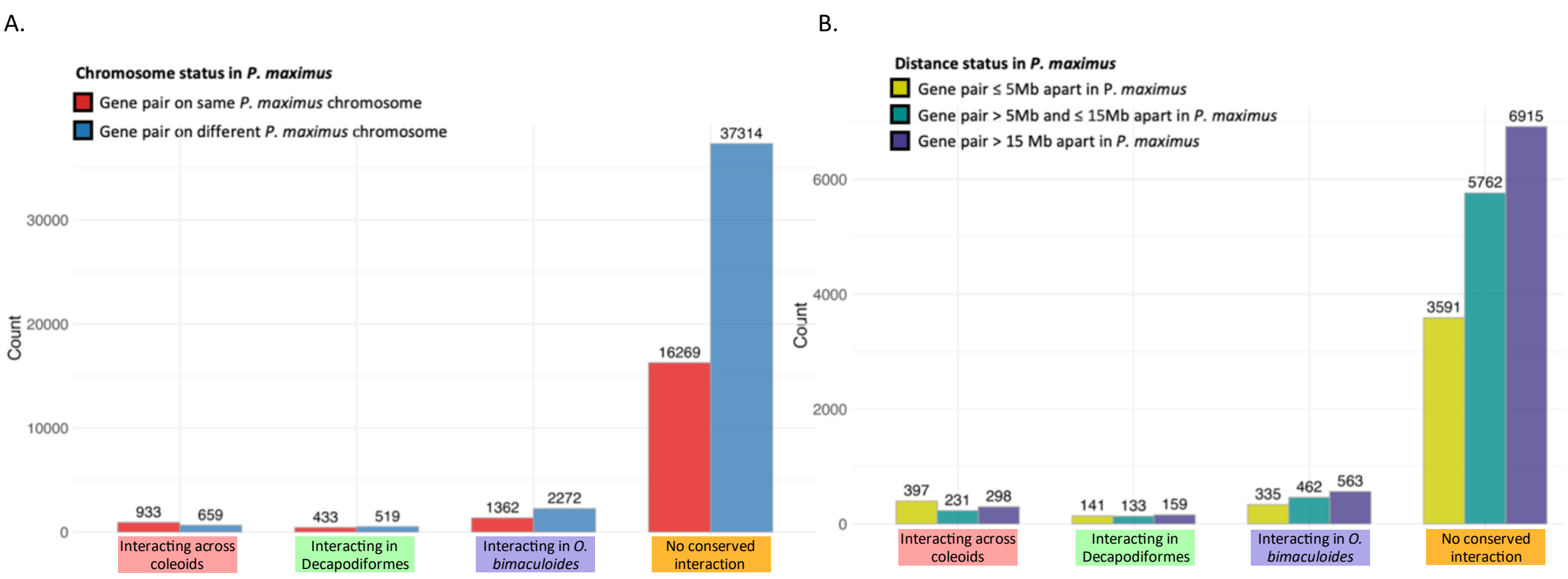

Figure S9. Chromosomal and genomic distance context of gene pairs stratified by interaction status.
Bar plots showing the number of gene pairs in each interaction category, coloured by their chromosomal arrangement in *P. maximus*. Gene pairs are categorised as being located as (A) on the same (red) or different (blue) *P. maximus* chromosomes or (B) ≤ 5 Mb (yellow), > 5 Mb and ≤ 15 Mb (green), and > 15 Mb (purple) apart on the same *P. maximus* chromosomes. Source data are provided as a Source Data file.

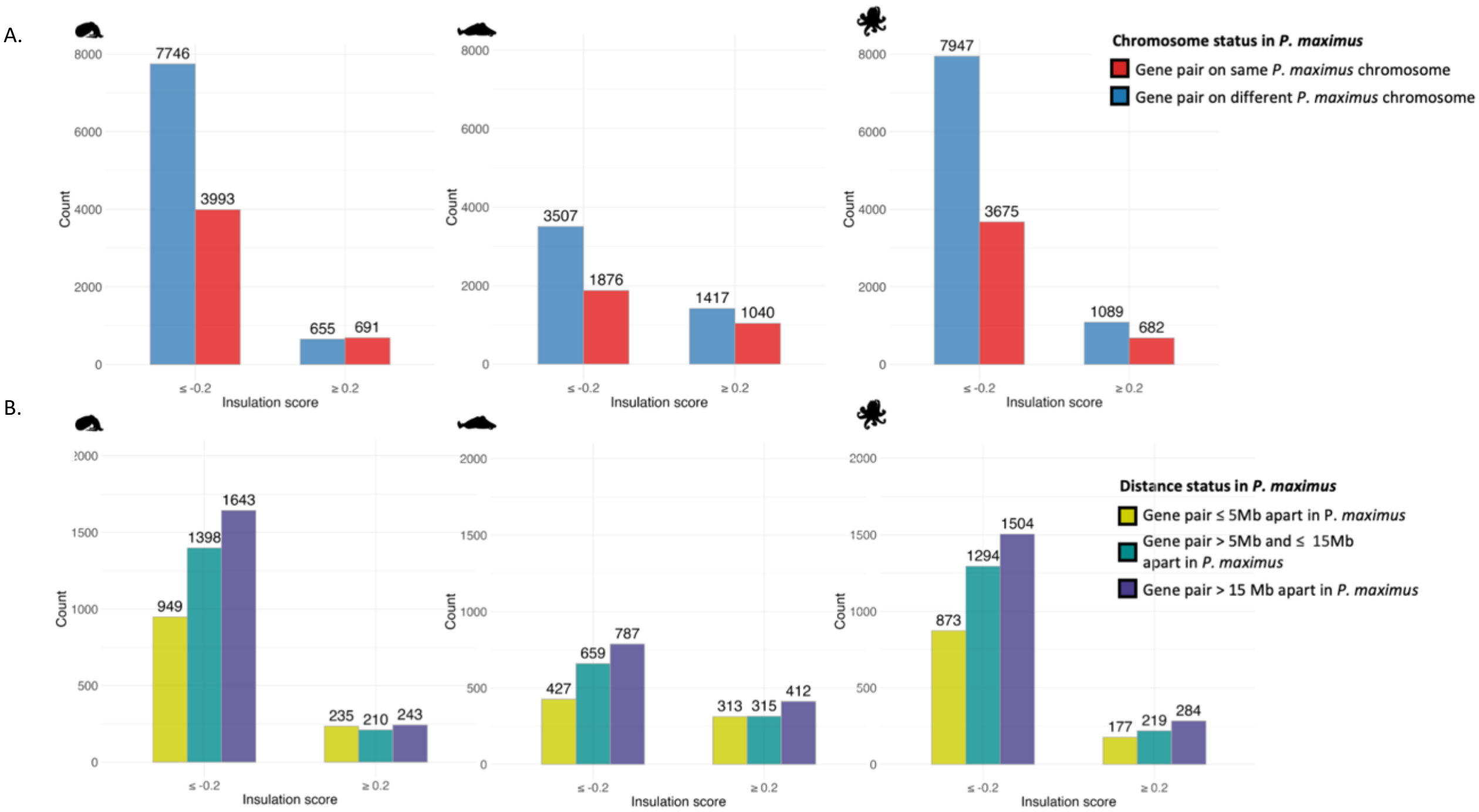

Figure S10. Chromosomal and genomic distance context of gene pairs stratified by insulation score and species.
Bar plots showing the number of gene pairs with low (≤ −0.2) or high (≥ 0.2) average insulation scores in *E. scolopes*, *S. officinalis*, and *O. bimaculoides*. Insulation scores were computed from Micro-C data at 100 kb resolution using a 350 kb window in *S. officinalis* and at 50 kb resolution with a 350 kb window in *O. bimaculoides.* Gene pairs are categorised according to (A) whether their orthologs are located on the same (red) or different (blue) *P. maximus* chromosomes, or (B) the linear genomic distance between orthologs in *P. maximus*: ≤ 5 Mb (yellow), > 5 Mb and ≤ 15 Mb (green), and > 15 Mb (purple). Source data are provided as a Source Data file.

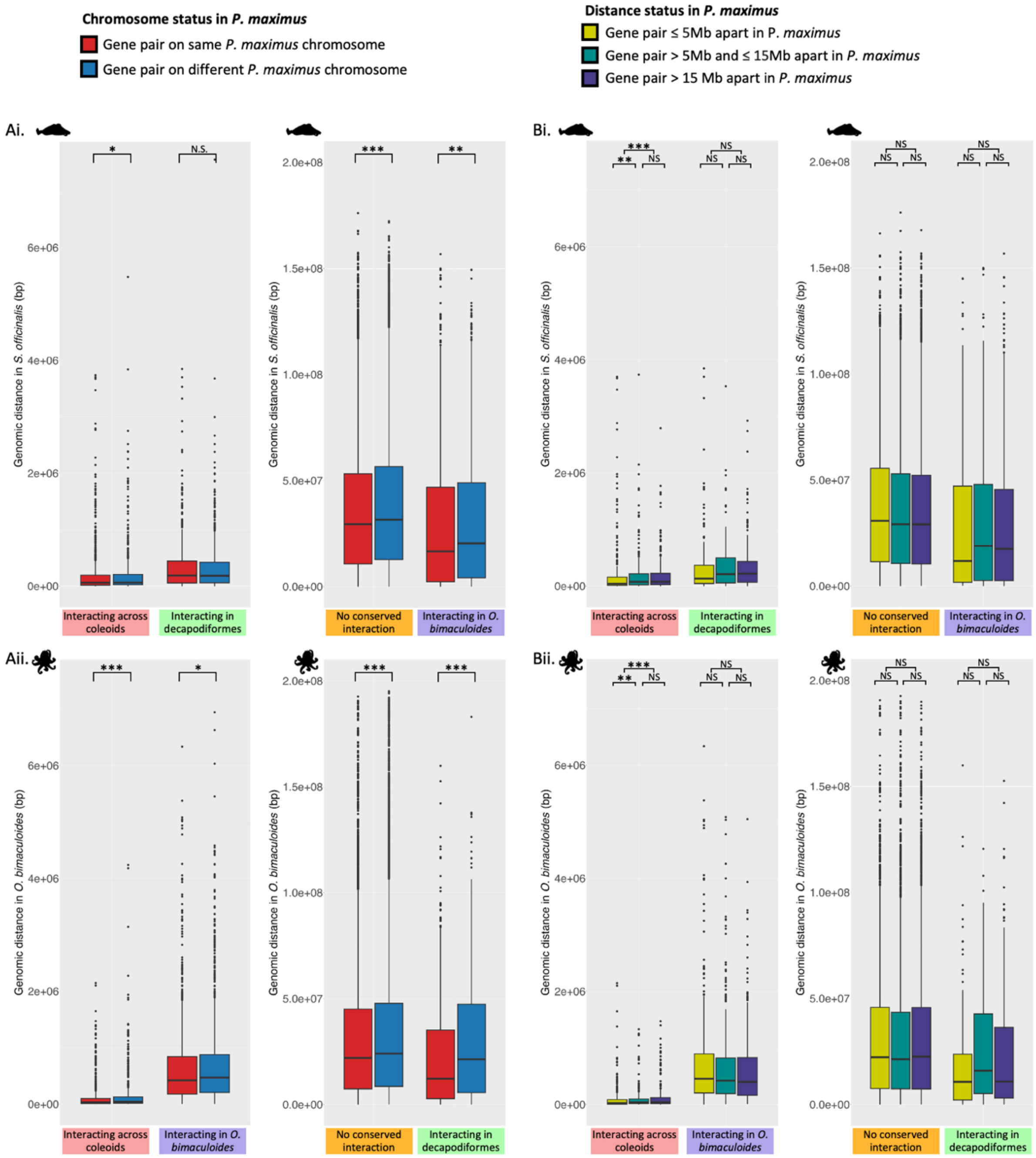

Figure S11. Genomic distances between gene pairs in *S. officinalis* and O. *bimaculoides*, grouped by interaction status and ancestral genomic context in *P. maximus*.

(Ai) Genomic distances between gene pairs in *S. officinalis*, coloured by ancestral chromosome status in *P. maximus* (same chromosome: red; different chromosomes: blue), grouped by interaction status (interacting across coleoids vs. interacting in decapodiformes; no conserved interaction vs. interacting in *O. bimaculoides*).

(Aii) As in (Ai), but for genomic distances between gene pairs in *O. bimaculoides*.

(Bi) Genomic distances between gene pairs in *S. officinalis*, coloured by ancestral genomic distance in *P. maximus* (≤5 Mb apart: yellow; >5–15 Mb apart: turquoise; >15 Mb apart: purple), grouped by interaction status as in (Ai).

(Bii) As in (Bi), but for genomic distances between gene pairs in *O. bimaculoides*.

Boxplots were generated from orthologous gene pairs, and sample sizes for each group are provided in Fig. S9. Boxplot significance values are based on BH-corrected two-sided Wilcoxon rank-sum tests: NS = non-significant, * = P < 0.05, ** = P < 0.005, *** = P < 0.0005. Exact P values are provided in Supplementary Data 7Aii and Bii.

Centre line, median; box limits, upper and lower quartiles; whiskers, 1.5 × interquartile range; points represent outlying gene pairs. Source data are provided as a Source Data file.

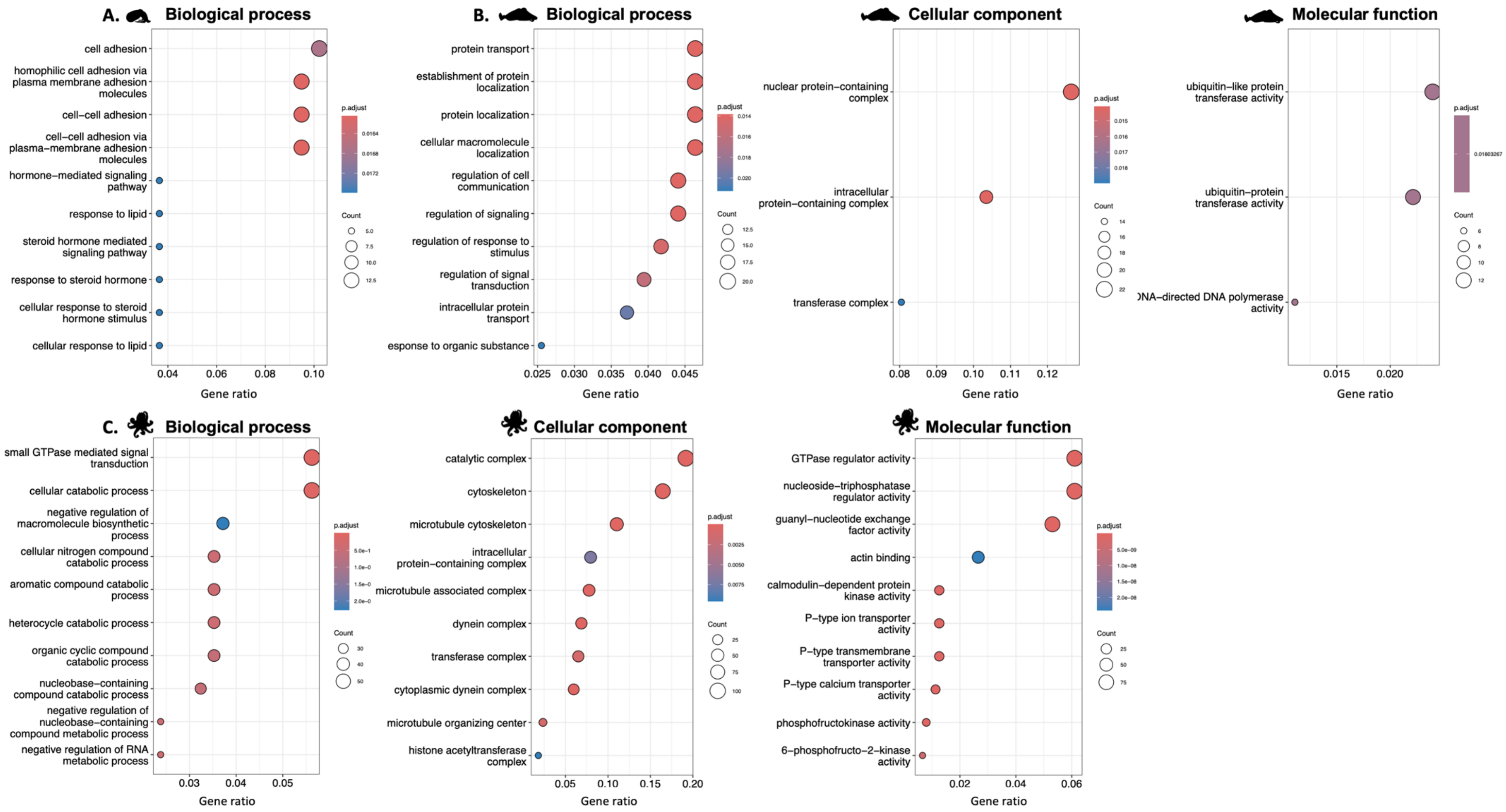

Figure S12. GO term enrichment results for genes located in loop anchors across whole-embryo datasets in *E. scolopes*, *O. bimaculoides*, and *S. officinalis*. (A) Enriched Gene Ontology (GO) terms for loop anchor genes in *E. scolopes*, (B) *O. bimaculoides*, and (C) *S. officinalis*, grouped by GO category: biological process, cellular component, and molecular function. Each dot represents an enriched GO term, dot size indicates the number of genes associated with each term, and colour reflects statistical significance (adjusted P value). Gene ratio is defined as the number of genes in the query set annotated with a given term divided by the total number of query genes. Only genes located at loop anchors in loops detected at 100 kb and 50 kb resolution were included in the analysis. Source data are provided as a Source Data file.

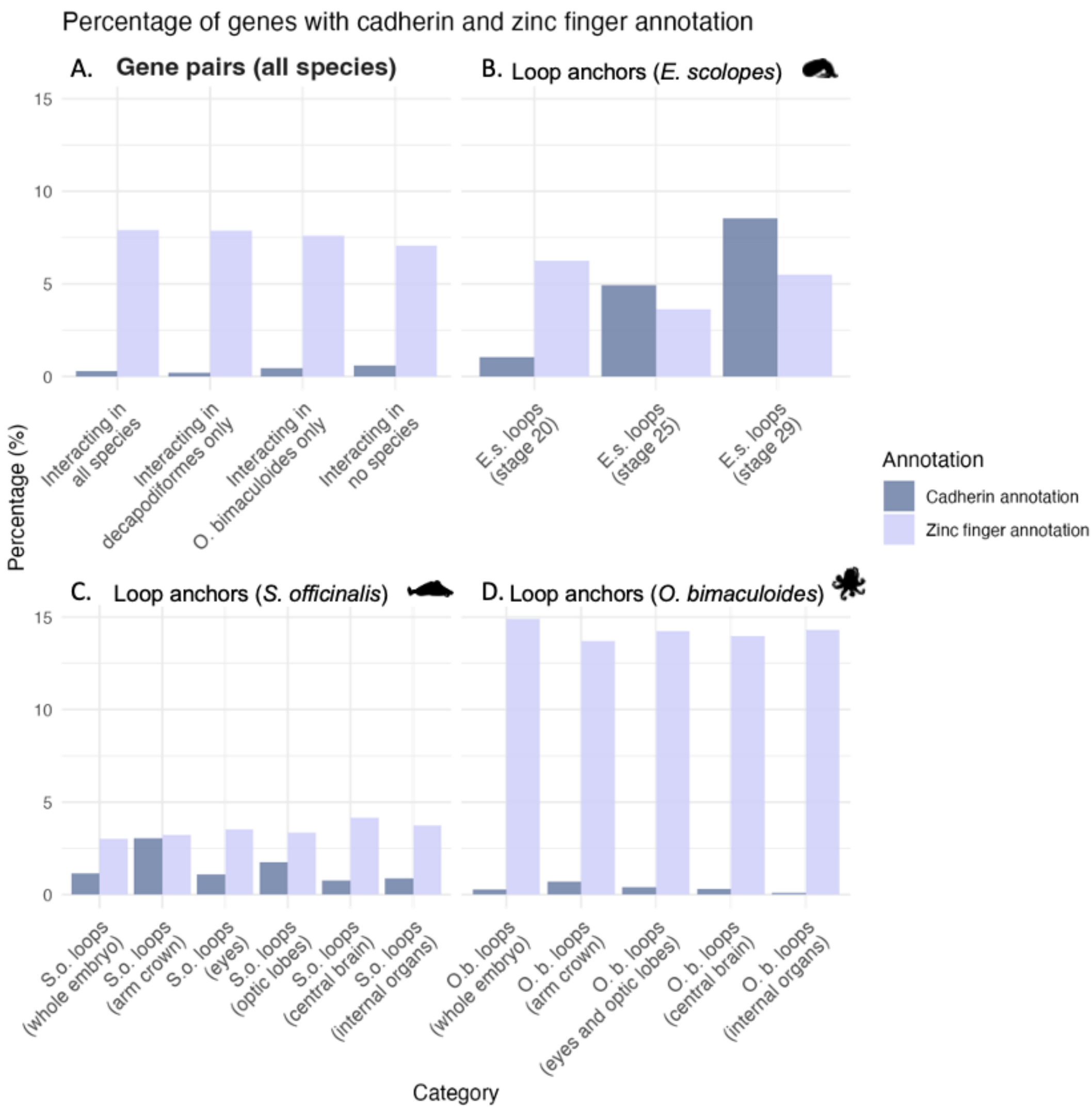

Figure S13. Percentage of genes with cadherin and zinc finger annotations across interaction and loop anchor categories.

Barplots showing the percentage of genes annotated with cadherin (purple) or zinc finger (light blue) domains across (A) gene pair categories and (B) *E. scolopes* (C) *S. officinalis* and (D) *O. bimaculoides* loop anchors. Source data are provided as a Source Data file.

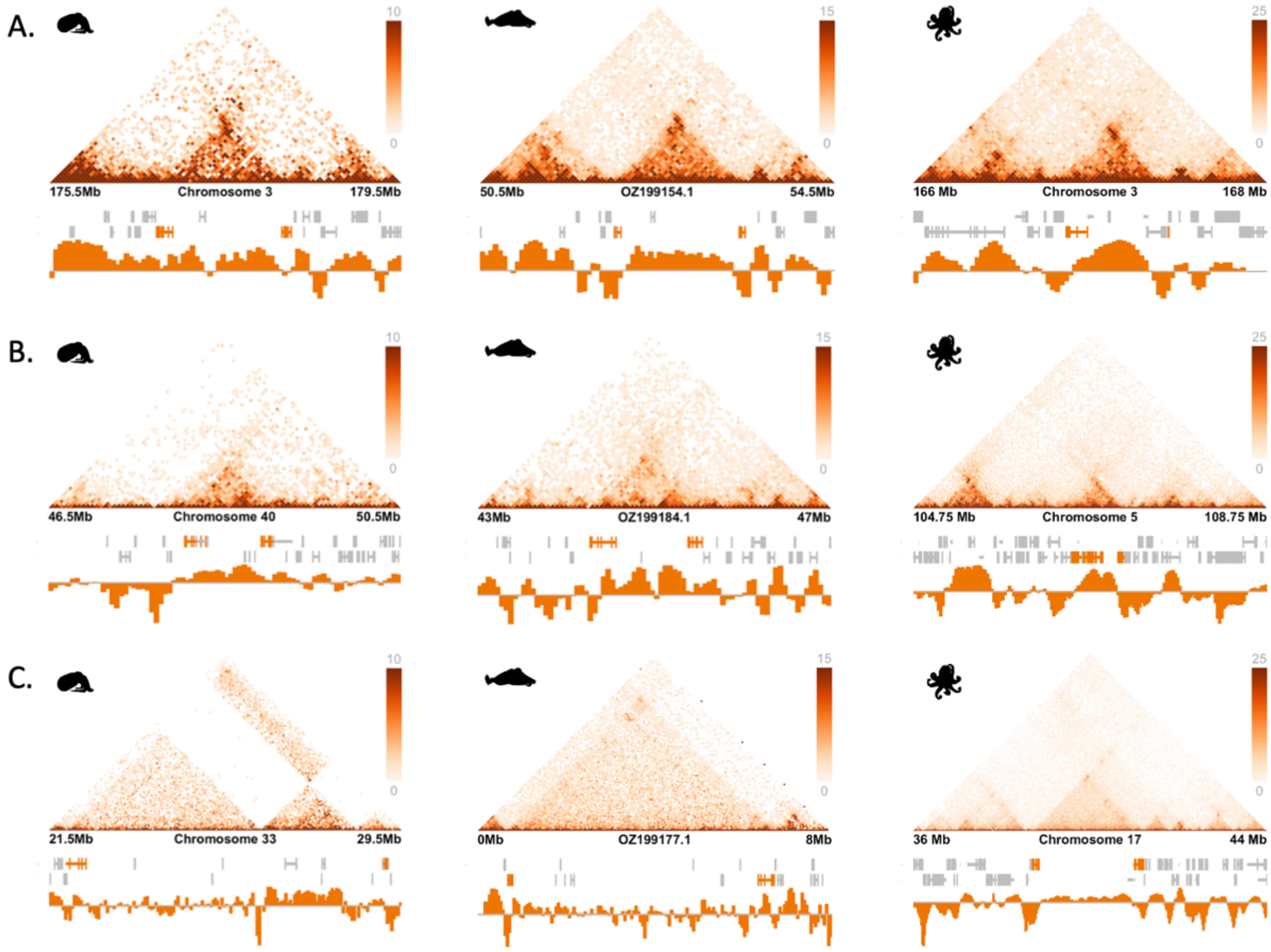

Figure S14. Loops conserved across *E. scolopes*, *S. officinalis*, and *O. bimaculoides*.
Micro-C contact matrices and insulation score profiles are shown for three conserved loops (A–C), each detected in all three species, identified at 100 kb and 50 kb resolution. Loops are shown at 50 kb resolution for *E. scolopes* (left) and *S. officinalis* (middle), and at 25 kb resolution *for O. bimaculoides* (right). Orange bars represent genes that overlap with loop anchors. Grey bars represent other genes in the depicted region. Orange tracks show insulation scores calculated using a 350 kb window. Loops were considered conserved if they contained at least one orthologous gene at both the start and end positions, regardless of gene orientation. Note that the *E. scolopes* region in panel C shows a localised signal dropout between loop anchors, likely reflecting a small misassembly. Nonetheless, orthologous genes are present at both ends, supporting conservation of the loop across species.

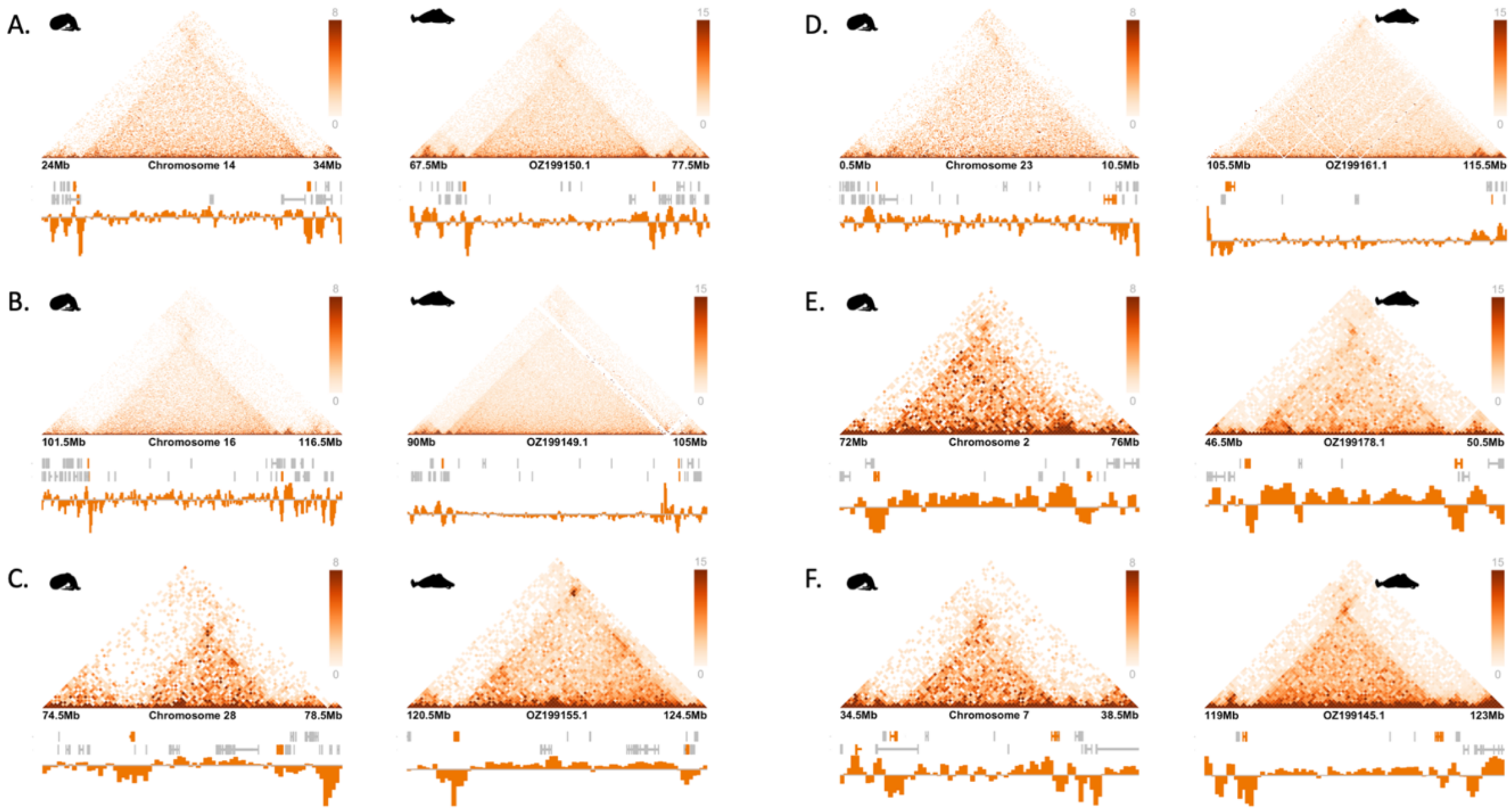

Figure S15. Examples of loops conserved between *E. scolopes* and *S. officinalis* with disrupted anchor organisation in *O. bimaculoides*.
Micro-C contact matrices and insulation score profiles are shown for six loops (A–F) conserved between *E. scolopes* (left) and *S. officinalis* (right), identified at 100 kb and 50 kb resolution. Loops are displayed at 50 kb resolution for both species. Orange bars indicate genes overlapping loop anchors; grey bars show other genes in the region. Orange tracks represent insulation scores calculated using a 350 kb window. Panels A–C show loops whose anchor orthologs are located on different chromosomes in *O. bimaculoides*. Panels D–F show loops whose anchor orthologs are located far apart on the same chromosome in *O. bimaculoides.*

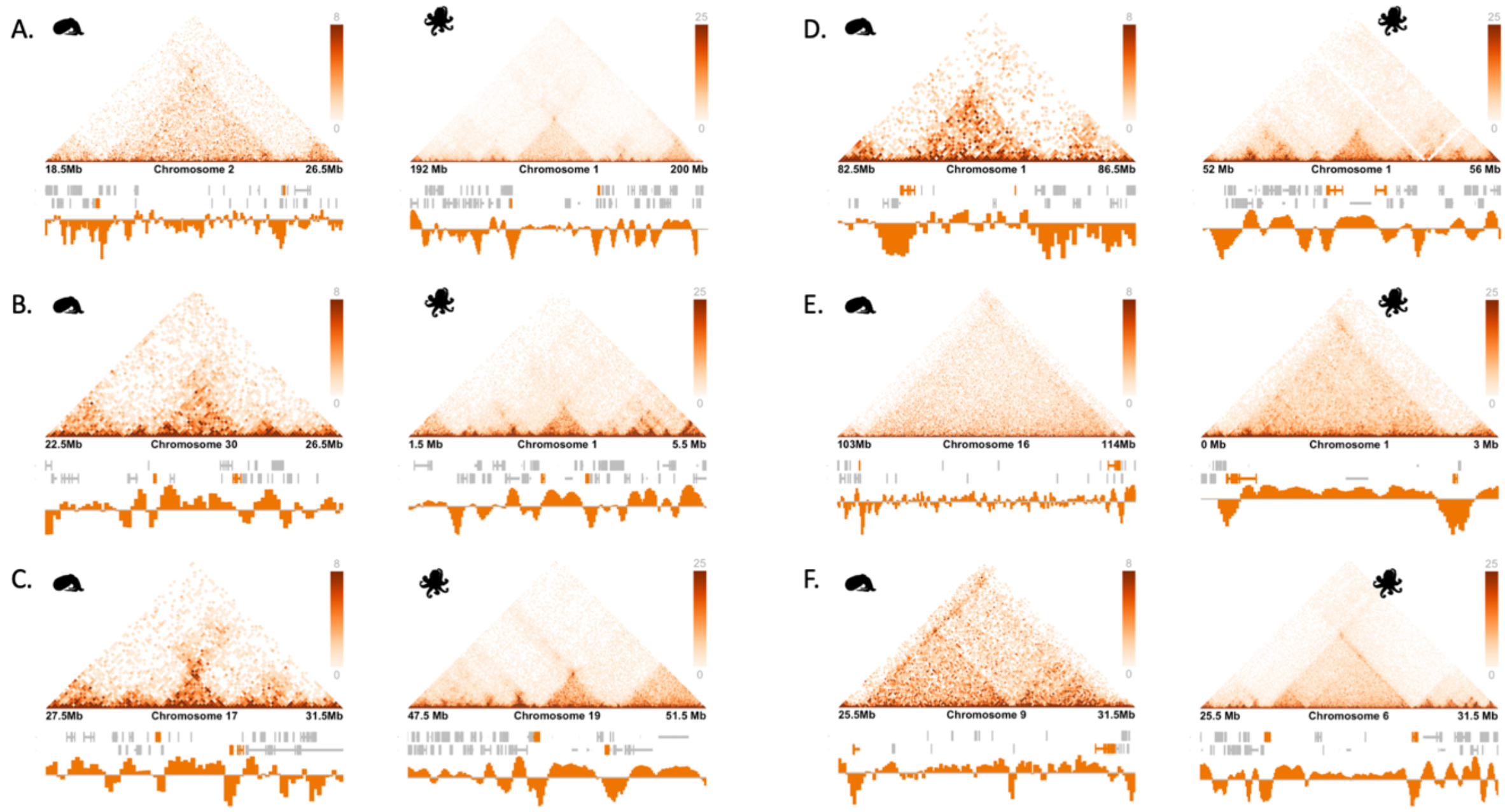

Figure S16. Loops conserved between *E. scolopes* and *O. bimaculoides*.
Micro-C contact matrices and insulation score profiles are shown for six loops (A–F) conserved between *E. scolopes* and *O. bimaculoides*, identified at 100 kb and 50 kb resolution. Loops are displayed at 50 kb resolution for *E. scolopes* and 25 kb resolution for *O. bimaculoides*. Orange bars indicate genes overlapping loop anchors; grey bars show other genes in the surrounding region. Insulation scores, calculated using a 350 kb window, are shown as orange tracks beneath each matrix. All conserved loops are shown in this figure, defined as those with at least one orthologous gene at both anchor positions, regardless of gene orientation.

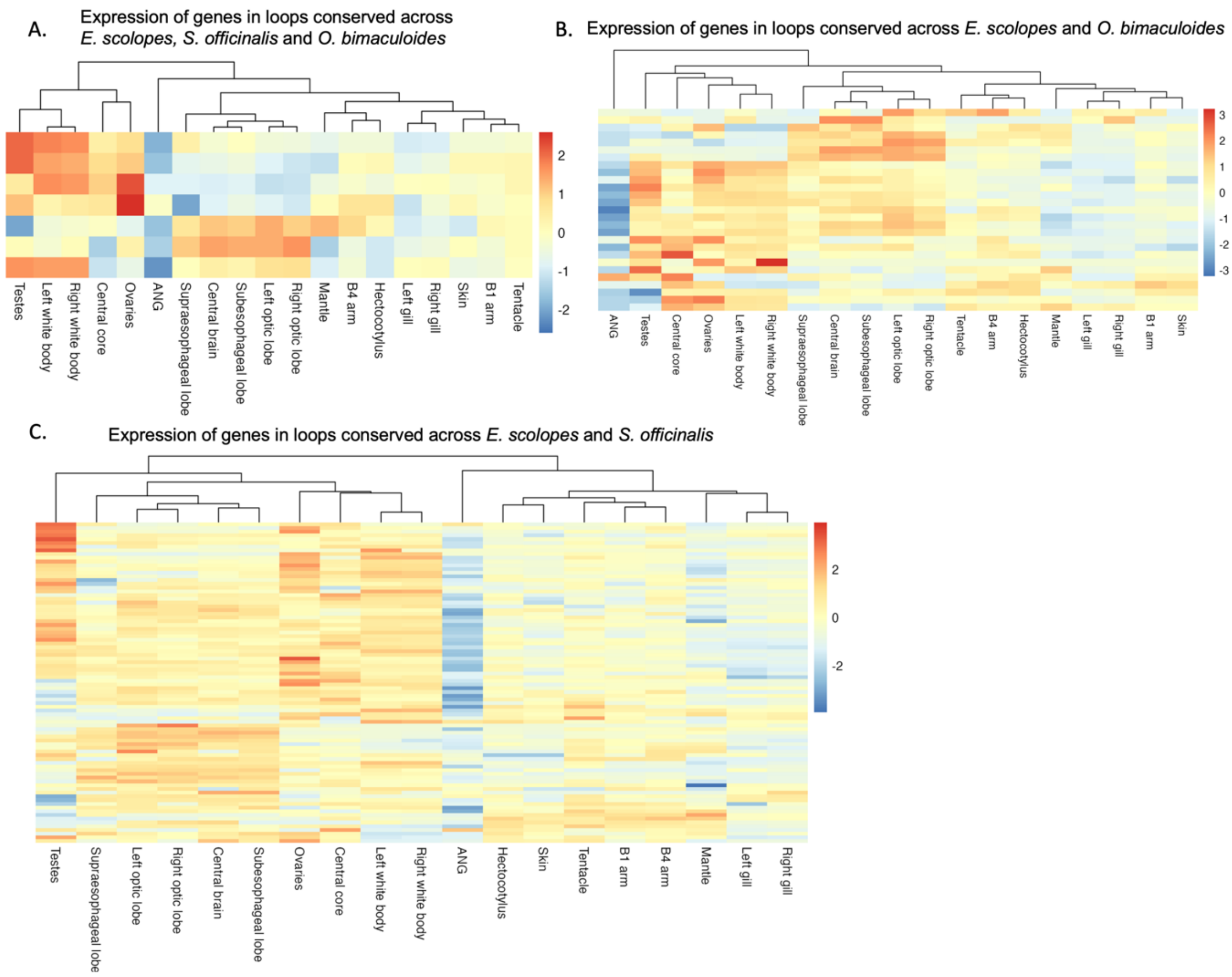

Figure S17. Expression of genes located in conserved loops across *E. scolopes* tissues.
Heatmaps showing log-transformed TPM expression of *E. scolopes* genes located in loops conserved across (A) *E. scolopes*, *S. officinalis*, and *O. bimaculoides*; (B) *E. scolopes* and *O. bimaculoides*; (C) *E. scolopes* and *S. officinalis*. Each row represents a gene and each column a tissue. Expression values are scaled by row (Z-score) to highlight tissue-specific expression patterns. Hierarchical clustering was applied to both genes and tissues using Euclidean distance and complete linkage. Source data are provided as a Source Data file.

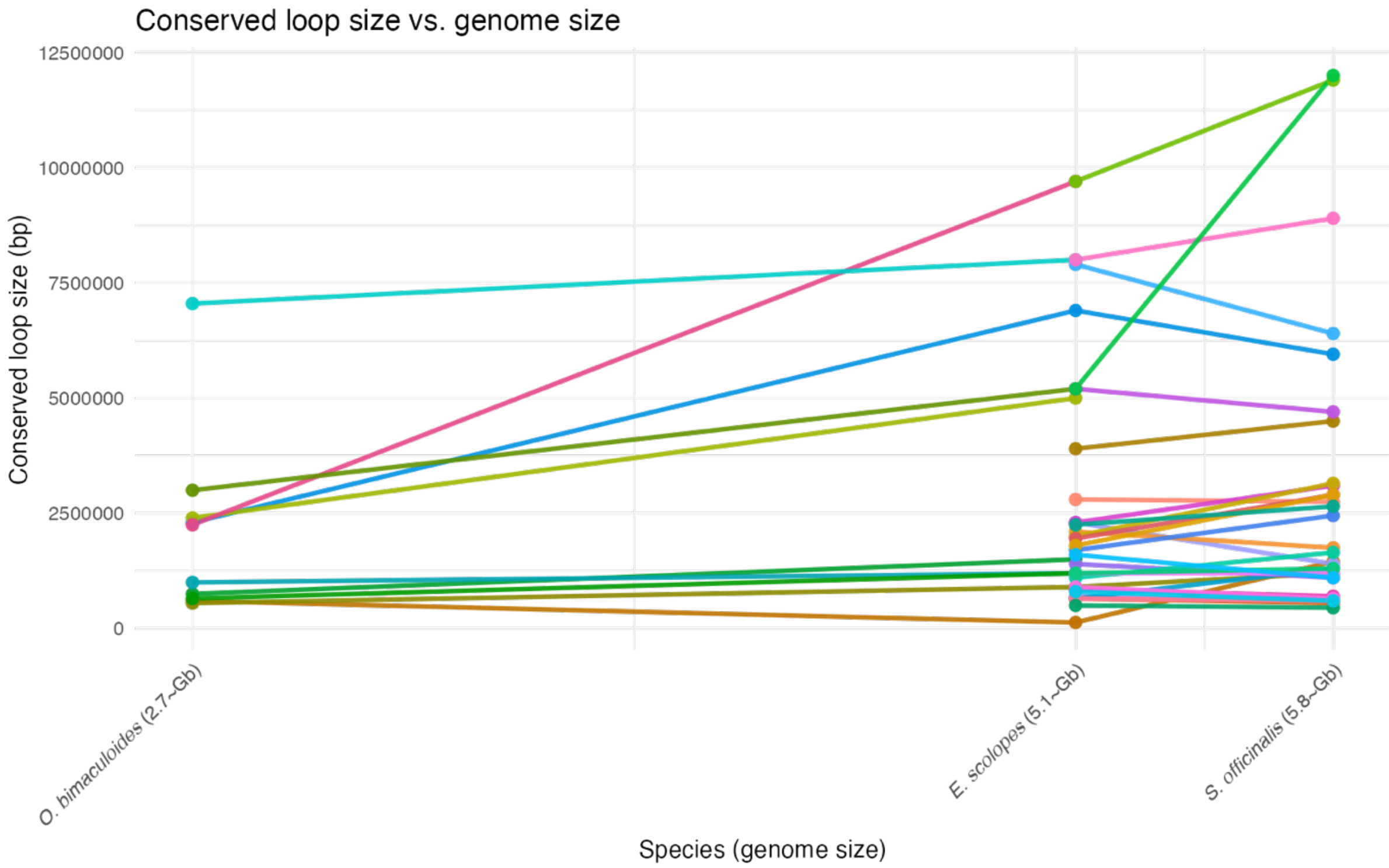

Figure S18. Relationship between conserved chromatin loop size and genome size across coleoid species. Each line and colour represents a conserved loop shared between two or more coleoid species, with loop size plotted on the y-axis and species (ordered by genome size) on the x-axis. Genome sizes are indicated in parentheses on the x-axis labels. Source data are provided as a Source Data file.

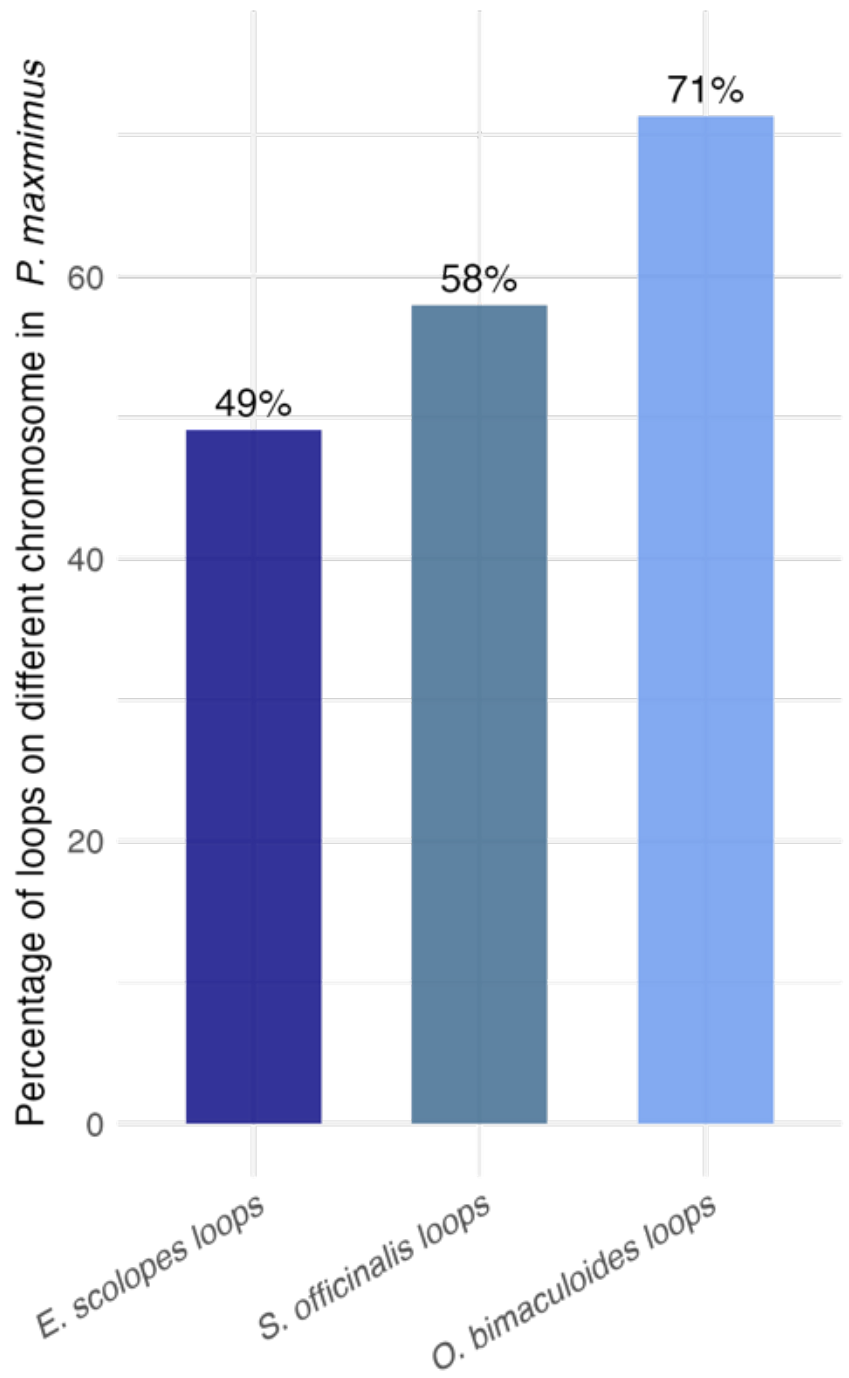

Figure S19. Percentage of loops in each coleoid species with orthologous genes located on more than one chromosome in *P. maximus*. Bar plot showing the proportion of chromatin loops in *E. scolopes*, *S. officinalis*, and *O. bimaculoides* in which the genes located at loop anchors map to different chromosomes in *P. maximus*. This serves as a proxy for the ancient coleoid chromosomal rearrangement event, with higher percentages indicating greater disruption of ancestral synteny. Source data are provided as a Source Data file.

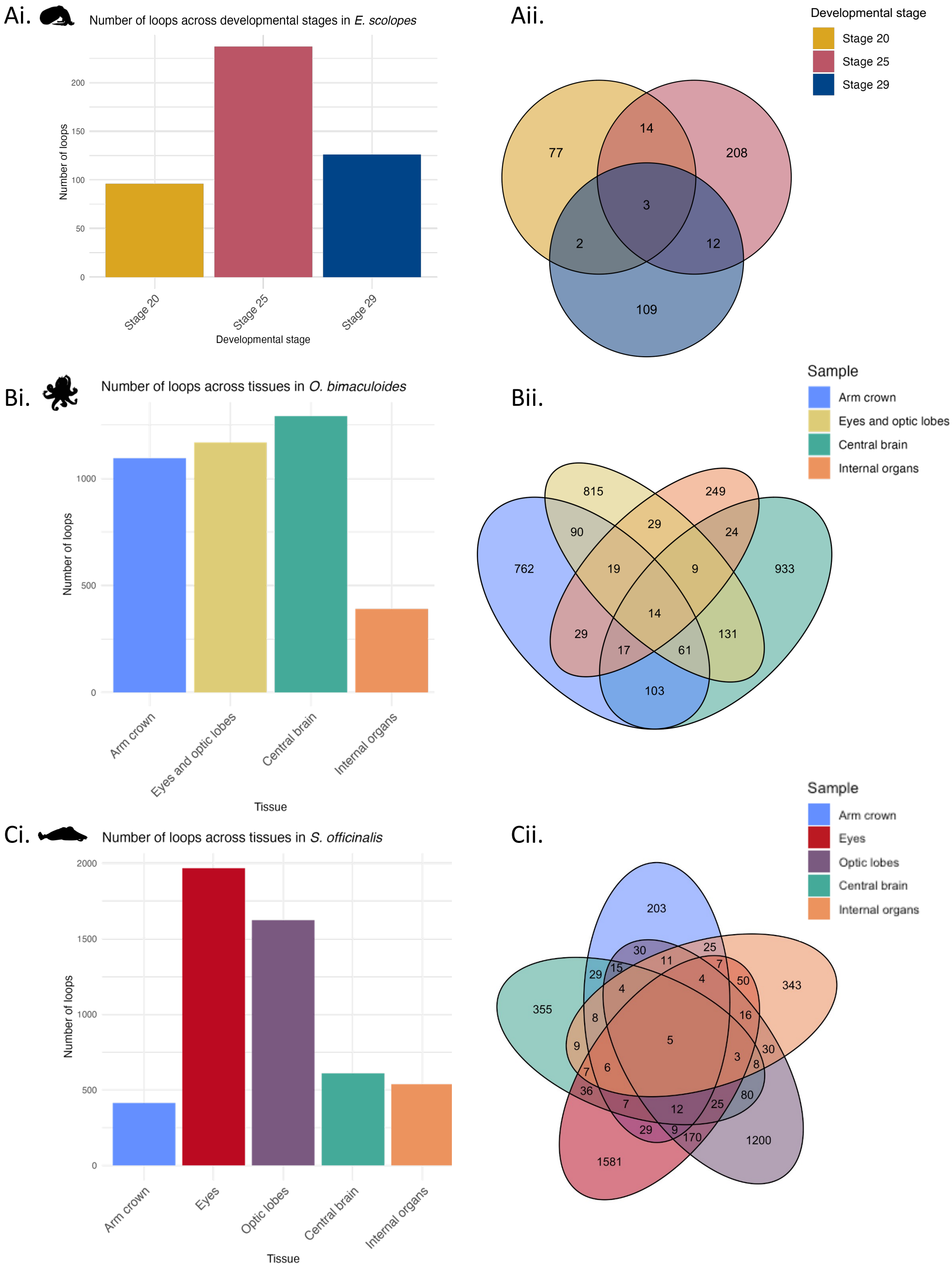

Figure S20. Number and overlap of chromatin loops across developmental stages and tissues in *E. scolopes*, *O. bimaculoides*, and *S. officinalis*.

(Ai) Number of chromatin loops identified in *E. scolopes* across three developmental stages and (Aii) Venn diagram showing the overlap of loops across these stages.

(Bi) Number of loops detected in four tissues in *O. bimaculoides* and (Bii) Venn diagram showing the overlap of loops across these tissues.

(Ci) Number of loops detected in five tissues in *S. officinalis* and (Cii) Venn diagram showing the overlap of loops across these tissues.

Loops were detected for each species at 100 kb and 50 kb resolution. Loops are shown here regardless of gene content at their anchors; i.e. loops may or may not contain genes at their anchor positions. Source data are provided as a Source Data file.

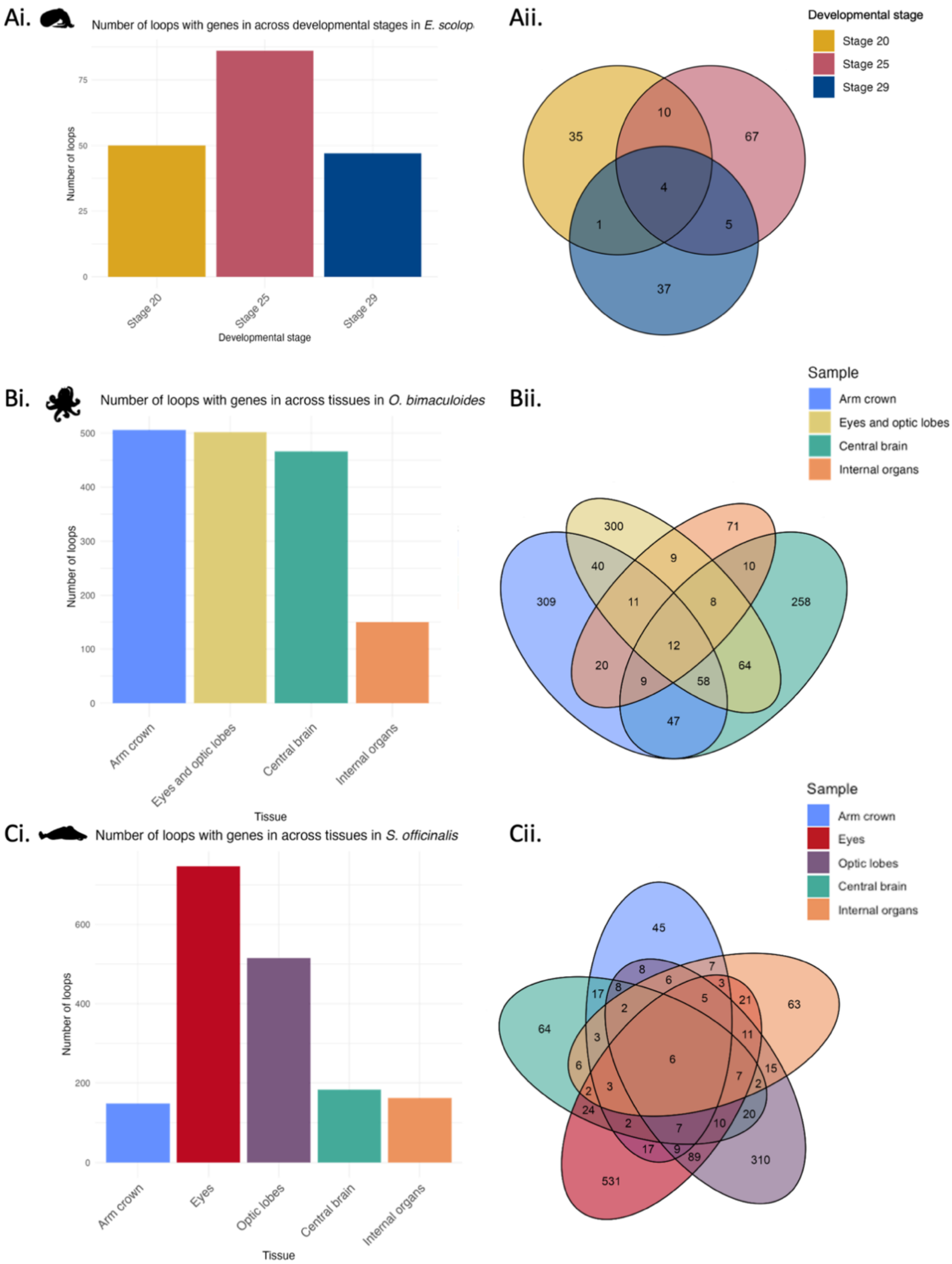

Figure S21. Number and overlap of loops containing genes at both anchors across developmental stages and tissues in *E. scolopes*, *O. bimaculoides*, and *S. officinalis*.
(Ai) Number of chromatin loops identified in *E. scolopes* across three developmental stages and (Aii) Venn diagram showing the overlap of loops across these stages.
(Bi) Number of loops detected in four tissues in *O. bimaculoides* and (Bii) Venn diagram showing the overlap of loops across these tissues.
(Ci) Number of loops detected in five tissues in *S. officinalis* and (Cii) Venn diagram showing the overlap of loops across these tissues.
Loops were detected for each species at 100 kb and 50 kb resolution. Only loops with at least one gene in both anchor regions were retained. Loops containing the exact same sets of genes at their anchors were considered duplicates and removed from the analysis. Source data are provided as a Source Data file.

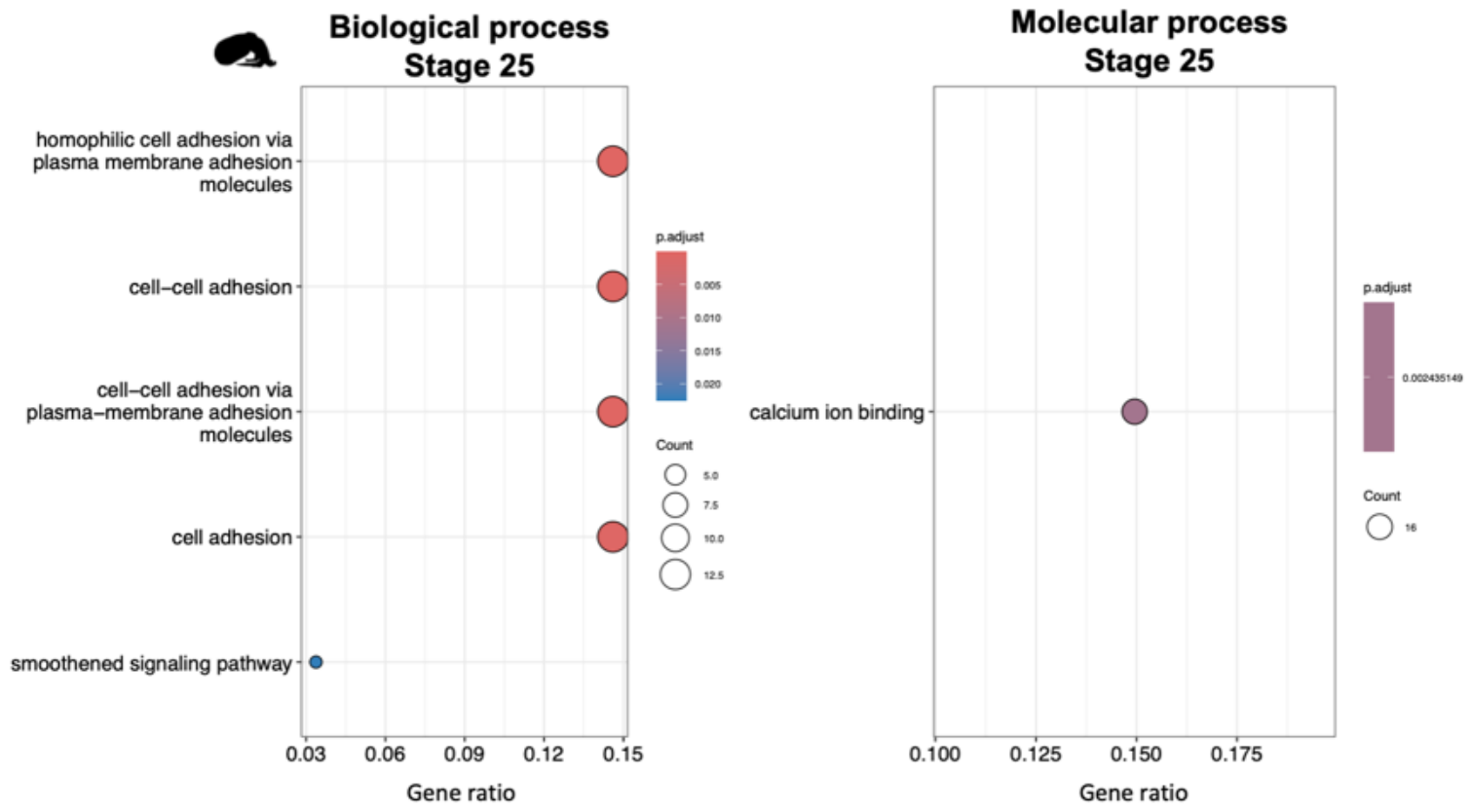

Figure S22. GO term enrichment results for genes located in loop anchors in developmental stage Micro-C data of *E. scolopes*. Grouped by GO category: biological process and molecular function. Each dot represents an enriched GO term, dot size indicates the number of genes associated with each term, and colour reflects statistical significance (adjusted P value). Gene ratio is defined as the number of genes in the query set annotated with a given term divided by the total number of query genes. Only genes located at loop anchors in loops detected at 100 kb and 50 kb resolution were included in the analysis. Source data are provided as a Source Data file.

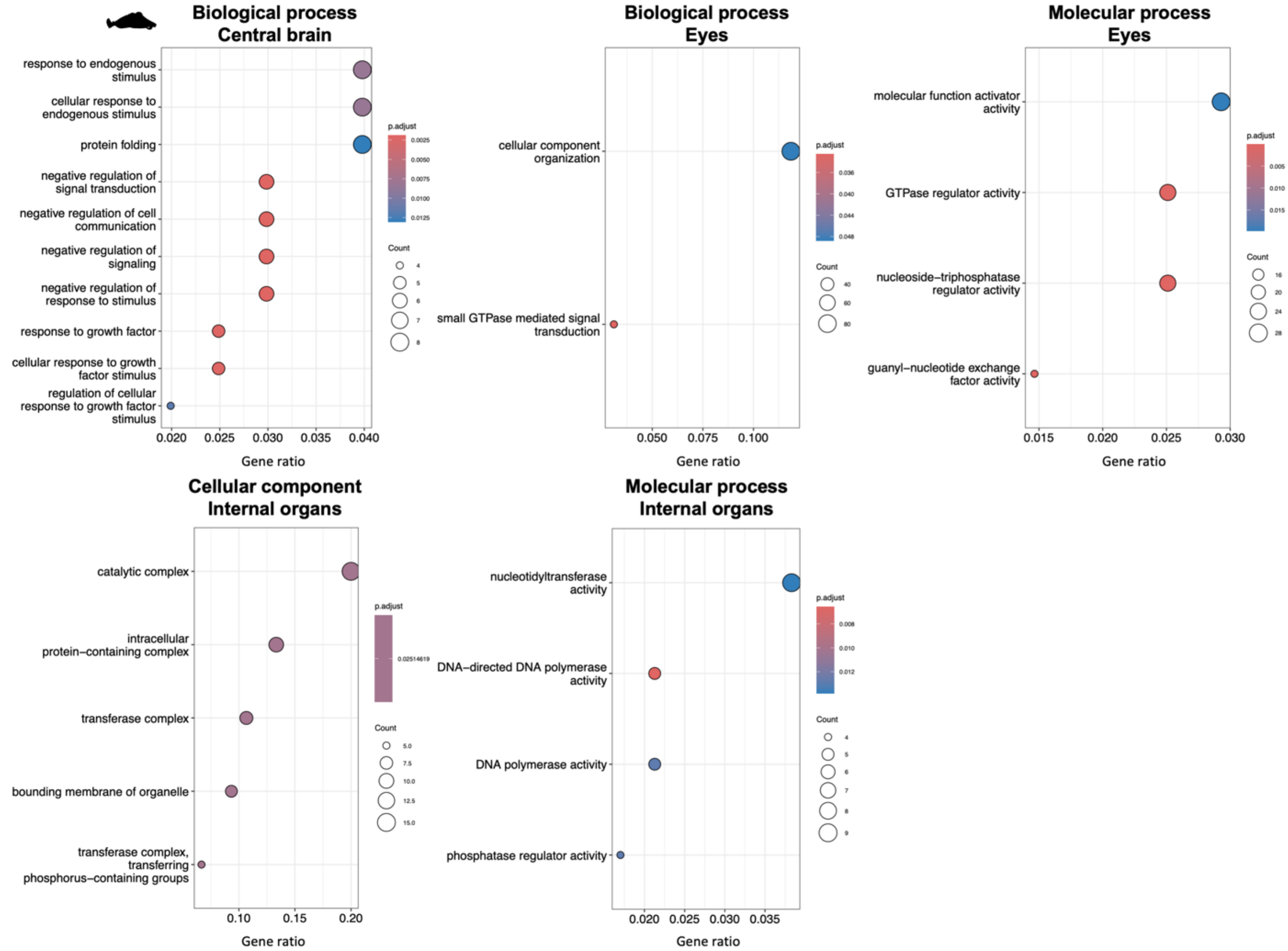

Figure S23. GO term enrichment results for genes located in loop anchors in tissue-specific data for *S. officinalis.* Grouped by GO category: biological process, cellular component, and molecular function. Each dot represents an enriched GO term, dot size indicates the number of genes associated with each term, and colour reflects statistical significance (adjusted P value). Gene ratio is defined as the number of genes in the query set annotated with a given term divided by the total number of query genes. Only genes located at loop anchors in loops detected at 100 kb and 50 kb resolution were included in the analysis. Source data are provided as a Source Data file.

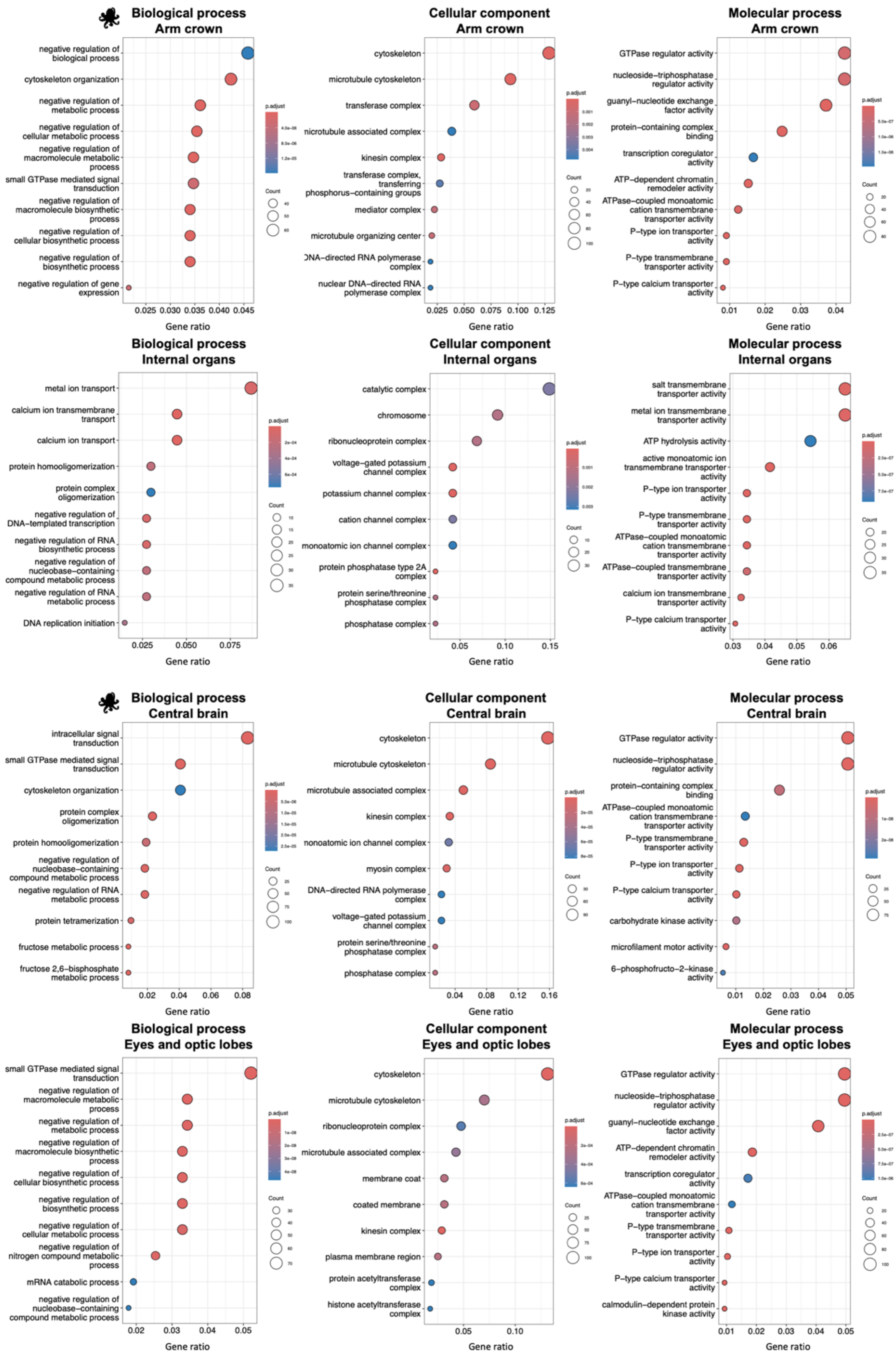

Figure S24. GO term enrichment results for genes located in loop anchors in tissue-specific data for *O. bimaculoides.* Grouped by GO category: biological process, cellular component, and molecular function. Each dot represents an enriched GO term, dot size indicates the number of genes associated with each term, and colour reflects statistical significance (adjusted P value). Gene ratio is defined as the number of genes in the query set annotated with a given term divided by the total number of query genes. Only genes located at loop anchors in loops detected at 100 kb and 50 kb resolution were included in the analysis. Source data are provided as a Source Data file.

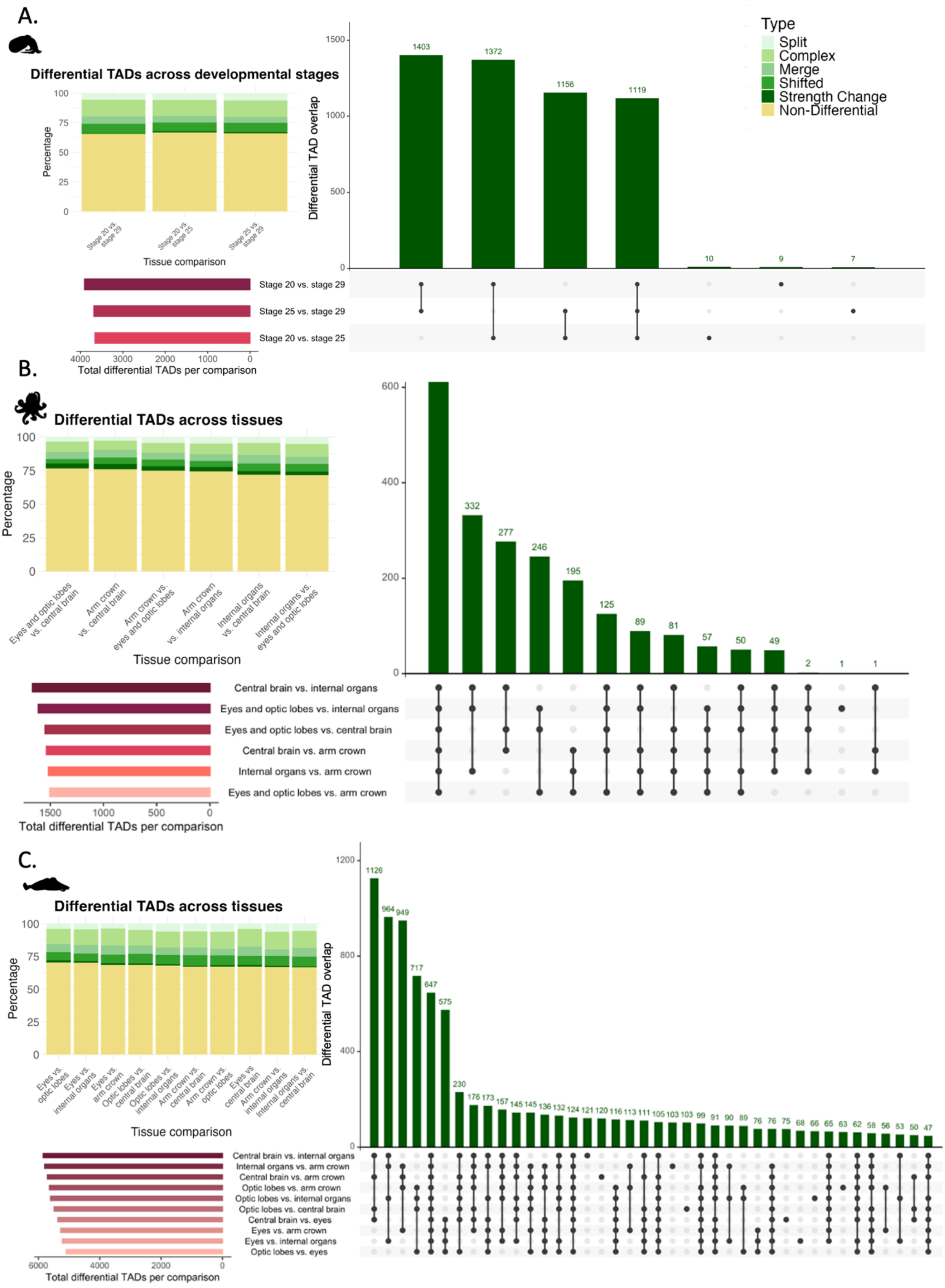
A.
Differential TADs across developmental stages
Percentage
Tissue comparison
Stage 20 vs. stage 29
Stage 20 vs. stage 25
Stage 25 vs. stage 29
Differential TAD overlap
1403
1372
1156
1119
10
9
7
Stage 20 vs. stage 29
Stage 25 vs. stage 29
Stage 20 vs. stage 25
Total differential TADs per comparison
Type
Split
Complex
Merge
Shifted
Strength Change
Non-Differential
B.
Differential TADs across tissues
Percentage
Eyes and optic lobes vs. central brain
Arm crown vs. central brain
Arm crown vs. eyes and optic lobes
Arm crown vs. internal organs
Internal organs vs. central brain
Internal organs vs. eyes and optic lobes
Tissue comparison
332
277
246
195
125
89
81
57
50
49
2
1
1
Central brain vs. internal organs
Eyes and optic lobes vs. internal organs
Eyes and optic lobes vs. central brain
Central brain vs. arm crown
Internal organs vs. arm crown
Eyes and optic lobes vs. arm crown
Total differential TADs per comparison
C.
Differential TADs across tissues
Percentage
Eyes vs. optic lobes
Eyes vs. internal organs
Eyes vs. arm crown
Optic lobes vs. central brain
Optic lobes vs. internal organs
Arm crown vs. central brain
Arm crown vs. optic lobes
Eyes vs. central brain
Arm crown vs. internal organs
Internal organs vs. central brain
Tissue comparison
Differential TAD overlap
1126
964
949
717
647
575
230
176
173
157
145
145
136
132
124
121
120
116
113
111
105
103
103
99
91
90
89
76
76
75
68
66
65
63
62
58
56
53
50
47
Central brain vs. internal organs
Internal organs vs. arm crown
Central brain vs. arm crown
Optic lobes vs. arm crown
Optic lobes vs. internal organs
Optic lobes vs. central brain
Central brain vs. eyes
Eyes vs. arm crown
Eyes vs. internal organs
Optic lobes vs. eyes
Total differential TADs per comparison

Figure S25. Differential TADs across developmental stages and tissues in *E. scolopes*, *O. bimaculoides*, and *S. officinalis*. (A) Differential TADs identified across developmental stages in *E. scolopes*, (B) across stage 20 tissues in *O. bimaculoides*, and (C) across stage 29 tissues in *S. officinalis*.
Top left: stacked barplots showing the proportion of differential TADs per comparison by type of change (split, merge, shifted, complex, strength change), with non-differential TADs shown in grey. Bottom left: total number of differential TADs detected per pairwise comparison. Right: UpSet plots showing shared and unique differential TADs across comparisons. Differential TADs were identified using TADCompare and analysed at 100 kb resolution for *E. scolopes* and *S. officinalis*, and 50 kb resolution for *O. bimaculoides*, using a 1 Mb sliding window. If a TAD contained an NA in the 'Differential' classification in any comparison, it was excluded from all comparisons. TADs were considered overlapping if they shared exact coordinates and were classified as differential in each file. TADs classified as differential in one comparison and non-differential in all others were counted as unique to that comparison. Source data are provided as a Source Data file.

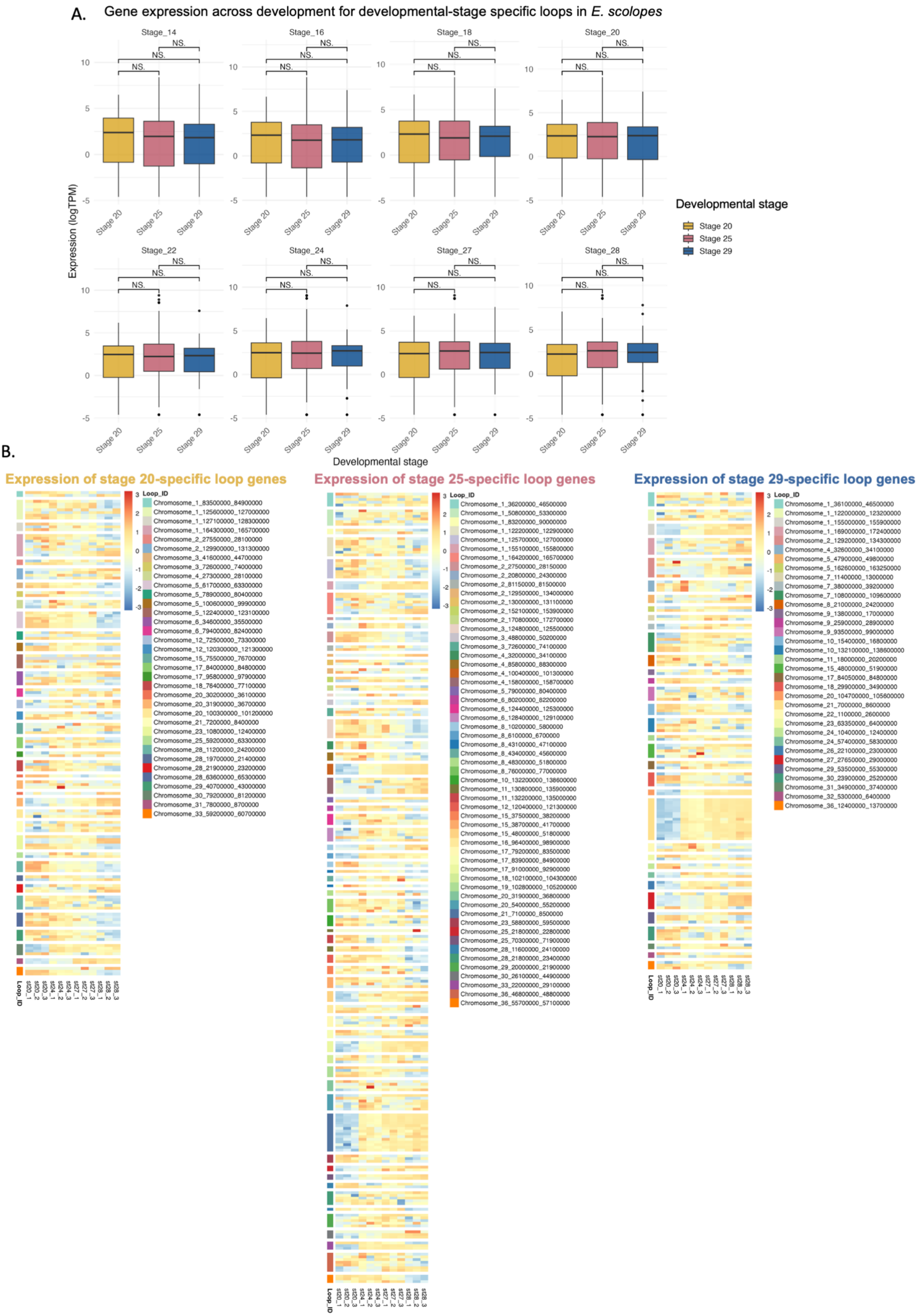
A.
Gene expression across development for developmental-stage specific loops in E. scolopes
Stage_14
Stage_16
Stage_18
Stage_20
Stage_22
Stage_24
Stage_27
Stage_28
NS.
Expression (logTPM)
Developmental stage
Stage 20
Stage 25
Stage 29
B.
Expression of stage 20-specific loop genes
Expression of stage 25-specific loop genes
Expression of stage 29-specific loop genes
Loop_ID

Figure S26. Gene expression patterns across development for *E. scolopes* genes located in developmental stage-specific loops.

(A) Boxplots showing log-transformed TPM expression across three developmental stages (stage 20, 25, and 29) for genes located in loops specific to each stage. Each panel shows the expression of stage-specific loop genes (indicated along the x-axis) across all three stages, highlighting changes in expression across development. Statistical comparisons were performed using two-sided Wilcoxon rank-sum tests; 'NS' indicates non-significant comparisons ($P > 0.05$). Sample sizes for each group are provided in the Source Data file. Centre line, median; box limits, upper and lower quartiles; whiskers, 1.5 × interquartile range; points represent outlying loop genes. Genes found in loops shared across multiple developmental stages were excluded to ensure stage specificity.

(B) Heatmaps showing log-transformed TPM expression of genes located in stage-specific loops across developmental stages: genes in stage 20-specific loops (left), stage 25-specific loops (middle), and stage 29-specific loops (right). Rows represent individual genes, and columns represent three biological replicates per developmental stage. Each heatmap is scaled by row to highlight stage-specific expression patterns. Genes with zero expression (TPM = 0) and no variation across all samples were excluded, and only loops with more than one remaining expressed gene were retained. As in panel A, loops with gene sets shared across multiple developmental stages were excluded.

Loops were detected at 100 kb and 50 kb resolution. Source data are provided as a Source Data file.

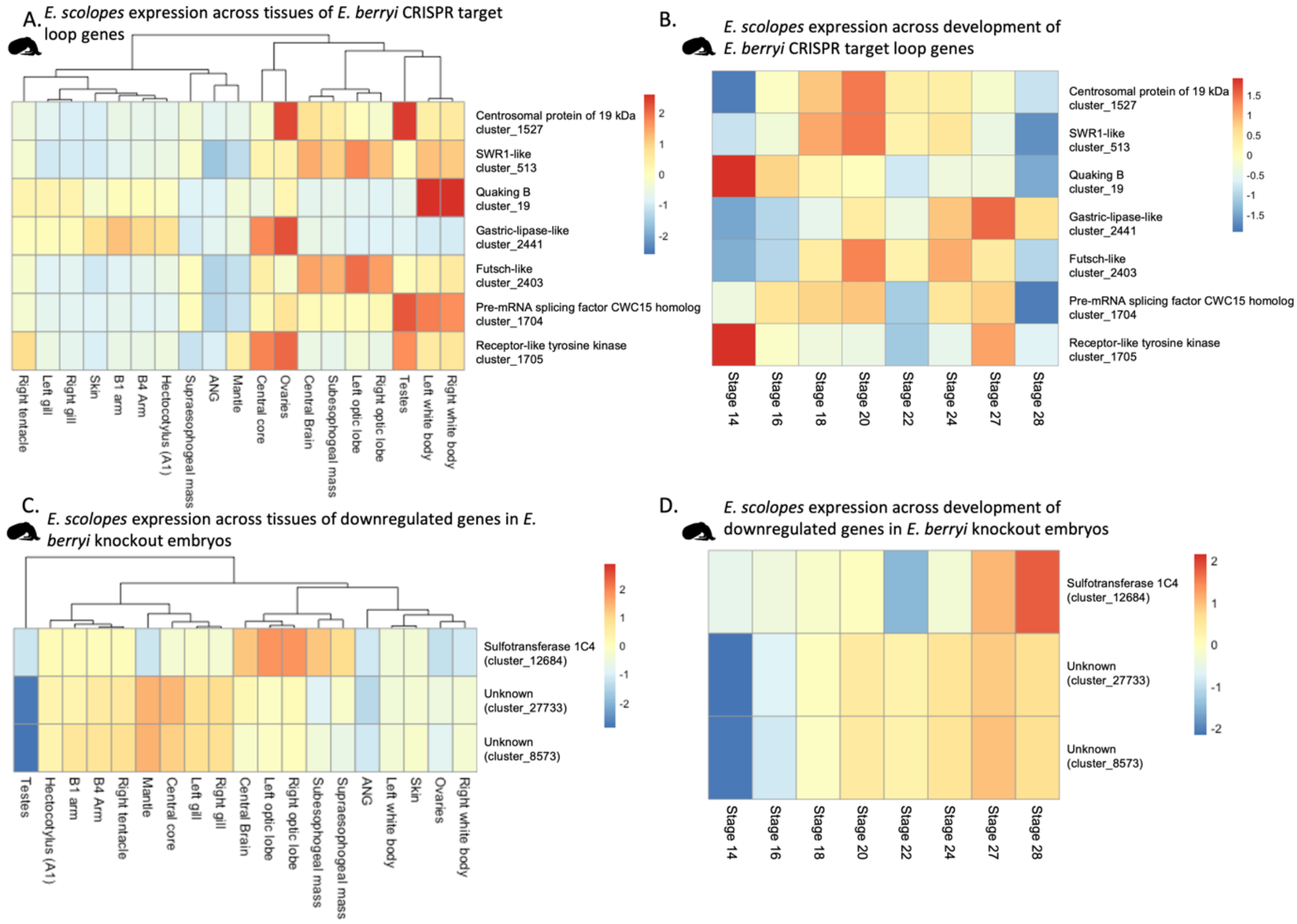

Figure S27. Expression of *E. scolopes* genes associated with CRISPR-targeted loops and transcriptional response in *E. berryi* knockout embryos.

(A) Expression across tissues in *E. scolopes* orthologs located in the loop targeted by CRISPR-Cas9 in *E. berryi*.

(B) Expression across development in *E. scolopes* orthologs located in the loop targeted by CRISPR-Cas9 in *E. berryi*.

(C) Expression across tissues in *E. scolopes* of orthologs of genes near target loop that were significantly downregulated (Wald test, BH correction) in *E. berryi* CRISPR knockout embryos.

(D) Expression across developmental stages of the same significantly downregulated (Wald test, BH correction) ortholog set in *E. scolopes*.

Heatmaps display log-transformed TPM values across tissues or developmental stages. Rows represent genes, columns represent biological samples averaged across replicates. Expression values are row-scaled to highlight expression patterns across conditions. Tissues were hierarchically clustered in panels (A) and (C), while developmental stages in panel (B) and (D) were shown in chronological order without clustering to preserve temporal progression. Note: One *E. berryi* gene (EB05908.1) is absent from panels C and D because it is unannotated in the *E. scolopes* gene annotation used in this study, despite having a clear BLAST hit in the region shown in Fig. 5E. This gene is also annotated as G5765 in the *E. scolopes* gene annotation from[2]. Source data are provided as a Source Data file.

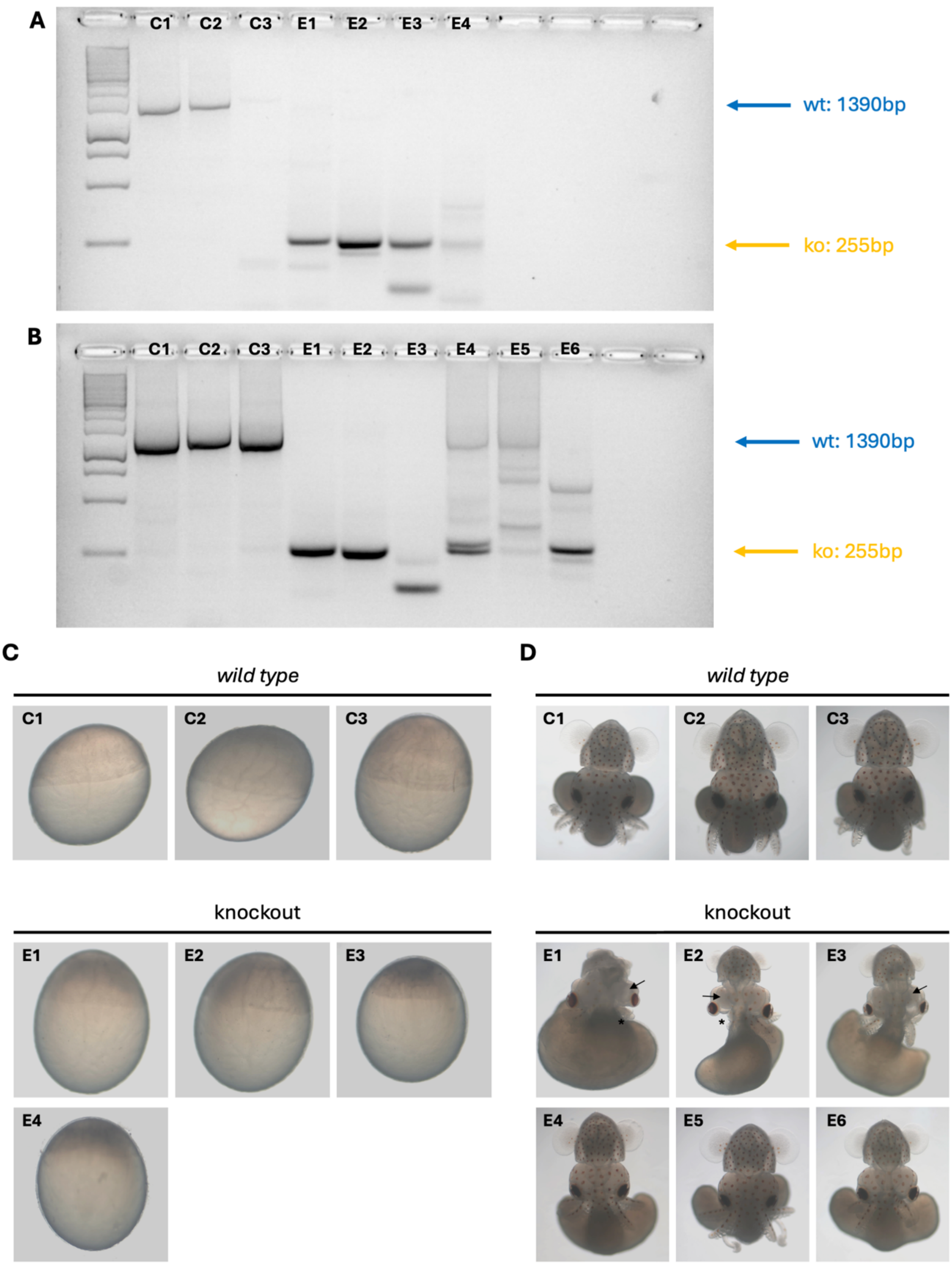

Figure S28. Genotypic and phenotypic analysis of crispant embryos.
(A) Agarose gel displaying results of genotyping PCR of embryos depicted in (C). Expected wild type band is 1390 bp (blue arrow) and expected knockout band is 255 bp (orange arrow).
(B) Agarose gel displaying results of genotyping PCR of embryos depicted in (D). Expected wild type band is 1390 bp (blue arrow) and expected knockout band is 255 bp (orange arrow).
(C) Wild type and knockout embryos that were used for RNA sequencing analysis at developmental stage 14.
(D) Wild type and knockout embryos that were used for RNA sequencing analysis at developmental stage 28. Embryos E1-E3 showed significant developmental phenotypes, including smaller optic lobes (arrow) and smaller or missing arms (star), while Embryos E4-E6 showed very slight (developmental delay) to no phenotype after gRNA injection.

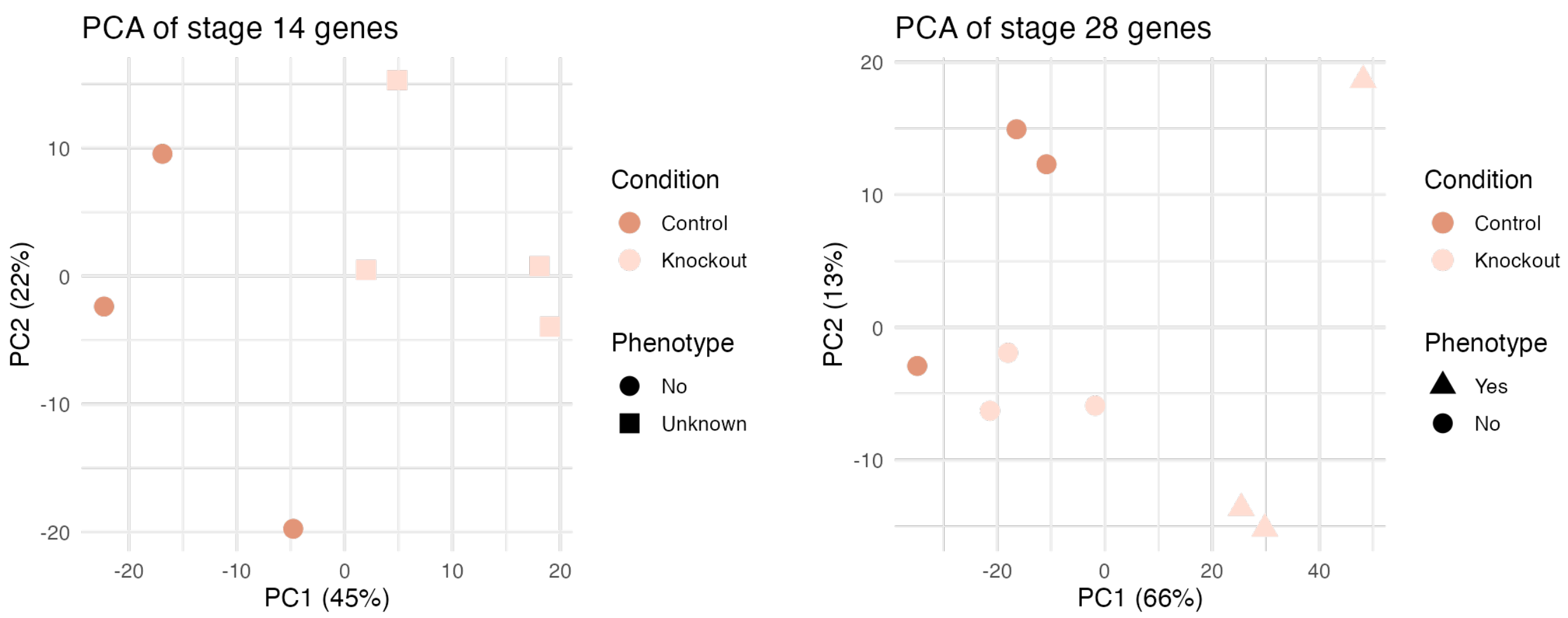

Figure S29. Principal component analysis (PCA) of *E. berryi* CRISPR embryos at stages 14 and 29. PCA plots showing transcriptome profiles of *E. scolopes* embryos injected with CRISPR-Cas9 constructs compared to uninjected controls. Analyses were performed separately for stage 14 (left) and stage 29 (right) embryos. Each point represents a single embryo, coloured by experimental condition (control or injected) and shaped by phenotypic outcome (present, absent, or unknown). PCA was performed on variance-stabilised (VST) RNA-seq expression values across all genes expressed at each developmental stage. Axes indicate the percentage of variance explained by each principal component. Source data are provided as a Source Data file.

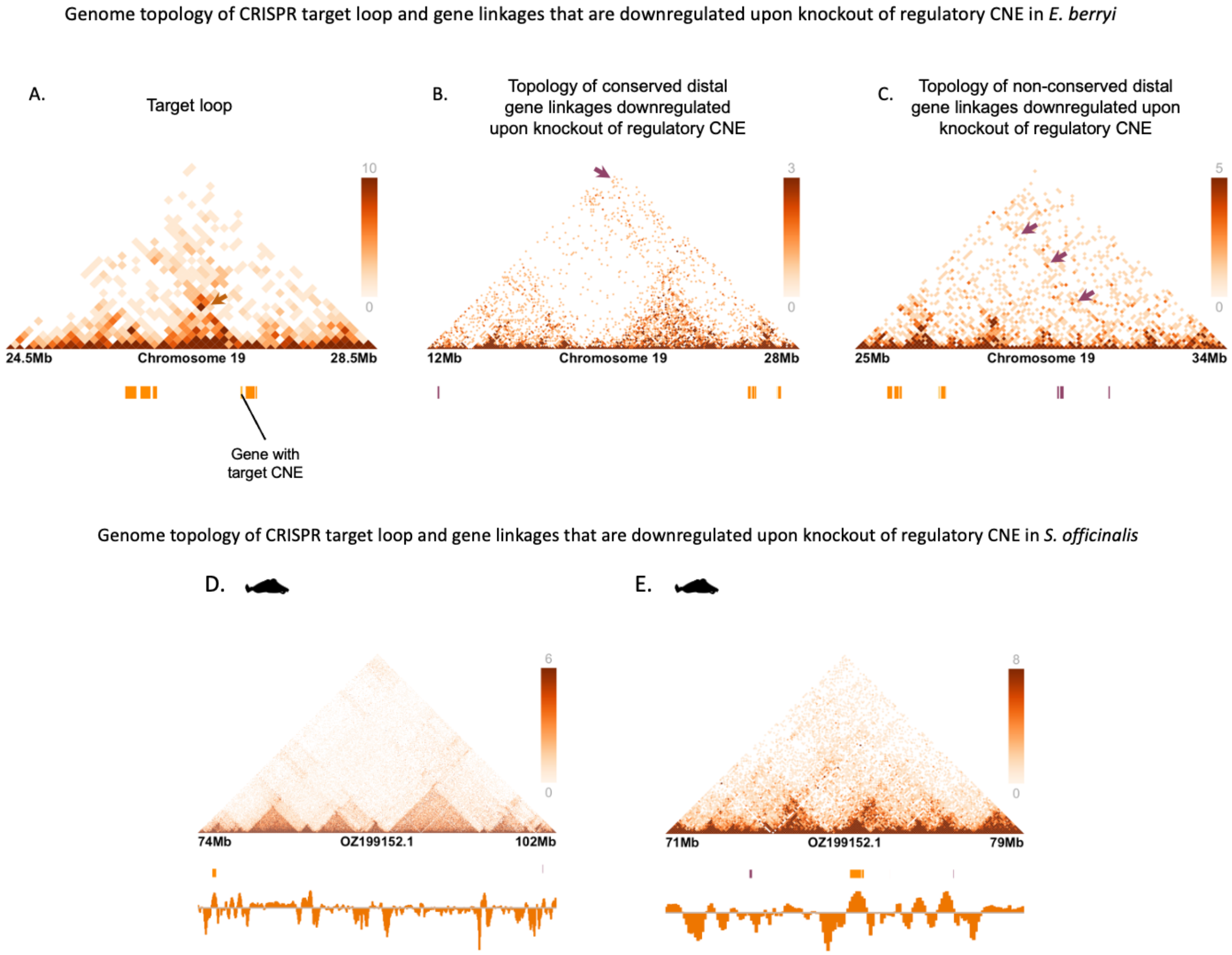

Figure S30. Genome topology of CRISPR target loop and distal downregulated gene linkages in *E. berryi* and *S. officinalis*.

(A) Micro-C contact map showing the CRISPR-targeted loop on chromosome 19 in *E. berryi*. The gene containing the targeted putative regulatory non-coding sequence is highlighted in yellow and marked below the map.

(B) *E. berryi* topology of distal gene linkages downregulated upon knockout of the target intron, which is conserved in *E. scolopes* and *O. bimaculoides* (orthologous to the contacts shown in Fig. 5D).

(C) Same as (B), but showing downregulated gene linkages in *E. berryi* that are not conserved in *O. bimaculoides* (but still orthologous to *E. scolopes*, as shown in Fig. 5E).

(D) Micro-C contact map of the corresponding CRISPR target loop in *S. officinalis*. Note that not all genes could be annotated in this species; only cluster_1527/EB05998, cluster_2403/EB06550, cluster_1704/EB06007, and cluster_1705/EB06081 were confidently mapped.

(E) *S. officinalis* topology of distal gene linkages orthologous to downregulated genes in *E. berryi*, conserved across *E. scolopes* and *O. bimaculoides* (as in Fig. 5D). Note that the loop appears only partially conserved in *S. officinalis* due to limitations in genome annotation: *E. scolopes* genes were mapped onto the genome and only top-ranked mRNA hits were retained to create the gene annotation, therefore, some genes are missing. Furthermore, the target gene Quaking B itself is located on an unplaced scaffold in *S. officinalis.*

Contact maps for *E. berryi* are shown at 100 kb resolution, and for *S. officinalis* at 50 kb resolution. Genes within the CRISPR target loop are highlighted in orange below the contact map, with the gene with the target intron shown in yellow. Genes that were downregulated in *E. berryi* knockout embryos are marked in purple. Background genes were removed from the contact map gene annotations to improve clarity.

Supplementary Notes

Supplementary Note 1. *Evolutionary braiding entropy reflects chromatin interaction conservation*

While fusion-with-mixing has been reported in the literature, measurements of mixing are not clearly established. As more genomes become available, we can begin to measure not just positional entropy (shuffling along a genomic coordinate), but also mixing of the homology assignments ('lines') between multiple genomes or species. For this, we applied concepts from topological theory to investigate the degree and entropy of braided structures (i.e., mixing of homologous coding and non-coding element order, irrespective of the exact base pair position) on chromosomes[3]. For the example shown in Fig. 2C, the homologous region between *S. officinalis* and *E. scolopes* (the Decapodiformes) showed a braiding entropy of < 0.01 (indicating almost no mixing), whereas between the more distantly related species *E. scolopes* and *O. bimaculoides* (Coleoidea), high entropy of 0.96 was detected. We quantified the degree of mixing for three different sizes of homologous regions (1 Mb, 10 Mb, and 50 Mb) and found the smallest window had very little mixing signal difference (median entropy of < 0.001 for all species combinations). The largest difference in mixing between these two phylogenetic nodes was observed for 10 Mb windows and comprised an entropy of 0.6 between *E. scolopes* and *O. bimaculoides*, compared to < 0.001 between *E. scolopes* and *S. officinalis*. The time of coleoid cephalopod separation was thus sufficient to achieve a relatively high mixed state, whereas decapodiform species showed almost no entropy increase, despite multiple apparent inversions on Oxford dotplots (Fig. 2A). For larger regions, such as 50 Mb, we observed higher entropy of mixing: 1.53 between *E. scolopes* and *O. bimaculoides* and 0.84 between *E. scolopes* and *S. officinali*s. Together, this suggests that within Decapodiformes the positioning of coding and non-coding elements is not as highly mixed or entropic, especially for smaller windows, whereas between Decapodiformes and Octopodiformes, the states are highly mixed.

To test the degree of mixing within homologous chromosomes or sub-chromosomal regions with respect to their chromatin organisation, we partitioned the mixing results above into categories of regions that span gene pairs from different interaction categories, according to their Micro-C strength: (1) interacting across coleoids, (2) *O. bimaculoides*-only interactions, (3) decapodiform-only interactions, or (4) gene pairs not in conserved coleoid interactions. Between *E. scolopes* and *O. bimaculoides*, the median degree of braid entropy for 10 Mb homologous regions that contain at least one gene pair interacting across the coleoids was 0.609, for regions with decapodiform-only interactions 0.529, for regions with octopodiform-only interactions 0.429, compared to 0.306 for 10 Mb regions that had no conserved coleoid interaction (two-sided Wilcoxon rank-sum test, P = 0.054). The braiding entropy scaled with the homology window size, with smaller windows having less entropy than larger ones. For 1 Mb homologous windows, windows with interactions conserved across the coleoids showed

entropy of 0.001, 0 for decapodiform-only, 0.001 for octopus-only, and 0 for windows with no conserved interactions. For 50 Mb homologous windows, the entropy was 1.283, 1.426, and 0.985 for windows with conserved coleoid interactions, decapodiform-only interactions, and octopodiform-only interactions, respectively. No 50 Mb windows had exclusively 'gene pairs not in conserved coleoid interactions'. This indicates that genomic topological linkages as measured by conserved interactions facilitate stronger mixing within the homologous regions. This complements the notion of frequent syntenic breaks at topological domain boundaries[4,5], with the implication that mixing is stronger within chromatin domains. This further suggests that a minimal chromatin domain size (i.e. >1 Mb) is required for mixing to manifest at the evolutionary scale.

Supplementary Note 2. *Interaction conservation and ancestral genome organisation shape tissue-wide gene expression and reflect regulatory constraints*

To investigate whether chromatin interactions influence gene expression, we analysed TPM-normalised expression levels of genes across *E. scolopes* tissues and interaction categories (Fig. S3, Supplementary Data 3). For this analysis, all genes in the 'interacting across the coleoids', 'interacting Decapodiformes only', 'interacting *O. bimaculoides* only' were retained, while genes in the 'not in conserved coleoid interactions' category were included only if they were exclusive to this category and not present in any interacting categories, preventing overlaps and ensuring clear comparisons.

Within each interaction category, variation in gene expression in *E. scolopes* was substantial, with broad interquartile ranges indicating tissue-specific differences in expression levels. However, across interaction categories, gene expression distributions were largely similar across tissues (Fig. S3B, C). Genes interacting in all species showed the highest median expression across *E. scolopes* tissues, followed by genes interacting in *E. scolopes* only, then genes interacting in *O. bimaculoides* only, with genes not in conserved coleoid interactions exhibiting the lowest median expression. These patterns suggest that genes in conserved interactions may be located in transcriptionally active regions that promote consistent and higher expression levels across tissues. Notably, genes interacting in all species had significantly higher expression (BH-adjusted two-sided Wilcoxon rank-sum test) in several tissues with coleoid innovations (Supplementary Data 3) such as the subesophageal lobe, supraesophageal lobe, central brain, central core, optic lobes, hectocotylus and accessory nidamental gland (ANG). Similarly, genes interacting in Decapodiformes only also had significantly higher expression compared to genes not in conserved coleoid interactions in some of these coleoid-specific tissues, including the subesophageal lobe, supraesophageal lobe, central brain, central core, optic lobes, hectocotylus, and ANG. Genes interacting only in *O. bimaculoides* exhibited significantly higher expression than genes not in conserved coleoid interactions in a few tissues, including the central core, left optic lobe, right optic lobe, and ANG. These results suggest that conserved chromatin interactions may contribute to the evolution of complex regulatory networks underpinning coleoid-specific adaptations.

To assess the tissue specificity of *E. scolopes* gene expression across interaction categories, we calculated the tissue specificity index (τ). Genes in the 'interacting in all species' category had the lowest τ values (median τ = 0.551), followed by those 'interacting in Decapodiformes only' (median τ = 0.580), indicating broader expression across tissues (Supplementary Data 4Ai). In contrast, genes interacting only in *O. bimaculoides* (τ = 0.606) and genes not in conserved coleoid interactions (median τ = 0.665) exhibited significantly higher tissue specificity (BH-adjusted two-sided Wilcoxon rank-sum test) (Supplementary Data 4Aii). Statistical comparisons confirmed that genes interacting in all species had significantly lower τ values than those in the 'interacting in *O. bimaculoides* only' (P = 0.014) and 'genes not in conserved coleoid interactions' (P = 0.0004) categories. However, differences between 'interacting in Decapodiformes only' and 'interacting in all species' were not significant, suggesting that conservation of interactions within Decapodiformes may also be linked to broader expression patterns.

These results are consistent with recent large-scale comparative transcriptomic studies showing that genes with broad expression patterns across tissues are under stronger evolutionary constraint, while more tissue-specific genes are less conserved at both the sequence and regulatory levels[6,7]. Broadly expressed genes, a proxy for pleiotropy, are less likely to undergo rapid changes in expression or sequence, likely due to their roles in multiple tissues and biological processes[6,7] Furthermore, highly expressed genes are likewise associated with stronger purifying selection, leading to slower evolutionary rates, likely due to the higher cost of transcriptional and translational errors[8,9]. Collectively, these findings support the idea that the most conserved interacting gene pairs are subject to particularly strong selective constraints, likely due to their broad expression and pleiotropic function.

To assess whether ancestral genomic organisation influences gene expression in *E. scolopes*, we compared co-expression patterns between genes whose orthologs were located on the same or different *P. maximus* chromosomes. Gene pairs interacting across the coleoids and located on the same *P. maximus* chromosome showed significantly higher *E. scolopes* gene expression correlation than those on different chromosomes (BH-adjusted two-sided Wilcoxon rank-sum test, P = 0.0094), suggesting that shared chromosomal context may contribute to regulatory coordination and constraint. A similar trend was observed for gene pairs interacting only in Decapodiformes, though this difference was marginally non-significant after correction (BH-adjusted P = 0.0574). In contrast, there was no significant difference (P > 0.05) in co-expression for gene pairs not in conserved coleoid interactions or those interacting only in *O. bimaculoides* (i.e not interacting in the species with gene expression data, *E. scolopes*).

Furthermore, when we compared expression levels across tissues (Fig. S3D-F), we found that for genes in gene pairs interacting in all species, those located on the same *P. maximus* chromosome exhibited higher median expression than those on different chromosomes (Fig. S3D), with significant differences in a range of tissues, some of which could be classed as

coleoid novelties, such as the ANG, B1 arm, B4 arm, central brain, central core, hectocotylus, optic lobes, right tentacle, right white body and skin (Supplementary Data 6). There were no significant differences in expression level between genes not in conserved coleoid interactions with different chromosomal origins across tissues. These results suggest that interacting gene pairs that have remained syntenic since the ancestral molluscan genome may promote higher expression and be under stronger regulatory constraints due to their transcriptional importance than genes from different *P. maximus* chromosomes, whereas genes not in conserved coleoid interactions are under similar relaxed constraints, regardless of chromosomal origin. On the other hand, tissue specificity (τ) did not differ significantly between same and different chromosomal origin across any of the four interaction categories, suggesting that ancestral chromosomal context is not a major determinant of expression breadth. Together, these findings indicate that while ancestral synteny correlates with higher expression and co-expression in conserved interacting pairs, interaction status more strongly reflects underlying pleiotropic constraints and evolutionary pressures.

Hierarchical clustering of gene expression patterns were very similar (Fig. S3C, F), with no strong separation based on interaction status or chromosomal origin. While some variation in expression levels is evident from the heatmaps, genes across all groups exhibited broadly comparable expression distributions. This suggests that while conserved interactions may contribute to overall expression levels (as indicated by the boxplots), they do not necessarily drive distinct expression patterns across tissues.

Overall, these findings suggest that conserved chromatin interactions are associated with higher, more coordinated, and more broadly expressed gene expression patterns across tissues compared to genes not in conserved coleoid interactions. This indicates that conserved coleoid interacting genes may maintain essential functions across multiple tissues[10]. In contrast, genes not in conserved coleoid interactions may have more specialised roles and/or evolve under relaxed regulatory constraints. Furthermore, chromosomal origin within *P. maximus* significantly influences gene expression levels and co-expression but does not affect tissue specificity, highlighting the contribution of large-scale genome reorganisation to the evolution of distinct gene regulatory programmes in coleoids.

Supplementary Note 3. *Functional enrichment patterns reflect interaction conservation and ancestral chromosomal context of gene pairs*

To investigate potential functional differences between gene interaction categories, we performed GO enrichment analyses across biological processes, molecular functions, and cellular components for genes in each interaction group (Fig. S4). As with the expression analyses (Fig. S

3A), all genes in the 'interacting across the coleoids', 'interacting Decapodiformes only', 'interacting *O. bimaculoides* only' were retained, while genes in the 'not in conserved coleoid interactions' category were included only if they were exclusive to this category. Genes

interacting across the coleoids were strongly enriched for core cellular and biosynthetic processes, particularly RNA processing and translation, with consistent enrichment for ribosome- and nuclear complex-associated functions across cellular component and molecular function categories, indicating a functionally central, housekeeping gene set, consistent with the results of the gene expression analyses.

Genes interacting in Decapodiformes only were enriched for protein catabolic processes, including ubiquitin-dependent proteolysis and proteasome-associated functions, suggesting a potential role in lineage-specific regulation of protein degradation and turnover. In contrast, genes interacting only in *O. bimaculoides* showed broader enrichment across both catabolic and biosynthetic processes, including protein folding, translation, and peptide metabolic processes, as well as peptidase activity and RNA polymerase-associated complexes. This may reflect species-specific adaptation or regulatory rewiring.

Genes that were not in conserved coleoid interactions were notably enriched for neural signalling-related functions, including neurotransmitter receptor activity, ligand-gated ion channel activity, and components of the synaptic membrane and postsynaptic compartments. These genes were also associated with DNA-binding transcription factor (TF) activity, suggesting a regulatory role possibly decoupled from deeply conserved chromatin architecture.

When comparing genes interacting in all species based on their ancestral chromosomal origin, we observed differences in enriched GO terms (Fig. S8). Genes interacting across the coleoids whose orthologs were located on the same *P. maximus* chromosome were enriched for core cellular and housekeeping functions, such as RNA processing and protein maturation, alongside catalytic and nuclear complex-associated activities. In contrast, genes interacting across the coleoids but located on different *P. maximus* chromosomes showed broader enrichment across both biosynthetic and catabolic processes, including translation, peptide metabolism, and protein degradation, as well as RNA-associated functions. These results suggest that even within highly conserved interaction groups, ancestral chromosomal origin is associated with functional coherence, with genes in ancestral interactions more tightly linked to core cellular processes, whereas those associated with rearranged genomic contexts show more functionally diverse profiles. For genes not in conserved coleoid interactions, GO results were more similar when comparing those with same versus different *P. maximus* chromosome origin (Fig. S8). Taken together, these results suggest that ancestral chromosomal proximity plays a role in maintaining the regulatory and functional coherence of conserved interactions between gene pairs, whereas genes not in conserved coleoid interactions may evolve under fewer structural constraints and show more functional heterogeneity.

To complement the GO analysis, we also assessed the enrichment of specific protein domain annotations associated with cephalopod nervous system development[11] across gene pair

categories. No significant enrichment of zinc finger or cadherin genes was detected in any gene pair group (Fig. S13), suggesting that these domain families are not strongly associated with conserved chromatin interaction status. Instead, their distribution may reflect lineage-specific expansions more closely tied to species-specific or novel aspects of genome architecture, rather than ancient, conserved regulatory interactions and core biological functions.

Supplementary Note 4. *Loop-associated genes are enriched for dynamic regulatory functions and transcription binding factor motifs across species and contexts*

To assess whether genes located at chromatin loop anchors are associated with particular functional roles, we performed GO enrichment analyses across all species, developmental stages and tissues (Fig. S12, S22, S23, S24). Despite differences in loop distribution and usage across categories, a consistent pattern emerged: loop-associated genes are enriched for regulatory, signalling, and cellular coordination processes, consistent with the proposed role of chromatin loops in enabling dynamic, context-dependent gene expression.

In *E. scolopes* whole-embryo data, loop-associated genes were enriched for processes related to cell adhesion and hormone-mediated signalling, including responses to steroids and lipids (Fig. S12A). Analysis of earlier embryonic stages further supported this, with stage 25 loop genes showing strong enrichment for cell–cell adhesion, as well as calcium ion binding (Fig. S22), consistent with the onset of tissue maturation and increased intercellular coordination during late organogenesis[12]. In *S. officinalis*, whole-embryo loop genes showed enrichment for processes such as protein transport, regulation of signal transduction and cell communication, as well as components of nuclear protein-containing complexes and ubiquitin transferase activity (Fig. S12B), suggesting loop-mediated control over intracellular trafficking and post-translational regulation. In *O. bimaculoides*, loop genes in whole embryos were strongly associated with GTPase signalling, cytoskeletal organisation, and calmodulin-dependent kinase activity, with enrichment of dynein, microtubule-associated, and actin-binding proteins (Fig. S12C), suggesting that chromatin loops may support structural remodeling and rapid signalling in complex, highly active tissues such as the *O. bimaculoides* nervous system.

Tissue-level GO enrichment analyses in *S. officinalis* (Fig. S23) and *O. bimaculoides* (Fig. S24) reinforced whole-embryo patterns and revealed additional tissue-specific specialisations. In *S. officinalis*, central brain loops were consistent with the regulatory and signalling enrichments seen at the whole-embryo level, while the eyes and internal organs showed more restricted enrichment, including cellular organisation and replication-related terms. In *O. bimaculoides*, loop genes in neural tissues (central brain, eyes and optic lobes, and arm crown) were consistently enriched for functions similar to those associated with whole-embryo samples, supporting a role for loops in neural structure and signal transduction. On the other hand, internal organ loops were instead enriched for ion transport and transmembrane transporter

activity, alongside protein-containing and channel-associated complexes, with additional signals related to transcriptional regulation. These findings suggest that chromatin loops in neural tissues are functionally geared toward plasticity and dynamic regulation, whereas those in internal organs are associated with essential cellular maintenance.

Taken together, these findings suggest that chromatin loops frequently bring together genes involved in plastic, condition-sensitive regulatory functions. These functional profiles stand in contrast to those of genes in gene pairs grouped by interaction category. While genes located in loops, which are rarely conserved, were enriched for dynamic regulatory processes, genes involved in conserved interactions across all species were primarily associated with core housekeeping functions. This dichotomy supports a model in which conserved gene interactions maintain essential, stable cellular processes under strong regulatory constraint, whereas chromatin loops act as flexible, evolutionarily adaptable regulatory elements that facilitate precise control of gene expression in response to developmental, spatial, or environmental cues.

Furthermore, we found that across all species, developmental stages, and tissues, zinc finger-containing genes comprised ~ 7–14% of loop-associated gene sets, while cadherin genes generally remained below 2%, with notable exceptions (Fig. S13). In *E. scolopes*, zinc finger gene representation peaked in stage 20 loops, whereas cadherin gene content increased steadily through development, reaching ~ 8.5% at stage 29. In *S. officinalis*, zinc finger gene proportions were lower (3–5%), but consistently higher than cadherins. In *O. bimaculoides*, the proportion of loop-associated zinc finger genes were notably high (~ 14%) across all tissues, consistent with a known lineage-specific expansion of this gene family in Octopodiformes[13], and not necessarily driven by chromatin interaction patterns.

No significant enrichment of zinc finger genes was detected across loop datasets in any species compared to the rest of the genome ($P > 0.05$, two-sided Fisher's exact test with BH correction). However, despite the overall low abundance of cadherin genes, enrichment analysis revealed significant overrepresentation in several categories. Cadherin genes were significantly enriched in the *E. scolopes* stage 25 and stage 29 loop sets (BH-adjusted $P = 2.22 \times 10^{-4}$ and $P = 1.22 \times 10^{-6}$, respectively), and also in the *S. officinalis* arm crown loop set (BH-adjusted $P = 0.0273$), indicating stage- or tissue-specific functional relevance. These findings are consistent with stage 25 and 29 representing key periods of nervous system development in *E. scolopes*[12]. The *O. bimaculoides* arm crown loops also showed the highest proportion of cadherin genes across tissues, consistent with the pattern found in *S. officinalis*, although the enrichment was not statistically significant. The high proportion of cadherin genes in the *S. officinalis* and *O. bimaculoides* arm crown loops likely reflects the neural character of the arm tissues in both species, which support complex sensorimotor functions. Given the conserved role of cadherins in nervous system development across metazoans[14] and the expansion of protocadherins in the Octopodiformes[13] and Decapodiformes[15,16], their enrichment in loop-

associated regions of neural tissues likely reflects their involvement in establishing and maintaining complex neural architecture in the coleoids.

Developmental stage and tissue-specific motif enrichment at loop anchors mostly mirrored patterns from whole-embryo analyses (main text), including consistent significant (hypergeometric test, BH-adjusted $P < 0.05$) detection of low-complexity and repeat-associated sequences, and widespread enrichment of Forkhead, bHLH, MYB, Homeobox, and CTCF-like motifs across species. Building on this shared regulatory foundation, tissue-specific analyses revealed additional transcription factors reflecting context-dependent regulatory activity. In *O. bimaculoides*, the eyes and optic lobes were enriched for neural regulators such as Barhl2, Vax2, and NeuroG2, while central brain, internal organs, and the arm crown displayed more general motifs including Tcf3 and GA-rich sequences. In *S. officinalis*, all tissues showed additional enrichment for developmental regulators such as Vax2 and Dlx1. These findings suggest that while some regulatory motifs shape a conserved regulatory backbone, dynamic recruitment of context-specific transcription factors likely contributes to the spatial and temporal control of gene expression.

Supplementary Note 5. *Chromatin loops are enriched for CNEs and open chromatin regions and show greater plasticity than TADs*

We quantified CNE enrichment in loop anchors and loop interior regions across *E. scolopes*, *O. bimaculoides*, and *S. officinalis*. Loop anchors showed significant enrichment for conserved CNEs in all three species compared to background genomic regions (BH-adjusted two-sided Wilcoxon rank-sum test, $P = 6.6 \times 10^{-4}$ for *E. scolopes* CNEs conserved in *O. bimaculoides*, 4.0×10^{-10} for *O. bimaculoides* CNEs conserved in *E. scolopes*, and 1.2×10^{-13} for *S. officinalis* CNEs conserved in *E. scolopes*). Loop interior regions also exhibited significant CNE enrichment compared to the genomic background, although to a lesser extent in *E. scolopes* and *O. bimaculoides* (BH-adjusted $P = 5.4 \times 10^{-3}$ and 2.7×10^{-2}, respectively), and very strongly in *S. officinalis* (BH-adjusted $P = 1.6 \times 10^{-61}$), potentially reflecting the shorter evolutionary distance between decapodiform–decapodiform CNEs relative to octopodiform–decapodiform CNEs. Direct comparisons of loop anchors and loop interior regions showed that CNE density was significantly higher in loop anchors in *E. scolopes* and *O. bimaculoides* (BH-adjusted $P = 1.6 \times 10^{-2}$ and 4.4×10^{-8}, respectively), and marginally higher in *S. officinalis* (BH-adjusted $P = 5.4 \times 10^{-2}$). Together, our results suggest that chromatin loop topology, especially the anchors, which form the spatial proximity-based interactions between distal genomic regions, plays a regulatory role in coleoid genomes.

To further assess how chromatin loops relate to gene content, we compared the proportion of loop regions occupied by genes to genome-wide gene density. *E. scolopes* loops showed a median gene density of 19.1%, while *O. bimaculoides* loops showed a median gene density of 23.3% between loop anchors. These values contrast with genome-wide gene pairs, where median gene density ranged from 33.0% to 46.2% (Supplementary Data 5). These differences

were statistically significant between loops and gene pairs (two-sided Wilcoxon rank-sum test, BH-adjusted $p < 0.0001$), confirming that loops are consistently more gene- sparse. The higher gene content in *O. bimaculoides* is consistent with its more compact genome, which has a smaller size despite having a similar number of annotated genes to *E. scolopes*[13,15].

Next, we quantified normalised ATAC peak enrichment in loop anchors, loop interior regions, and the genomic background at stages 20, 25, and 29. At all three developmental stages, loop anchor regions showed significantly greater chromatin accessibility than background regions (two-sided Wilcoxon rank-sum test, BH-adjusted P = 0.0092 at stage 20, 0.0092 at stage 25, and 9.0×10^{-8} at stage 29). At stage 29, loop interior regions were also significantly enriched for ATAC peaks (P = 0.010) compared to background regions, though this enrichment was not observed at stages 20 or 25. Pairwise comparisons further revealed that loop anchors had significantly higher accessibility than loop interior regions at all stages (BH-adjusted P = 0.035 at stage 20, 0.022 at stage 25, and 0.0063 at stage 29).

Median normalised fractional chromatin accessibility at loop anchors increased across development, from 0.559 at stage 20 to 0.630 at stage 25 and 0.634 at stage 29. Background regions, including loop interior, showed a similar upward trend, indicating a general increase in chromatin accessibility during embryogenesis. However, loop anchors exhibited a broader distribution of chromatin accessibility than all other genomic regions, suggesting that only a subset of anchors become highly accessible during development.

To test whether loops indeed represent more dynamic regulatory structures than higher-order chromatin features, we performed TAD calling and differential TAD analysis across developmental stages and tissues. In contrast to loops, TADs appeared far more conserved; approximately 65–75% of TADs were shared across tissue comparisons (Fig. S25A). Most differential events were classified as complex rearrangements, where existing TADs were neither split nor merged but reorganised into entirely new configurations[17], potentially enabling localised expression shifts while preserving broader architectural domains. Nevertheless, the general patterns of tissue-specific differences observed in loops and TADs were broadly consistent, suggesting that both levels of genome architecture contribute to tissue-specific regulation, albeit with differing degrees of conservation. UpSet plots indicated that the same set of TADs were recurrently identified as differential across tissues, suggesting a shared group of domains with heightened regulatory plasticity. However, this pattern may partly reflect our analytical approach, which classifies TADs absent in a tissue as differential (Fig. S25B). Taken together, these results indicate that chromatin loops represent plastic, context-dependent regulatory features of genome organisation, potentially facilitating fine-scale control of gene expression. Furthermore, they may evade the evolutionary constraints experienced by whole TADs, which serve as more stable, higher-order structures.

Supplementary References

1. Simakov, O. *et al.* Deeply conserved synteny and the evolution of metazoan chromosomes. *Sci Adv* **8**, eabi5884 (2022).
2. Rogers, T. F. *et al.* Gene modelling and annotation for the Hawaiian bobtail squid, Euprymna scolopes. *Sci. Data* **11**, 40 (2024).
3. Thiffeault, J.-L. & Budisic, M. Braidlab: A software package for braids and loops. *arXiv [math.GT]* (2014).
4. Farré, M., Robinson, T. J. & Ruiz-Herrera, A. An Integrative Breakage Model of genome architecture, reshuffling and evolution: The Integrative Breakage Model of genome evolution, a novel multidisciplinary hypothesis for the study of genome plasticity. *Bioessays* **37**, 479–488 (2015).
5. Liao, Y., Zhang, X., Chakraborty, M. & Emerson, J. J. Topologically associating domains and their role in the evolution of genome structure and function in Drosophila. *Genome Res.* **31**, 397–410 (2021).
6. Mantica, F. *et al.* Evolution of tissue-specific expression of ancestral genes across vertebrates and insects. *Nat. Ecol. Evol.* **8**, 1140–1153 (2024).
7. Jiang, W. & Chen, L. Tissue specificity of gene expression evolves across mammal species. *J. Comput. Biol.* **29**, 880–891 (2022).
8. Drummond, D. A., Bloom, J. D., Adami, C., Wilke, C. O. & Arnold, F. H. Why highly expressed proteins evolve slowly. *Proc. Natl. Acad. Sci. U. S. A.* **102**, 14338–14343 (2005).
9. Pál, C., Papp, B. & Hurst, L. D. Highly expressed genes in yeast evolve slowly. *Genetics* **158**, 927–931 (2001).
10. Eisenberg, E. & Levanon, E. Y. Human housekeeping genes, revisited. *Trends Genet.* **29**,

569–574 (2013).

11. Albertin, C. B. & Katz, P. S. Evolution of cephalopod nervous systems. *Curr. Biol.* **33**, R1087–R1091 (2023).

12. Lee, P. N., Callaerts, P. & de Couet, H. G. The embryonic development of the Hawaiian bobtail squid (Euprymna scolopes). *Cold Spring Harb. Protoc.* **2009**, db.ip77 (2009).

13. Albertin, C. B. *et al.* The octopus genome and the evolution of cephalopod neural and morphological novelties. *Nature* **524**, 220–224 (2015).

14. Hulpiau, P. & van Roy, F. Molecular evolution of the cadherin superfamily. *Int. J. Biochem. Cell Biol.* **41**, 349–369 (2009).

15. Belcaid, M. *et al.* Symbiotic organs shaped by distinct modes of genome evolution in cephalopods. *Proc. Natl. Acad. Sci. U. S. A.* **116**, 3030–3035 (2019).

16. Rencken, S. *et al.* Chromosome-scale genome assembly of the European common cuttlefish Sepia officinalis. *Genomics* (2025).

17. Cresswell, K. G. & Dozmorov, M. G. TADCompare: An R package for differential and temporal analysis of topologically associated domains. *Front. Genet.* **11**, 158 (2020).
