# Supplementary material for: Genome reorganisation and expansion shape 3D genome architecture and define a distinct regulatory landscape in coleoid cephalopods
Source: Nat Commun. 2026 Oct 9;17:10319. doi: 10.1038/s41467-026-77625-7 (PMC13652810; doi:10.1038/s41467-026-77625-7)
Supplement: Supplementary file 2 [file 41467_2026_77625_MOESM2_ESM.pdf]

<u>Description Of Additional Supplementary Information</u>

Supplementary Data 1. Summary of all Micro-C sequencing libraries used in this study.

Supplementary Data 2. Sequencing depth and mapping summary for Micro-C libraries used in this study.

Supplementary Data 3. Differential tissue-specific expression in *E. scolopes* across gene pair categories.

Supplementary Data 4. Tissue specificity of gene expression (τ) across interaction categories and chromosomal origin.

Supplementary Data 5. Intervening gene density across gene pairs in *E. scolopes* and *O. bimaculoides*, grouped by interaction status.

Supplementary Data 6. Differential tissue-specific expression in *E. scolopes* across gene pair categories and *P. maximus* chromosomal origin.

Supplementary Data 7. Median genomic distances and statistical comparisons between gene pairs across interaction statuses, grouped by P. maximus chromosomal origin and genomic distance categories for the three focal coleoid species.

Supplementary Data 8. Enriched known DNA motifs in loop anchor-associated sequences in *E. scolopes*.

Supplementary Data 9. Enriched known DNA motifs in loop anchor-associated sequences in *S. officinalis*.

Supplementary Data 10. Enriched known DNA motifs in loop anchor-associated sequences in *O. bimaculoides*.

Supplementary Data 11. Enriched known DNA motifs in the CRISPR-cas9 knockout region in *E. berryi*.

Supplementary Data 12. CRISPR–Cas9 guide RNAs used for targeted knockout of the intronic region of Quaking B in E. berryi.
