# Supplementary material for: Genome reorganisation and expansion shape 3D genome architecture and define a distinct regulatory landscape in coleoid cephalopods
Source: Nat Commun. 2026 Oct 9;17:10319. doi: 10.1038/s41467-026-77625-7 (PMC13652810; doi:10.1038/s41467-026-77625-7)
Supplement: Supplementary file 3 [file 41467_2026_77625_MOESM4_ESM.pdf]

nature portfolio

Corresponding author(s): Thea F. Rogers

Last updated by author(s): 23.11.25

Reporting Summary

Nature Portfolio wishes to improve the reproducibility of the work that we publish. This form provides structure for consistency and transparency in reporting. For further information on Nature Portfolio policies, see our Editorial Policies and the Editorial Policy Checklist.

Statistics

For all statistical analyses, confirm that the following items are present in the figure legend, table legend, main text, or Methods section.

n/a	Confirmed	
☐	☒	The exact sample size (*n*) for each experimental group/condition, given as a discrete number and unit of measurement
☐	☒	A statement on whether measurements were taken from distinct samples or whether the same sample was measured repeatedly
☐	☒	The statistical test(s) used AND whether they are one- or two-sided<br>*Only common tests should be described solely by name; describe more complex techniques in the Methods section.*
☒	☐	A description of all covariates tested
☐	☒	A description of any assumptions or corrections, such as tests of normality and adjustment for multiple comparisons
☐	☒	A full description of the statistical parameters including central tendency (e.g. means) or other basic estimates (e.g. regression coefficient) AND variation (e.g. standard deviation) or associated estimates of uncertainty (e.g. confidence intervals)
☐	☒	For null hypothesis testing, the test statistic (e.g. *F*, *t*, *r*) with confidence intervals, effect sizes, degrees of freedom and *P* value noted<br>*Give P values as exact values whenever suitable.*
☒	☐	For Bayesian analysis, information on the choice of priors and Markov chain Monte Carlo settings
☒	☐	For hierarchical and complex designs, identification of the appropriate level for tests and full reporting of outcomes
☐	☒	Estimates of effect sizes (e.g. Cohen's *d*, Pearson's *r*), indicating how they were calculated

Our web collection on statistics for biologists contains articles on many of the points above.

Software and code

Policy information about availability of computer code

Data collection	Micro-C libraries were prepared using the Dovetail Micro-C kit and sequenced at core facilities using standard Illumina instruments. Sequencing was performed on NovaSeq S4 XP flow cells (PE150), NovaSeqX 10B XP flow cells (PE150), and one NextSeq550 run (PE75, Medium output).
Data analysis	All software used for data analysis is fully described in the Methods. Briefly, analyses used BLASTN v2.16.0, TransDecoder v5.7.1, miniprot v0.12-r237, cd-hit v4.8.1, odp v0.3.0, Trimmomatic v0.39, seqtk v1.4, HiC-Pro v3.0.0, JuicerTools v1.22.01, Juicebox v2.15, Plotgardener v1.8.3, BEDTools v2.30.0, FAN-C v0.9.2, Mustache v1.0.1, HOMER v5.1, RepeatModeler v2.0.6, RepeatMasker v4.1.8, STAR v2.7.11b, Trim Galore v0.6.10, featureCounts v2.1.1, DESeq2 v1.48.1, and additional tools including UCSC utilities v486 and deepTools v3.5.0. Analyses were conducted in Python v3.10 and R (v4.3–4.5) with packages including dplyr, tidyr, stringr, ggplot2, pheatmap, VennDiagram, TADCompare, UpSet, clusterProfiler, AnnotationForge, and others. Custom Python, R, and Bash scripts used throughout the analysis will be sent as a github link to the editor to share with reviewers and will be made publically availble prior to publication.

For manuscripts utilizing custom algorithms or software that are central to the research but not yet described in published literature, software must be made available to editors and reviewers. We strongly encourage code deposition in a community repository (e.g. GitHub). See the Nature Portfolio guidelines for submitting code & software for further information.

Data

Policy information about availability of data

All manuscripts must include a data availability statement. This statement should provide the following information, where applicable:

- Accession codes, unique identifiers, or web links for publicly available datasets
- A description of any restrictions on data availability
- For clinical datasets or third party data, please ensure that the statement adheres to our policy

Data availability
aw Micro-C sequencing data generated in this study have been deposited in the NCBI Sequence Read Archive under BioProject accession PRJNA1450934.
Sample names can be found in Table S1.
Most code is available on github here: https://github.com/TheaFrances/Coleoid_genome_topology_2025
Code used for dot plot visualisations is available at: https://bitbucket.org/viemet/public/src/master/vamp/, and code for multi-locus topology analysis (MLT) is available at https://github.com/conchoecia/odp/commit/8310442e49a1b26c6cf9b0b6b672ca40779311b9

Research involving human participants, their data, or biological material

Policy information about studies with human participants or human data. See also policy information about sex, gender (identity/presentation), and sexual orientation and race, ethnicity and racism.

Reporting on sex and gender	*Use the terms sex (biological attribute) and gender (shaped by social and cultural circumstances) carefully in order to avoid confusing both terms. Indicate if findings apply to only one sex or gender; describe whether sex and gender were considered in study design; whether sex and/or gender was determined based on self-reporting or assigned and methods used. Provide in the source data disaggregated sex and gender data, where this information has been collected, and if consent has been obtained for sharing of individual-level data; provide overall numbers in this Reporting Summary. Please state if this information has not been collected. Report sex- and gender-based analyses where performed, justify reasons for lack of sex- and gender-based analysis.*
Reporting on race, ethnicity, or other socially relevant groupings	*Please specify the socially constructed or socially relevant categorization variable(s) used in your manuscript and explain why they were used. Please note that such variables should not be used as proxies for other socially constructed/relevant variables (for example, race or ethnicity should not be used as a proxy for socioeconomic status). Provide clear definitions of the relevant terms used, how they were provided (by the participants/respondents, the researchers, or third parties), and the method(s) used to classify people into the different categories (e.g. self-report, census or administrative data, social media data, etc.) Please provide details about how you controlled for confounding variables in your analyses.*
Population characteristics	*Describe the covariate-relevant population characteristics of the human research participants (e.g. age, genotypic information, past and current diagnosis and treatment categories). If you filled out the behavioural & social sciences study design questions and have nothing to add here, write "See above."*
Recruitment	*Describe how participants were recruited. Outline any potential self-selection bias or other biases that may be present and how these are likely to impact results.*
Ethics oversight	*Identify the organization(s) that approved the study protocol.*

Note that full information on the approval of the study protocol must also be provided in the manuscript.

Field-specific reporting

Please select the one below that is the best fit for your research. If you are not sure, read the appropriate sections before making your selection.

☒ Life sciences ☐ Behavioural & social sciences ☐ Ecological, evolutionary & environmental sciences

For a reference copy of the document with all sections, see nature.com/documents/nr-reporting-summary-flat.pdf

Life sciences study design

All studies must disclose on these points even when the disclosure is negative.

Sample size	Sample sizes were determined by the availability of high-quality Micro-C libraries and embryo material for each species and developmental stage. No statistical method was used to pre-determine sample size. The number of samples per species, developmental stage, and tissue type is reported in Table S1 and is standard for chromatin conformation studies.
Data exclusions	No data were excluded except where explicitly stated in the Methods (e.g., exclusion of bins lacking genes, removal of scaffolds, filtering of outliers presumed to originate from misassemblies). All exclusion criteria were pre-established and applied uniformly across species.
Replication	Multiple biological replicates were generated for most Micro-C sequencing libraries across species, developmental stages, and tissues (Table S1). All replicates were assessed for sequencing quality, mapping rate, library complexity, and coverage. Only replicates that passed these quality filters and were suitable for cross-species or cross-stage comparisons were included in downstream analyses.

	Libraries with substantially higher coverage were downsampled to match the lowest high-quality replicate for analyses that compared across samples. Mapping and downsampling stats are reported in table S2.<br>All computational analyses were repeated on the available high-quality replicates where possible, and results were consistent across replicates.
Randomization	Randomization was not applicable. Samples (embryos or tissues) were assigned to experimental processing based on developmental stage, species, and availability, not on experimental grouping. No treatment conditions or group assignments requiring randomization were part of the study design.
Blinding	Blinding was not relevant because no treatment groups were compared and data collection involved sequencing-based assays processed using standardized computational pipelines.

Reporting for specific materials, systems and methods

We require information from authors about some types of materials, experimental systems and methods used in many studies. Here, indicate whether each material, system or method listed is relevant to your study. If you are not sure if a list item applies to your research, read the appropriate section before selecting a response.

Materials & experimental systems

n/a	Involved in the study
☒	☐ Antibodies
☒	☐ Eukaryotic cell lines
☒	☐ Palaeontology and archaeology
☐	☒ Animals and other organisms
☒	☐ Clinical data
☒	☐ Dual use research of concern
☒	☐ Plants

Methods

n/a	Involved in the study
☒	☐ ChIP-seq
☒	☐ Flow cytometry
☒	☐ MRI-based neuroimaging

Animals and other research organisms

Policy information about studies involving animals; ARRIVE guidelines recommended for reporting animal research, and Sex and Gender in Research

Laboratory animals	The study involved cephalopod animals (Euprymna scolopes, Sepia officinalis, Octopus bimaculoides, and Euprymna berryi) maintained and handled in laboratory aquaria. Adult animals were kept under controlled seawater conditions, and embryos were collected, maintained, and dissected according to established institutional protocols. Developmental stages used included whole embryos (stages 20, 25, 29 for E. scolopes; stage 29 for S. officinalis; stages 20 and 29 for O. bimaculoides and E. berryi).
Wild animals	*Provide details on animals observed in or captured in the field; report species and age where possible. Describe how animals were caught and transported and what happened to captive animals after the study (if killed, explain why and describe method; if released, say where and when) OR state that the study did not involve wild animals.*
Reporting on sex	Cephalopod embryos do not exhibit externally distinguishable sexual characteristics, and sex cannot be determined at the developmental stages examined. Therefore, sex-based analyses were not applicable and were not performed.
Field-collected samples	Adult cephalopods were obtained from established laboratory colonies or marine facilities; no animals were collected directly from the wild by the authors for this study.
Ethics oversight	Ethics statement<br>All animal husbandry and experimental procedures complied with the relevant institutional, national, and international regulations governing the use of cephalopods in research. Work involving S. officinalis at the Max Planck Institute for Brain Research was conducted under institutional project licence F126/1025 in accordance with Directive 2010/63/EU. Work involving E. scolopes at Schönbrunn Zoo and the University of Vienna complied with Directive 2010/63/EU and AAALAC guidelines and was conducted under veterinary import permits 2023-0.555.058 and 2021-0.400.840. Work involving O. bimaculoides at the University of Chicago was approved by the Institutional Animal Care and Use Committee (IACUC) under protocol #72760. Procedures involving O. bimaculoides and E. berryi at the Marine Biological Laboratory were approved by the MBL Institutional Animal Care and Use Committee (IACUC) under protocol 22-13I. Embryonic cephalopods are exempt from Directive 2010/63/EU, which applies to independently feeding post-hatching animals.

Note that full information on the approval of the study protocol must also be provided in the manuscript.

Plants

Seed stocks	*Report on the source of all seed stocks or other plant material used. If applicable, state the seed stock centre and catalogue number. If plant specimens were collected from the field, describe the collection location, date and sampling procedures.*
Novel plant genotypes	*Describe the methods by which all novel plant genotypes were produced. This includes those generated by transgenic approaches, gene editing, chemical/radiation-based mutagenesis and hybridization. For transgenic lines, describe the transformation method, the number of independent lines analyzed and the generation upon which experiments were performed. For gene-edited lines, describe the editor used, the endogenous sequence targeted for editing, the targeting guide RNA sequence (if applicable) and how the editor was applied.*
Authentication	*Describe any authentication procedures for each seed stock used or novel genotype generated. Describe any experiments used to assess the effect of a mutation and, where applicable, how potential secondary effects (e.g. second site T-DNA insertions, mosiacism, off-target gene editing) were examined.*
