# Supplementary material for: Genome reorganisation and expansion shape 3D genome architecture and define a distinct regulatory landscape in coleoid cephalopods
Source: Nat Commun. 2026 Oct 9;17:10319. doi: 10.1038/s41467-026-77625-7 (PMC13652810; doi:10.1038/s41467-026-77625-7)
Supplement: Supplementary file 4 [file 41467_2026_77625_MOESM5_ESM.pdf]

nature portfolio

Peer Review File

Genome reorganisation and expansion shape 3D genome architecture and define a distinct regulatory landscape in coleoid cephalopods

Corresponding Author: Dr Thea Rogers

This file contains all reviewer reports in order by version, followed by all author rebuttals in order by version.

Version 0:

Reviewer comments:

Reviewer #1

(Remarks to the Author)
In "Genome reorganisation and expansion shape 3D genome architecture and define a distinct regulatory landscape in coleoid cephalopods", Rogers and co-authors present a multi-species, multi-tissue, multi-stage characterisation of the 3D genome regulatory landscape and its evolution in cephalopods. Cephalopods have highly rearranged and expanded genomes compared with other molluscs, which co-occurs with the evolution of complex morphological, anatomical and behavioural characters in this group. Here the authors explore how high order chromatin architecture might have contributed to macrosyntenic changes in these molluscs and the emergence of their unique biological characters. They identify dynamic regulatory loops enriched in genes involved in nervous system development and transcriptional regulation, and through Cas9-mediated knockouts, demonstrate that at least some of these regulatory regions might be essential for normal development.

While I think the findings are important and provide novel insights into the evolution and particularities of cephalopod and animal genomes, I find several major points that I believe could help the manuscript be clearer and more accessible:

- The text is dense and at points difficult to follow and I am not sure combining Results and Discussion, while having seven supplementary notes, makes it easy for the reader. I suggest simplifying the text and perhaps separating results and discussion, so that findings are described in a more succinct way (ideally incorporating the key elements of the supplementary notes, see below) and all interpretations are together in a single section.

- The use of the term compartment is confusing. Do they refer to compartments as in vertebrate genomes (A/B compartments) or to TADs? The authors use TADs in some parts of the text, some panel figures and in the code. I suggest being consistent with existing terminology and if the authors consider that the organisation they observe in cephalopods does not fully align with either compartments and TADs, then it might be better to use a different term (e.g., domains), to avoid confusion.

- Are Supplementary Notes needed? These are again dense and they seem to include important observations. Could they be included, somehow, into the main text but focused to the key points?

- Could the authors provide aggregate plots of compartments and loops to easily illustrate insulation/enrichment? The identified loops are also long (Mb-range). Although cephalopod genomes are large, these are also very long loops. Could this be due to the resolutions used to call loops (50 and 100 kb)? Have the authors tried to identify loops at lower resolutions? Does their data allow them to check lower resolutions? If they have and there aren't smaller loops, that would also be an important finding.

- CRISPR-Cas9. It is unclear how the authors selected the 1Kb target region. Do they have ATAC-seq data indicating that there are potential accessible regulatory elements there? The authors state that the deletion was confirmed by PCR and genotyping. While they show the PCR in figure S28, it is unclear what they mean by genotyping (did they sequence the bands to confirm the deletions observed by PCR?)

(Remarks on code availability)
The code seems sufficient for ensuring reproducibility.

Reviewer #2

(Remarks to the Author)
The manuscript by Rogers et al. presents a detailed comparative analysis of the 3D genome architecture of three coleoid cephalopod lineages. The authors generated an extensive collection of sequencing datasets spanning multiple species, developmental stages, and tissues, and they complemented these data with a CRISPR-Cas9 knock-out experiment to test the functional role of regulatory elements. Their results reveal a hierarchical organization of regulatory restraint across the coleoid 3D genome. At one end of this hierarchy, large topological compartments have been maintained despite extensive genome rearrangements and often contain housekeeping genes. In contrast, chromatin loops are highly dynamic and often species-specific. Overall, I enjoyed reading this manuscript and find the results to be novel and an advancement of our understanding of the evolutionary implications of the 3D genome. I found their conclusions to be supported by the data they presented. Most of my comments are about the overall readability and clarity of the manuscript.
1. My primary concern with the manuscript is that the large number of supplementary notes and figures occasionally makes the main text difficult to follow. At times, some of the context found in the supplementary notes seems important for interpreting the conclusions being drawn within the main text. For example, some of the functional enrichment analyses have interesting results but they are only mentioned in concluding sentences. The dichotomy of gene categories found to be enriched in loops vs conserved interactions (supplementary note 4) I found to be especially interesting but hardly mentioned in the main text. I also thought figure S31 was helpful for summarizing all your results. I understand that there are constraints on manuscript length/number of figures, but incorporating some of these details could improve overall clarity.
2. Fig 2A: What are the grey vs orange dots in the dot plots? Some details in methods or figure legend on how this plot was generated would be helpful.
3. Page 4, first paragraph: Need clarification on what it means for O. bimaculoides segments to have homology with multiple E. scolopes regions. Are these regions duplicated in squid?
4. Page 4, second paragraph: I found it was difficult to understand the methodology of this section, and I had to read it several times. More explanation (in methods or main text) about the CNE comparisons would improve clarity.
5. It was not clear to me why P. maximus was used as the outgroup when Nautilus has a high-quality genome. Additionally, since P. maximus is used it would be helpful to include it in Fig 1D. and to note the number of chromosomes it has since chromosomal fusions are an important part of the story.
6. Fig 3A, expression is measured in E. scolopes. For the genes pairs found only interacting in O. bimaculoides, would it be possible that gene expression is more highly correlated if measured in octopus?
7. Page 6, last sentence: I thought an annotation for Sepia was generated?
8. Page 6, last sentence: Can you briefly define what you mean by gene coverage? Could be confused with depth.
9. Page 7, first full sentence: Is there a typo? Should it say “significantly lower”?
10. Table S6: fix headers
11. Page 14, last paragraph: Typo, 5C should be 5D.

(Remarks on code availability)

Version 1:

Reviewer comments:

Reviewer #1

(Remarks to the Author)
The authors have done a great job addressing all my recommendations. I think the manuscript is now clearer and a solid contribution to the study of 3D genome architecture in coleids and animals in general.

(Remarks on code availability)
The repo appears complete

Reviewer #2

(Remarks to the Author)
The authors have resolved my comments about the overall clarity of the manuscript. The conclusions are supported by their detailed analyses.

(Remarks on code availability)
The code is well documented and is a useable resource to the community.

Open Access This Peer Review File is licensed under a Creative Commons Attribution 4.0 International License, which permits use, sharing, adaptation, distribution and reproduction in any medium or format, as long as you give appropriate credit to the original author(s) and the source, provide a link to the Creative Commons license, and indicate if changes were made.
In cases where reviewers are anonymous, credit should be given to 'Anonymous Referee' and the source.
The images or other third party material in this Peer Review File are included in the article's Creative Commons license, unless indicated otherwise in a credit line to the material. If material is not included in the article's Creative Commons license and your intended use is not permitted by statutory regulation or exceeds the permitted use, you will need to obtain permission directly from the copyright holder.
To view a copy of this license, visit https://creativecommons.org/licenses/by/4.0/

We thank the reviewers for their careful reading of the manuscript and for their constructive and insightful comments. We have revised the manuscript accordingly and believe the changes have significantly improved the clarity of the manuscript. Below we address each comment in detail. All changes in the revised manuscript and in this response are indicated using red text.

Reviewer 1

In "Genome reorganisation and expansion shape 3D genome architecture and define a distinct regulatory landscape in coleoid cephalopods", Rogers and co-authors present a multi-species, multi-tissue, multi-stage characterisation of the 3D genome regulatory landscape and its evolution in cephalopods. Cephalopods have highly rearranged and expanded genomes compared with other molluscs, which co-occurs with the evolution of complex morphological, anatomical and behavioural characters in this group. Here the authors explore how high order chromatin architecture might have contributed to macrosyntenic changes in these molluscs and the emergence of their unique biological characters. They identify dynamic regulatory loops enriched in genes involved in nervous system development and transcriptional regulation, and through Cas9-mediated knockouts, demonstrate that at least some of these regulatory regions might be essential for normal development.

While I think the findings are important and provide novel insights into the evolution and particularities of cephalopod and animal genomes, I find several major points that I believe could help the manuscript be clearer and more accessible:

- The text is dense and at points difficult to follow and I am not sure combining Results and Discussion, while having seven supplementary Notes, makes it easy for the reader. I suggest simplifying the text and perhaps separating results and discussion, so that findings are described in a more succinct way (ideally incorporating the key elements of the supplementary Notes, see below) and all interpretations are together in a single section.

Response: We thank the reviewer for this helpful suggestion. We agree that improving clarity and accessibility of the manuscript is important, and we have revised the text accordingly. To ensure that the central findings and interpretations are more directly accessible to the reader, we have revised several sections of the Results and Discussion. We have also moved Supplementary Fig. S31 into the main text (now Table 1), as it provides a clear summary of the key findings and relationships described throughout the manuscript. In addition, the Supplementary Notes have been simplified, with key elements from Notes 6 and 7 incorporated into the main text in a simplified form and removed from the Supplementary Notes:

Page 12–13, lines 465–486 (previously Supplementary Note 6): "This loop is present at stage 20 but significantly downregulated at stages 25 and 29 (Fig. 4D). At stage 20, the loop coincides with elevated chromatin accessibility (ATAC-seq peaks), local insulation at loop borders (Fig. 4E), and peak gene expression (Fig. 4F), suggesting it coordinates the activation of this regulatory module during the onset of tissue differentiation. The loop brings together six genes; Prefoldin, two subunits of Chromatin Assembly Factor 1 (CAF-1), the snRNA-activating complex (SNAPc), Sas10/Utp3/C1D, and Sirtuin, all of which are functionally linked to chromatin assembly, cytoskeletal organisation, and RNA processing,

processes essential for early development. Each of these genes has well-established developmental roles: Prefoldin facilitates the folding of cytoskeletal proteins required for morphogenesis and cellular proliferation[45]; CAF-1 subunits organise chromatin and deposit histones to preserve epigenetic states during cell proliferation[46]; SNAPc regulates snRNA transcription necessary for pre-mRNA splicing during zygotic genome activation[47]. Sas10/Utp3/C1D proteins are involved in small subunit rRNA processing and ribosome biogenesis[48,49]; and Sirtuin modulates chromatin accessibility and epigenetic silencing during developmental patterning and cell fate specification[50,51]. Together, their activity likely orchestrates key transitions during early organogenesis. By stage 25, the disappearance of this loop is accompanied by a reduction in both insulation and expression, consistent with a shift from specification to more stable lineage commitment. Notably, while SNAPc (cluster_11478) and one CAF-1 subunit (cluster_18817) are syntenic in the outgroup *P. maximus* genome, the remaining genes are dispersed across the same or different chromosomes in *P. maximus*. This suggests that the looped configuration is a synapomorphic feature, likely facilitated by genome rearrangements in the coleoid ancestor, providing a regulatory context that enabled the emergence of cephalopod novelties such as their unique developmental program."

Page 15, lines 555–566 (previously Supplementary Note 7): "To investigate the potential regulatory role of the ~ 1 kb intronic sequence deleted by CRISPR–Cas9 (Fig. 5B), we performed motif enrichment analysis using HOMER (Table S11). After correction, a Hox-like motif (Hoxb4) was significantly enriched (BH-corrected $P < 0.05$), consistent with input from homeodomain transcription factors involved in body patterning and neural development[59]. Additional metazoan motifs, including Lhx6 (LIM-homeobox), bHLH factors, and IRF-associated motifs, were detected at nominal levels, suggesting diverse regulatory inputs also associated with neural and developmental processes[60–63]. Notably, MYNN (Myoneurin) motifs[64] were present within the deleted sequence and in an adjacent intronic region conserved in *O. bimaculoides* (Fig. 5B), which lies within a repetitive interval not targetable by guide RNAs. This suggests that the targeted region forms part of a broader regulatory module, with the deleted sequence representing one functional component. Together, these findings support the interpretation that the targeted intronic region represents a putative regulatory element, likely contributing to neural and developmental gene regulation."

Page 16, lines 596–613 (previously Supplementary Note 7): "Given the phenotypic and transcriptional consequences of the knockout, we infer that the deleted region integrates regulatory inputs critical for neural gene expression and development. These results suggest that the intronic sequence acts as a regulatory hub, contributing to the expression of genes within its chromatin interaction network. Long-range effects of the knock-out are consistent with a meta-loop architecture, in which regulatory elements exert influence across chromatin domain boundaries and over large genomic distances[65]. The downregulated genes span both conserved and lineage-specific targets, suggesting evolutionary flexibility in regulatory wiring despite structural conservation of the core loop. The more limited expression changes observed within the conserved loop, compared to the stronger transcriptional effects on genes beyond the loop, suggest that genes embedded within highly conserved and likely regulatorily entangled loop topologies are more buffered against perturbation of single CNEs, whereas genes involved in more distal and less conserved regulatory interactions show stronger transcriptional responses when regulatory regions are disrupted. Supporting this, the corresponding loop in *O. bimaculoides* shows tighter co-localisation between enhancer and promoter, suggesting additional regulatory entanglement following chromosomal fusion and mixing in the Octopodiformes. Together,

these results support a model in which conserved loop topologies retain core regulatory functions while allowing flexibility in their gene targets over evolutionary time. Figure 5H illustrates this scenario, showing how chromatin loops can mediate long-range regulatory interactions that are constrained and evolvable at the same time."

The remaining supplementary materials contain detailed analyses across multiple comparison categories and are retained in the Supplementary Information to avoid overloading the main text.

With respect to the suggestion to separate Results and Discussion, we have carefully considered this but have retained a combined Results and Discussion structure. Given the multi-layered nature of the analyses and the three interconnected themes developed throughout the manuscript (general evolutionary history of the coleoids, deeply conserved chromatin domains, and dynamic chromatin loops), we found that integrating results with interpretation improves clarity and allows the narrative to build more cohesively. We have, however, revised the text to ensure that results and their interpretation are presented as clearly and succinctly as possible.

- The use of the term compartment is confusing. Do they refer to compartments as in vertebrate genomes (A/B compartments) or to TADs? The authors use TADs in some parts of the text, some panel figures and in the code. I suggest being consistent with existing terminology and if the authors consider that the organisation they observe in cephalopods does not fully align with either compartments and TADs, then it might be better to use a different term (e.g., domains), to avoid confusion.

Response: We thank the reviewer for raising this point and agree that the terminology could be clearer. In the revised manuscript we have standardised the terminology used to describe chromatin organisation. Specifically, we now use the term "chromatin domain" to refer to regions containing interacting gene pairs associated with regions of high insulation, and the term TAD only to refer to regions identified using TAD-calling algorithms. We have removed the use of the term "compartment" so it can not be confused with A/B compartments, and clarified the terminology in the manuscript:

Page 5–6, lines 201–203: "Throughout this manuscript, we use the term chromatin domain to describe genomic regions bordered by high insulation, and reserve the term TAD only for regions identified using TAD-calling algorithms[36]."

- Are Supplementary Notes needed? These are again dense and they seem to include important observations. Could they be included, somehow, into the main text but focused to the key points?

Response: We have simplified and moved some parts of the Supplementary Notes, and Figure S31 into the main text, and only kept some of the very technical aspects (such as additional comparative and validation figures or braiding analysis) in the Supplementary Notes (see response above).

- Could the authors provide aggregate plots of compartments and loops to easily illustrate insulation/enrichment?

Response: We thank the reviewer for this suggestion. However, we prefer to keep these analyses separate because they address distinct biological questions and are based on different analytical frameworks. Specifically, we implemented insulation analysis for regions with shared orthologs to infer changes in and conservation of chromatin structures across species. This analysis was thus ortholog pair centered, and showed general conservation of chromatin domains in coleoid cephalopods. Further investigation of this conservation landscape in the context of chromatin loops, on the other hand, revealed that the loops are more dynamic and show little conservation in the coleoids. Because these analyses operate on different genomic features and scales, combining them into aggregate plots would obscure these distinct patterns. We have therefore retained separate analyses.

We specify this on **page 9–10, lines 350–359**: "Across species, only a minority of gene-associated loops overlapped with gene pairs from the four interaction categories described above. Approximately 5% of loops contained gene pairs in conserved interactions (i.e. those interacting across the coleoids, or restricted to interacting across the Decapodiformes or in *O. bimaculoides,* combined) while ~ 1% overlapped with gene pairs not assigned to any interaction category. Because the four gene pair categories were restricted to intrachromosomal orthologs shared across all three coleoid species and the outgroup *P. maximus*, they represent evolutionarily conserved interaction potential. This suggests that most chromatin loops are not formed around deeply conserved orthologous genes, but may instead reflect novel regulatory configurations involving genes that are scattered across different chromosomes in other species or recently evolved genes."

- The identified loops are also long (Mb-range). Although cephalopod genomes are large, these are also very long loops. Could this be due to the resolutions used to call loops (50 and 100 kb)? Have the authors tried to identify loops at lower resolutions? Does their data allow them to check lower resolutions? If they have and there aren't smaller loops, that would also be an important finding.

Response: We thank the reviewer for raising this point. Loop detection in Hi-C and Micro-C data is resolution dependent, and smaller loops can be detected when loop calling is performed at higher resolution. We had previously explored loop calling at 25 kb resolution. As expected, this resolution identifies additional shorter-range loops in the datasets where sufficient signal is available (see table below). However, reliable loop detection at this scale requires very high sequencing depth, and comparable loop detection across species becomes less reliable when sequencing depth differs between datasets. For example, in *E. scolopes* no loops were detected at 25 kb resolution, whereas numerous additional shorter loops were detected in the *S. officinalis* and *O. bimaculoides* datasets. For this reason, we focused our primary analyses on loops detected at 50 kb and 100 kb resolution, which provided robust and comparable loop detection across all species. Although absolute loop sizes decrease at higher resolution, the relative pattern across species is consistent: loops in *O. bimaculoides* remain larger on average than those in the decapodiform genomes across both 100 kb and 50 kb resolutions.

The large genomic distances spanned by many loops are particularly relevant in the context of this study. Since coleoid cephalopod genomes have undergone extensive expansion and chromosomal rearrangement, this has resulted in unusually large genomic intervals between regulatory elements and their target genes. Long-range chromatin loops therefore provide an important mechanism by

which regulatory interactions can be maintained despite large-scale genome reorganisation. Identifying and characterising these long-range interactions is therefore particularly relevant for understanding how regulatory architecture is maintained and reorganised in these genomes.

We have modified the text to clarify the choice of resolution for loop calling:

Page 24, lines 905–908: "Loop size estimates depend on the resolution at which loops are detected, with higher resolutions enabling the identification of shorter-range loops. The 50 kb and 100 kb resolutions were therefore chosen as they provided robust loop detection while allowing consistent comparisons across species."

Sample	Number of loops detected at 100 kb resolution	Average loop size at 100 kb resolution (Mb)	Number of loops detected at 50 kb resolution	Average loop size at 50 kb resolution (Mb)	Number of loops detected at 25 kb resolution	Average loop size at 25 kb resolution (Mb)
E. scolopes **whole embryo stage 29**	274	4.30	63	0.983	0	NA
S. officinalis **whole embryo stage 29**	511	3.32	479	1.25	286	0.565
O. bimaculoides **whole embryo stage 20**	415	5.09	263	1.77	557	0.677

- CRISPR-Cas9. It is unclear how the authors selected the 1Kb target region. Do they have ATAC-seq data indicating that there are potential accessible regulatory elements there? The authors state that the deletion was confirmed by PCR and genotyping. While they show the PCR in figure S28, it is unclear what they mean by genotyping (did they sequence the bands to confirm the deletions observed by PCR?)

Response: We thank the reviewer for these questions.

The targeted region was not selected based on ATAC-seq signal. Instead, it was chosen based on multiple independent lines of biological evidence together with practical constraints on guide RNA design.

First, the region lies within a chromatin loop that is conserved across coleoid species and contains genes that are upregulated in neural tissues and involved in neural development and regulatory processes. Second, a conserved non-coding sequence is present within the same intron of Quaking B across these species. Based on this combination of conserved chromatin topology, neural gene expression, and sequence conservation, we selected this intron as a candidate region for CRISPR–Cas9 perturbation.

Guide RNAs were designed to span a ~1 kb region within this locus. This region was positioned within the conserved decapodiform sequence and as close as possible to the conserved region shared with *O. bimaculoides*. Such conserved non-coding elements are rare across coleoid genomes, and intronic regions are highly repetitive and often difficult to target with CRISPR without predicted off-target effects. The final target region therefore represented the closest location within this conserved intronic segment where guide RNAs could be designed without predicted off-target effects.

We have revised the Methods section to clarify this point:

Page 27, lines 1020–1027: "Notably, a conserved sequence is present in the same intron of Quaking B in all three species. Therefore, we selected this intron as a candidate region for CRISPR–Cas9 perturbation. Guide RNAs were designed to span a ~ 1 kb region within this locus. This region was positioned within the conserved decapodiform sequence and as close as possible to the conserved region shared with *O. bimaculoides*, while ensuring that guide RNAs could be designed without predicted off-target effects. Based on this combination of shared chromatin topology, brain-specific expression, sequence conservation, and guide feasibility within a highly repetitive genome, we selected this region for CRISPR–Cas9 knockout to test its regulatory function."

For genotyping, the target region in wild type and knockout embryos was amplified using PCR. The different-sized bands were then gel purified and analysed with Sanger sequencing to confirm successful deletion. We have modified the main text and Materials and Methods to better explain this procedure.

Page 15, lines 553–554: "The deletion was confirmed by PCR (Fig. S28A, B) and genotyping" was changed to "The deletion was confirmed by genotyping, using PCR amplification of the target region followed by Sanger sequencing (Fig. S28A, B)."

Page 29, lines 1080–1082: "Wild-type and knockout bands were gel purified and analysed using Sanger Sequencing services provided by Genewiz (South Plainfield, NJ) to confirm successful deletion of the target region."

Reviewer 2

The manuscript by Rogers et al. presents a detailed comparative analysis of the 3D genome architecture of three coleoid cephalopod lineages. The authors generated an extensive collection of sequencing datasets spanning multiple species, developmental stages, and tissues, and they complemented these data with a CRISPR-Cas9 knock-out experiment to test the functional role of regulatory elements. Their results reveal a hierarchical organization of regulatory restraint across the coleoid 3D genome. At one end of this hierarchy, large topological compartments have been maintained despite extensive genome rearrangements and often contain housekeeping genes. In contrast, chromatin loops are highly dynamic and often species-specific. Overall, I enjoyed reading this manuscript and find the results to be novel and an advancement of our understanding of the evolutionary implications of the 3D genome. I found their conclusions to be supported by the data they presented. Most of my comments are about the overall readability and clarity of the manuscript.

1. My primary concern with the manuscript is that the large number of supplementary notes and figures occasionally makes the main text difficult to follow. At times, some of the context found in the supplementary notes seems important for interpreting the conclusions being drawn within the main text. For example, some of the functional enrichment analyses have interesting results but they are only mentioned in concluding sentences. The dichotomy of gene categories found to be enriched in loops vs conserved interactions (supplementary note 4) I found to be especially interesting but hardly mentioned in the main text. I also thought figure S31 was helpful for summarizing all your results. I understand that there are constraints on manuscript length/number of figures, but incorporating some of these details could improve overall clarity.

Response: We thank the reviewer for this helpful and constructive comment. We agree that some important context was contained within the Supplementary Notes, which could make interpretation of the main text more difficult. To address this, we have revised the manuscript to incorporate key findings from the supplementary material directly into the main text. In particular, we have moved Supplementary Fig. S31 into the main text (now Table 1), as it provides a clear summary of the key findings and relationships described throughout the manuscript. The final column of Table 1 also highlights the contrast in functional enrichment between gene categories associated with chromatin loops and those associated with conserved interactions (described in Supplementary Note 4). The Supplementary Notes have been simplified, Notes 6 and 7 have been removed, and key elements from these have been incorporated into the main text in a simplified form:

Page 12–13, lines 465–486 (previously Supplementary Note 6): "This loop is present at stage 20 but significantly downregulated at stages 25 and 29 (Fig. 4D). At stage 20, the loop coincides with elevated chromatin accessibility (ATAC-seq peaks), local insulation at loop borders (Fig. 4E), and peak gene expression (Fig. 4F), suggesting it coordinates the activation of this regulatory module during the onset of tissue differentiation. The loop brings together six genes; Prefoldin, two subunits of Chromatin Assembly Factor 1 (CAF-1), the snRNA-activating complex (SNAPc), Sas10/Utp3/C1D, and Sirtuin, all of which are functionally linked to chromatin assembly, cytoskeletal organisation, and RNA processing, processes essential for early development. Each of these genes has well-established developmental roles: Prefoldin facilitates the folding of cytoskeletal proteins required for morphogenesis and cellular proliferation[45]; CAF-1 subunits organise chromatin and deposit histones to preserve epigenetic states during cell proliferation[46]; SNAPc regulates snRNA transcription necessary for pre-mRNA splicing during zygotic genome activation[47]. Sas10/Utp3/C1D proteins are involved in small subunit rRNA processing and ribosome biogenesis[48,49]; and Sirtuin modulates chromatin accessibility and epigenetic silencing during developmental patterning and cell fate specification[50,51]. Together, their activity likely orchestrates key transitions during early organogenesis. By stage 25, the disappearance of this loop is accompanied by a reduction in both insulation and expression, consistent with a shift from specification to more stable lineage commitment. Notably, while SNAPc (cluster_11478) and one CAF-1 subunit (cluster_18817) are syntenic in the outgroup *P. maximus* genome, the remaining genes are dispersed across the same or different chromosomes in *P. maximus*. This suggests that the looped configuration is a synapomorphic feature, likely facilitated by genome rearrangements in the coleoid ancestor, providing a regulatory context that enabled the emergence of cephalopod novelties such as their unique developmental program."

Page 15, lines 555–566 (previously Supplementary Note 7): "To investigate the potential regulatory role of the ~ 1 kb intronic sequence deleted by CRISPR–Cas9 (Fig. 5B), we performed motif enrichment analysis using HOMER (Table S11). After correction, a Hox-like motif (Hoxb4) was significantly enriched (BH-corrected $P < 0.05$), consistent with input from homeodomain transcription factors involved in body patterning and neural development[59]. Additional metazoan motifs, including Lhx6 (LIM-homeobox), bHLH factors, and IRF-associated motifs, were detected at nominal levels, suggesting diverse regulatory inputs also associated with neural and developmental processes[60–63]. Notably, MYNN (Myoneurin) motifs[64] were present within the deleted sequence and in an adjacent intronic region conserved in *O. bimaculoides* (Fig. 5B), which lies within a repetitive interval not targetable by guide RNAs. This suggests that the targeted region forms part of a broader regulatory module, with the deleted sequence representing one functional component. Together, these findings support the interpretation that the targeted intronic region represents a putative regulatory element, likely contributing to neural and developmental gene regulation."

Page 16, lines 596–613 (previously Supplementary Note 7): "Given the phenotypic and transcriptional consequences of the knockout, we infer that the deleted region integrates regulatory inputs critical for neural gene expression and development. These results suggest that the intronic sequence acts as a regulatory hub, contributing to the expression of genes within its chromatin interaction network. Long-range effects of the knock-out are consistent with a meta-loop architecture, in which regulatory elements exert influence across chromatin domain boundaries and over large genomic distances[65]. The downregulated genes span both conserved and lineage-specific targets, suggesting evolutionary flexibility in regulatory wiring despite structural conservation of the core loop. The more limited expression changes observed within the conserved loop, compared to the stronger transcriptional effects on genes beyond the loop, suggest that genes embedded within highly conserved and likely regulatorily entangled loop topologies are more buffered against perturbation of single CNEs, whereas genes involved in more distal and less conserved regulatory interactions show stronger transcriptional responses when regulatory regions are disrupted. Supporting this, the corresponding loop in *O. bimaculoides* shows tighter co-localisation between enhancer and promoter, suggesting additional regulatory entanglement following chromosomal fusion and mixing in the Octopodiformes. Together, these results support a model in which conserved loop topologies retain core regulatory functions while allowing flexibility in their gene targets over evolutionary time. Figure 5H illustrates this scenario, showing how chromatin loops can mediate long-range regulatory interactions that are constrained and evolvable at the same time."

More broadly, we have revised several sections of the Results and Discussion to improve clarity and ensure that the main conclusions can be understood without reliance on the supplementary material.

2. Fig 2A: What are the grey vs orange dots in the dot plots? Some details in methods or figure legend on how this plot was generated would be helpful.

Response: We thank the reviewer for pointing this out. In Fig. 2A, each dot represents an orthologous gene. Orange dots indicate genes located on chromosome pairs inferred to be orthologous, while grey dots indicate genes located on other chromosome combinations. Chromosome pairs were considered putatively orthologous when the number of shared orthologous genes between the two chromosomes was significantly higher than expected by chance relative to the genomic background, as determined using Fisher's exact test ($P \leq 1 \times 10^{-5}$). We have clarified this in the figure legend:

Page 43, lines 1424–1427: "Each dot represents an orthologous gene. Orange dots indicate genes located on chromosome pairs inferred to be orthologous based on significant enrichment of shared orthologous genes (Fisher's exact test, $P \leq 1 \times 10^{-5}$), whereas grey dots indicate genes located on other chromosome combinations."

3. Page 4, first paragraph: Need clarification on what it means for O. bimaculoides segments to have homology with multiple E. scolopes regions. Are these regions duplicated in squid?

Response: Generally, one-to-many correspondences in synteny can be a result of either duplication of fusion. For cephalopods and, in this case squids, we do not find duplicated genes on these multiple segments that correspond to a single octopus region. This suggests fusion (and mixing) on the branch leading to octopus. We have added this logic to the end of the paragraph.

Page 4, lines 142–145: "This pattern can be explained either by duplication in the squid lineage or fusion-with-mixing in the octopus lineage. As we do not find any duplicated genes on the squid segments that correspond to a single octopus region, these results point to a high degree of within-chromosomal fusion-with-mixing in the Octopodiformes."

4. Page 4, second paragraph: I found it was difficult to understand the methodology of this section, and I had to read it several times. More explanation (in methods or main text) about the CNE comparisons would improve clarity.

Response: Thank you, we have reworked this paragraph to have better logic and flow:

Page 4–5, lines 147–164: "To quantify the effect of fusion-with-mixing on local sequence conservation, we analysed the lengths and genomic dispersion of sequence alignments between species. Dispersion is defined as the proportion of translocated regions from homologous to non-homologous chromosomes and correlates with evolutionary distance[35]. Between *E. scolopes* and *S. officinalis,* we found 150,610 and 110,440 total alignments (including both coding and non-coding sequences of 100 bp or longer, 78 Mb total alignment length) on homologous and non-homologous chromosomes, respectively (42.3% dispersion). Between *E. scolopes* and *O. bimaculoides*, 35,273 and 29,948 alignments (19 Mb total alignment length) were on homologous and non-homologous chromosomes, respectively (45.9% dispersion), showing a similar dispersion rate with the absolute numbers scaling with the genetic distance. Distinguishing between alignments that overlapped coding regions and those that did not (CNEs), we found that the dispersion rate for CNEs was higher, with only 3,185 alignments on homologous chromosomes, yet 22,462 alignments on non-homologous chromosomes for *E. scolopes* and *S. officinalis* (87.6% dispersion). Similarly, CNEs comprised only 528 alignments on homologous chromosomes compared to 6,182 alignments on non-homologous chromosomes for *E. scolopes* and *O. bimaculoides* (92.1% dispersion). This suggests that across coleoids, there are more relaxed constraints on the translocation of putative regulatory non-coding sequences compared with coding regions, likely due to their smaller size, modular function, and reduced sensitivity to disruption compared with protein-coding loci."

5. It was not clear to me why P. maximus was used as the outgroup when Nautilus has a high-quality genome. Additionally, since P. maximus is used it would be helpful to include it in Fig 1D. and to

note the number of chromosomes it has since chromosomal fusions are an important part of the story.

Response: We thank the reviewer for noting this. The scallop *Pecten maximus* was used as a non-coleoid molluscan reference genome in this study because it provides a well-curated chromosome-level assembly with high BUSCO completeness (> 95%) and represents one of the most stable genomic resources currently available for molluscan comparative analyses. Importantly, bivalve genomes such as *P. maximus* retain extensive ancestral molluscan linkage structure and therefore provide a useful baseline for assessing large-scale chromosomal reorganisation in derived molluscan lineages. Using such a conserved molluscan genome allows large-scale rearrangements in coleoids to be evaluated relative to this baseline organisation rather than relying on a single cephalopod lineage as the reference state.

While the genome of *Nautilus pompilius* represents an important resource for cephalopod evolution, a chromosome-scale assembly became available only after the initial stages of this project. In addition, the currently available assembly is flagged for potential contamination in NCBI, which may complicate large-scale synteny comparisons. For these reasons, we used *P. maximus* as a stable non-coleoid molluscan reference for the analyses presented here.

To clarify this point, we have revised the manuscript to state more clearly that genome rearrangements in coleoids are evaluated relative to conserved molluscan genome organisation rather than interpreted strictly as changes on the coleoid ancestral branch. We have also added *P. maximus* to Fig. 1A and D and stated the number of chromosomes for this species in the main text as suggested:

Page 7, lines 261–267: "To further investigate the relationship between chromatin interactions and ancestral coleoid genome reorganisation, we examined the chromosomal locations of orthologous gene pairs in *Pecten maximus*, a molluscan outgroup with a chromosome-scale genome (n = 19). *P. maximus* retains extensive ancestral molluscan linkage structure and therefore provides a useful baseline for evaluating genome reorganisation in the coleoids[35]. Patterns of chromosomal origin were consistent between *P. maximus* and *Nautilus pompilius* (Fig. S7), supporting its use as an appropriate reference for capturing genomic changes associated with coleoid genome evolution."

In addition, we note that large-scale chromosomal organisation is broadly consistent between *N. pompilius* and *P. maximus*. As shown in Supplementary Fig. S7, interacting gene pairs that are located on the same *N. pompilius* chromosome are also largely located on the same *P. maximus* chromosome, and gene pairs on different *N. pompilius* chromosomes are similarly separated in *P. maximus*. Across all interaction categories, 83–87% of gene pairs show consistent chromosome assignments between the two genomes (Fig. S7), indicating that the large-scale chromosomal organisation used as a reference in our analyses is broadly conserved and that our conclusions are not dependent on the choice of outgroup. We have added the concordance values to the legend of Fig. S7:

Supplementary Figures, page 7, Fig. S7 legend: "Across all interaction categories, 83–87% of gene pairs show concordant chromosome assignments between the two genomes, indicating broadly consistent large-scale chromosomal organisation between *N. pompilius* and *P. maximus*."

Finally, we note that biological material from *P. maximus* is readily accessible, facilitating future experimental validation and comparative genomic analyses, whereas sampling of *N. pompilius* species is more restricted due to conservation considerations. This further supports the use of *P. maximus* as a practical reference genome for ongoing and future work.

6. Fig 3A, expression is measured in E. scolopes. For the genes pairs found only interacting in O. bimaculoides, would it be possible that gene expression is more highly correlated if measured in octopus?

Response: We thank the reviewer for this suggestion. In principle, gene pairs interacting specifically in *O. bimaculoides* could show higher expression correlation if measured using octopus expression data. We had previously explored this possibility using the only publicly available *O. bimaculoides* RNA-seq dataset from an earlier octopus genome study (Albertin et al., 2015). The overall trend was broadly consistent with our results from *E. scolopes*: gene pairs interacting across all species showed the highest co-expression, followed by those interacting only in Decapodiformes, then those interacting only in *O. bimaculoides*, and finally gene pairs not in conserved coleoid interactions, with statistically significant differences between categories.

However, this dataset is not well suited for robust co-expression analysis in this context. The vast majority of tissues sequenced lack biological replicates, and genes show very low expression across samples, resulting in limited dynamic range for correlation estimates. In addition, the available samples originate from adult tissues that do not correspond to the embryonic tissues profiled by Micro-C, preventing reliable assessment of tissue-specific loop-associated expression in downstream analyses. For these reasons, we did not include the octopus RNA-seq dataset in the manuscript and instead used much more recent *E. scolopes* RNA-seq datasets, which include biological replicates and have much higher read coverage. Furthermore, the *E. scolopes* developmental RNA-seq dataset also includes samples that match the developmental stages used for the Micro-C experiments and is therefore appropriate for the downstream developmental-stage–specific loop analyses presented in the manuscript.

7. Page 6, last sentence: I thought an annotation for Sepia was generated?

Response: We thank the reviewer for raising this point. A complete gene annotation for *S. officinalis* was not available at the time these analyses were performed. To enable comparative analyses involving orthologous genes, we generated a provisional annotation by mapping *E. scolopes* proteins to the *S. officinalis* genome using Miniprot. This approach is sufficient for analyses that rely only on orthologous genes but does not capture *S. officinalis*-specific genes and therefore cannot provide a complete representation of gene content. As a result, it is not appropriate for analyses that require a comprehensive gene annotation, such as estimating gene coverage between genomic regions. Including *S. officinalis* in this analysis using the provisional annotation would therefore bias gene coverage estimates downward. For this reason, the gene coverage analyses were restricted to *E. scolopes* and *O. bimaculoides*, where high-quality gene annotations were available.

Page 7, line 247: We have changed the text from “In the two coleoid species with available gene annotations” to “In the two coleoid species with comprehensive gene annotations”.

8. Page 6, last sentence: Can you briefly define what you mean by gene coverage? Could be confused with depth.

Response: We thank the reviewer for pointing this out. Here, "gene coverage" refers to the proportion of the genomic interval between interacting gene pairs that overlaps annotated genes, providing a measure of local gene density rather than sequencing depth. We "changed gene coverage" to "gene density" throughout, and clarified the meaning of this in the revised text:

Page 7, lines 247–250: "In the two coleoid species with comprehensive gene annotations, *E. scolopes* and *O. bimaculoides*, interacting gene pairs consistently showed higher gene density between them (defined here as the proportion of the intervening genomic region occupied by annotated genes) than pairs not in conserved coleoid interactions."

9. Page 7, first full sentence: Is there a typo? Should it say "significantly lower"?

Response: Yes, thanks, we have fixed this.

10. Table S6: fix headers

Response: Thank you, we have fixed this.

11. Page 14, last paragraph: Typo, 5C should be 5D.

Response: Thanks, we have corrected this.
